# Stereoselective Synthesis and Structural Confirmation of All Four 8-Hydroxyhexahydrocannabinol Stereoisomers

**DOI:** 10.3390/molecules31020289

**Published:** 2026-01-13

**Authors:** Kei Ieuji, Kayo Nakamura, Hideyo Takahashi

**Affiliations:** Faculty of Pharmaceutical Sciences, Tokyo University of Science, 6-3-1 Niijuku, Katsushika-ku, Tokyo 125-8585, Japan

**Keywords:** hexahydrocannabinol, cannabinoid, 8-hydroxyhexahydrocannabinol, metabolite, hydroboration, epoxidation, ^1^H NMR

## Abstract

Hexahydrocannabinol (HHC), a hydrogenated derivative of Δ^9^-tetrahydrocannabinol (Δ^9^-THC), is a semi-synthetic cannabinoid marketed as an alternative to Δ^9^-THC. Its hydroxylated metabolite, 8-hydroxyhexahydrocannabinol (8-OH-HHC), exists as four stereoisomers: (6a*R*,8*R*,9*R*,10a*R*), (6a*R*,8*S*,9*S*,10a*R*), (6a*R*,8*S*,9*R*,10a*R*), and (6a*R*,8*R*,9*S*,10a*R*). However, the lack of reference standards has hindered pharmacokinetic and forensic studies. This work reports the first stereoselective synthesis and structural confirmation of all four 8-OH-HHC stereoisomers. Two strategies were employed: hydroboration–oxidation and epoxidation–reduction. Hydroboration of Δ^8^-THC with BH_3_·THF followed by oxidation predominantly produced *anti*-isomers (6a*R*,8*R*,9*R*,10a*R*) and (6a*R*,8*S*,9*S*,10a*R*) in moderate yields, along with small amounts of *syn*-isomer (6a*R*,8*S*,9*R*,10a*R*), suggesting an atypical mechanistic pathway. In contrast, *syn*-isomers (6a*R*,8*S*,9*R*,10a*R*) and (6a*R*,8*R*,9*S*,10a*R*) were accessed via epoxidation of Δ^8^-THC acetate using *m*CPBA and subsequent reduction with NaBH_3_CN/BF_3_·OEt_2_, affording the desired products with moderate selectivity. Absolute configurations were confirmed by nuclear Overhauser effect spectroscopy (NOESY). These methods will facilitate future pharmacokinetic and forensic research and support the development of improved detection strategies.

## 1. Introduction

**Figure 1:**
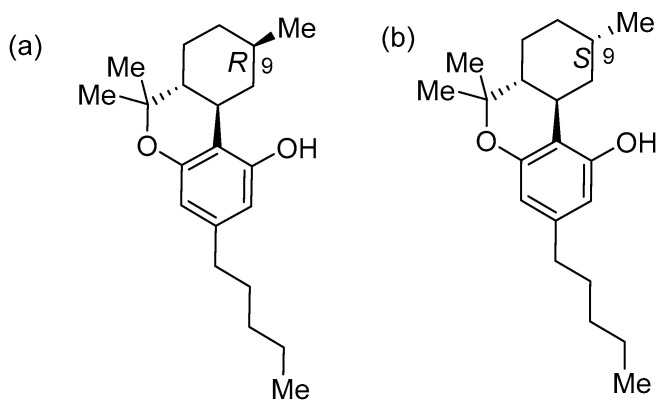
Structures of HHC epimers: (**a**) (6a*R*,9*R*,10a*R*)-HHC; (**b**) (6a*R*,9*S*,10a*R*)-HHC.

Preparations of cannabis, including the flowering or fruiting tops of the cannabis plant and their resins, are widely used as recreational drugs. Globally, 4.1% of individuals aged 15–64 had used cannabis within the past year [1]. Cannabinoids are the main components of cannabis, with more than 100 types reported to date [2]. Among them, Δ^9^-tetrahydrocannabinol (Δ^9^-THC) is a psychoactive narcotic and has become the most frequently consumed illicit drug of abuse in the world [3]. Recently, a new cannabinoid, hexahydrocannabinol (HHC), a hydrogenated derivative of Δ^9^-THC, has entered the market as an alternative to Δ^9^-THC [4,5]. Although HHC has been reported to occur naturally [6], most of the currently available HHC products are obtained through relatively simple chemical transformations of natural cannabinoids. As HHC is directly produced by the catalytic hydrogenation of Δ^8^-THC or Δ^9^-THC [7], currently marketed HHC is typically a mixture of the epimers, namely (6a*R*,9*R*,10a*R*)-HHC and (6a*R*,9*S*,10a*R*)-HHC (Figure 1) [4,8].

**Figure 2:**
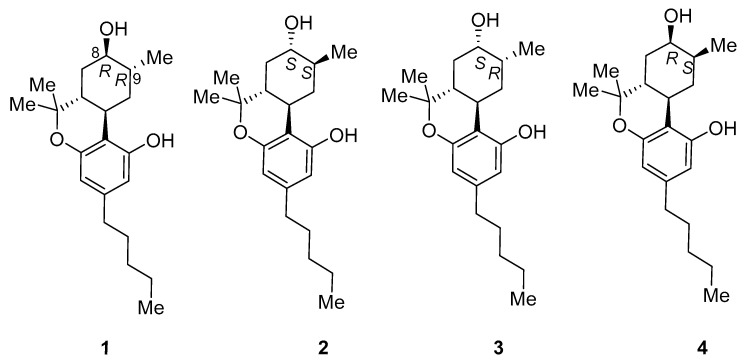
Structures of (8*R*)OH-(6a*R*,9*R*,10a*R*)-HHC (**1**), (8*S*)OH-(6a*R*,9*S*,10a*R*)-HHC (**2**), (8*S*)OH-(6a*R*,9*R*,10a*R*)-HHC (**3**), (8*R*)OH-(6a*R*,9*S*,10a*R*)-HHC (**4**).

HHC metabolites have recently captured the interest of toxicology researchers and forensic analysts. Pharmacokinetic studies of HHC have identified several metabolites, among which 8-OH-HHC, in which the C8-position of HHC is hydroxylated, has been reported since 1990 [9,10,11]. Considering that commercially available HHC typically consists of a mixture of (6a*R*,9*R*,10a*R*)-HHC and (6a*R*,9*S*,10a*R*)-HHC, the resulting 8-OH-HHC metabolites are expected to form a mixture comprising (8*R*)OH-(6a*R*,9*R*,10a*R*)-HHC (**1**), (8*S*)OH-(6a*R*,9*S*,10a*R*)-HHC (**2**), (8*S*)OH-(6a*R*,9*R*,10a*R*)-HHC (**3**), and (8*R*)OH-(6a*R*,9*S*,10a*R*)-HHC (4) (Figure 2). In 2025, Gréen reported that three stereoisomers of 8-OH-HHC (**1**, **2**, **4**) were detected in urine specimens submitted for cannabinoid testing in varying amounts. However, compound **3** was not identified at the time, as a reference standard was not available for confirmation [12].

Reliable analysis of 8-OH-HHC requires reference standards for all four of its stereoisomers. Therefore, we decided to stereoselectively synthesize the four stereoisomers of 8-OH-HHC (**1**, **2**, **3**, **4**). Our synthetic strategy uses Δ^8^-THC as the starting material to provide the stereoisomers through two routes: hydroboration–oxidation or epoxidation–reduction.

## 2. Results and Discussion

### 2.1. Synthesis of (8R)OH-(6aR,9R,10aR)-HHC (***1***) and (8S)OH-(6aR,9S,10aR)-HHC (***2***)

For the synthesis of the *anti*-stereoisomers (**1**, **2**), we decided to examine the hydroboration reaction of Δ^8^-THC [13]. The starting material, Δ^8^-THC, was synthesized according to known synthetic methods [14]. Following the procedure reported by Mechoulam [13], 2 equivalents of BH_3_·THF were added to Δ^8^-THC in THF solution at 23 °C for 22 h. This reaction mixture was treated with excess H_2_O_2_ in a basic aqueous solution. NMR analysis revealed that the resulting crude product was a mixture of three compounds. Purification of the crude product by silica gel column chromatography afforded compound **1** in an isolated yield of 26% and compound **2** in a yield of 34%. The third compound could not be isolated because it co-eluted with compound **2**, but NMR analysis revealed it to be compound **3**. Considering that the hydroboration reaction proceeds via *syn*-addition, the formation of the *anti*-compounds **1** and **2** was expected. However, the appearance of compound **3** was unexpected, suggesting an atypical pathway. This observation prompted the further investigation of the hydroboration reaction conditions. Therefore, we conducted a systematic study of the effects of the hydroboration–oxidation reaction conditions on the production ratio of the three compounds (Table 1). First, Δ^8^-THC was treated with 2 equivalents of BH_3_·THF at −20 °C for 2 h (reaction 1). Then, 10 equivalents of hydrogen peroxide and 10 equivalents of sodium hydroxide were added to carry out an oxidation reaction (reaction 2) for 1 h. The resulting crude product was separated by silica-gel column chromatography to give compound **1** in 24% yield, along with compounds **2** and **3** as a mixture. The ratio of compounds **2** and **3** was determined by nuclear magnetic resonance (NMR) spectroscopy to be 5:1 (entry 1).

The reaction temperature was raised to −10 °C, and the reaction was continued for 21 h, yielding 21% of compound **1** and 34% of the mixture of compounds **2** and **3**, with a 4:1 ratio (entry 2). The temperature was further increased, and the hydroboration reaction was carried out at 0 °C for 2 h. As a result, the yield of **1** was increased to 40%, and the yield of the mixture of **2** and **3** was 35%, with the ratio of **2** to **3** being 7:1 (entry 3). Attempts to suppress the formation of *syn*-compound **3** by increasing the amount of BH_3_·THF, raising the temperature, or increasing the amounts of hydrogen peroxide and sodium hydroxide were unsuccessful. The persistent formation of *syn*-compound **3** suggests that the reaction may proceed via a mechanism that deviates from the generally accepted one [15].

**Figure 3:**
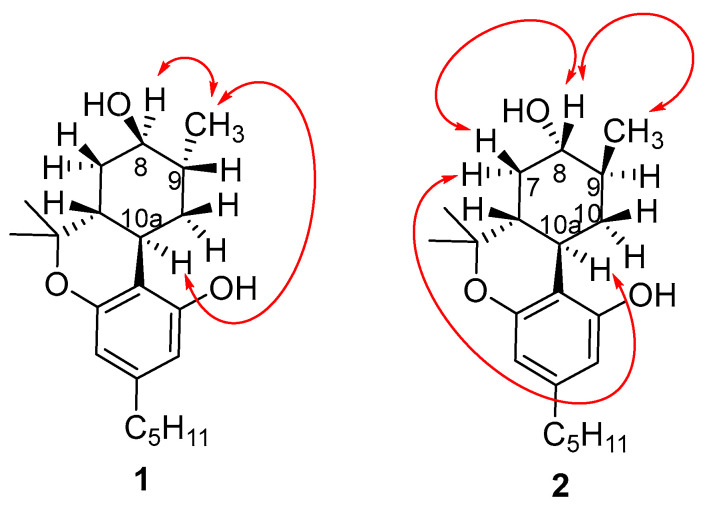
Key correlations of NOESY spectrum of (8*R*)OH-(6a*R*,9*R*,10a*R*)-HHC (**1**) and (8*S*)OH-(6a*R*,9*S*,10a*R*)-HHC (**2**).

The stereochemical structures of compounds **1** and **2** were determined by nuclear Overhauser effect spectroscopy (NOESY) (Figure 3).

In compound **1**, correlations were observed between the 8-H proton signal and the 9-methyl signal; the 9-methyl signal was also correlated with the 10a-H proton signal. These results revealed that the 8-OH group and the 9-methyl group were in the *trans*-configuration, confirming that the absolute configuration of compound **1** was (8*R*,9*R*). As to compound **2**, correlations were observed between the 9-methyl signal and the 8-H proton signal, which was also correlated with one of the 7-H signals. Additionally, another 7-H proton signal was found to be correlated with the 10a-H proton signal. These results revealed that the 8-OH group and the 9-methyl group were in the *trans*-configuration, confirming that the absolute configuration of compound **2** was (8*S*,9*S*). Furthermore, the structure of compound **3** was consistent with the (8*S*,9*R*) isomer obtained in the synthesis described below.

**Scheme 1:**
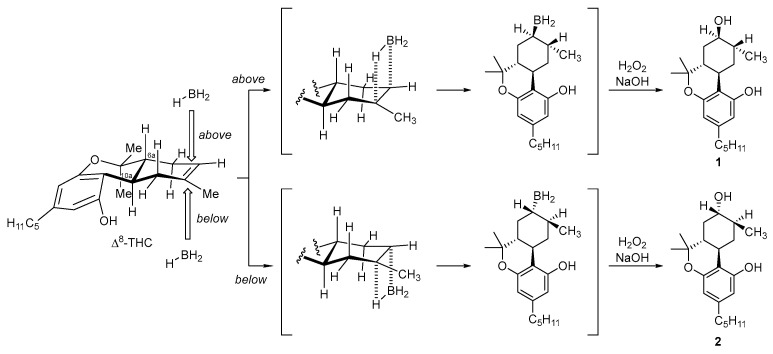
Reaction mechanism of hydroboration to yield compounds **1** and **2**.

The mechanism by which compounds **1** and **2** are obtained is shown in Scheme 1. According to the well-known hydroboration mechanism, the *syn*-addition of BH_3_ occurs above or below the double bond. Considering that compounds **1** and **2** were obtained in approximately the same ratio, the addition reaction is likely to proceed with approximately the same probability from either above or below the double bond. The lack of pronounced stereoselectivity in this hydroboration reaction can be attributed to the fact that the chiral sources 6a-H and 10a-H are far from the double bond and therefore are not expected to be sterically affected.

**Scheme 2:**
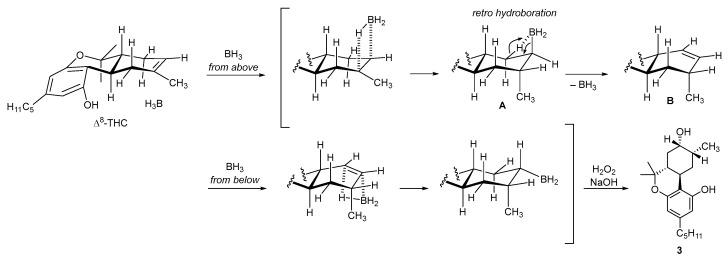
Reaction mechanism of hydroboration to yield compound **3**.

In contrast, the formation of compound **3** is inconsistent with the conventional *syn*-addition mechanism of hydroboration. In this context, the report by Merino and Fernández-Herrera (2020) [16], which described the generation of the *trans*-hydroboration products of β5 steroids, is particularly informative. They proposed that the *trans*-hydroboration products arise via a hydroboration–retro hydroboration mechanism. Our results can be rationalized by invoking a similar mechanism (Scheme 2). According to the conventional mechanism, a *syn*-addition reaction proceeds to form intermediate **A**, which subsequently undergoes retro hydroboration to form intermediate **B**. If the hydroboration of intermediate **B** proceeds from the face below the double bond, as shown in Scheme 2, then oxidation should give compound **3**. It should be noted that *syn*-addition to obtain intermediate B can occur in four possible ways, depending on whether the addition occurs from the face above or below the double bond and whether or not it follows Markovnikov’s rule. In light of this mechanistic complexity, the formation of by-products other than compound **3** cannot be excluded, although such species have not yet been isolated.

### 2.2. Synthesis of (8S)OH-(6aR,9R,10aR)-HHC (***3***), (8R)OH-(6aR,9S,10aR)-HHC (***4***)

**Scheme 3:**
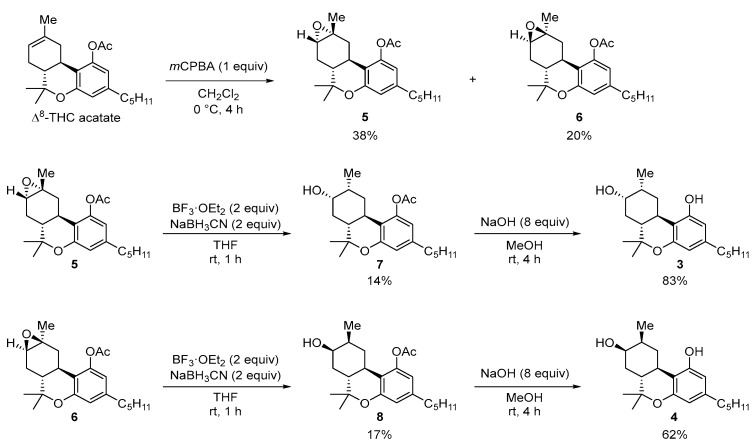
Synthesis of syn-stereoisomers **3**, **4**.

For the synthesis of the *syn*-stereoisomers (**3**, **4**), we modified the Dethe method [17] and investigated the regioselective ring-opening of epoxides using a combination of NaBH_3_CN and BF_3_⋅OEt_2_ (Scheme 3). Δ^8^-THC acetate, which was prepared from Δ^8^-THC according to the conventional method [18], was treated with **1** equivalent of *m*CPBA at 0 °C in CH_2_Cl_2_ for 4 h to afford epoxide (**5**) in 38% yield and epoxide (**6**) in 20% yield [19].

The epoxy derivative **5** was reduced with 2 equivalents of NaBH_3_CN in the presence of 2 equivalents of BF_3_⋅OEt_2_ in THF solution at 23 °C for 1 h to provide a mixture. After purification of the crude compounds by silica-gel column chromatography, compound (**7**) was isolated in 14% yield as a single diastereoisomer. Compound **7** was then hydrolyzed with sodium hydroxide to give the desired *syn*-compound **3** in 83% yield. Similarly, the epoxy derivative **6** was reduced using the NaBH_3_CN/BF_3_⋅OEt_2_ system to provide a mixture, which was purified by silica-gel column chromatography, and compound (**8**) was isolated in 17% yield as a single diastereoisomer. The hydrolysis of compound **8** afforded the corresponding *syn*-compound **4** in 62% yield. Hence, the reduction of compounds **5** and **6** yielded many products, suggesting that the regio- and stereo-selectivity of this reaction was insufficient.

**Figure 4:**
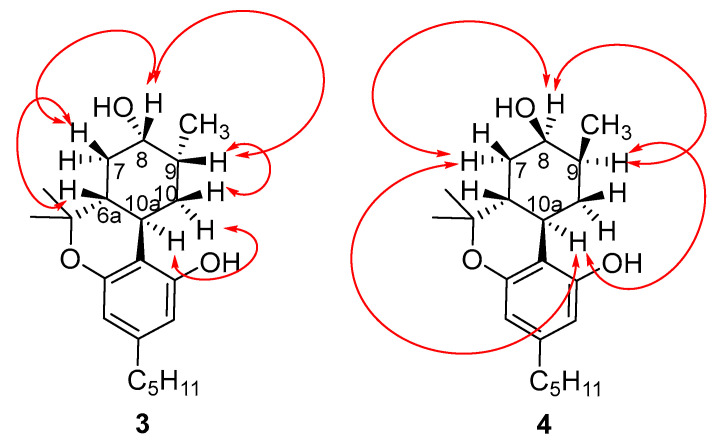
Key correlations of the NOESY spectrum of (8*S*)OH-(6a*R*,9*R*,10a*R*)-HHC (**3**) and (8*R*)OH-(6a*R*,9*S*,10a*R*)-HHC (**4**).

The stereochemical structures of compounds **3** and **4** were determined by the NOESY spectrum (Figure 4).

In compound **3**, correlations were observed between the 9-H proton signal and the 8-H proton signal, which was also correlated with one of the 7-H proton signals. The 7-H proton signal was also correlated with the 6a-H proton signal. Additionally, correlations were observed between the 9-H proton signal and one of the 10-H proton signals, and between the other 10-H proton signal and the 10a-H proton signal. These results revealed that the 8-OH group and the 9-methyl group were in the *cis* configuration, confirming that the absolute configuration of compound **3** was (8*S*,9*R*). In compound **4**, correlations were observed between the 8-H and 9-H proton signals; the 9-H signal was also correlated with the 10a-H proton signal. Additionally, correlations were observed between the 8-H proton signal and that of the 7-H proton signal, which was also correlated with the 10a-H proton signal. These results revealed that the 8-OH group and the 9-methyl group were in the *cis* configuration, confirming that the absolute configuration of compound **4** was (8*R*,9*S*).

**Scheme 4:**
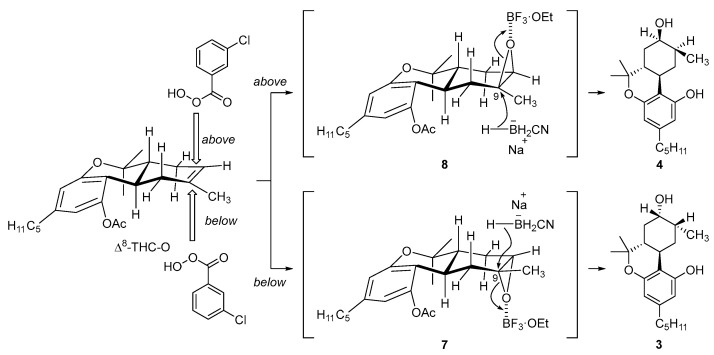
Reaction mechanism of epoxidation to produce compounds **3** and **4**.

The proposed mechanism for the formation of compounds **3** and **4** is shown in Scheme 4. Following the well-known epoxidation reaction using *m*CPBA, the epoxide is activated by BF_3_⋅OEt_2_, after which a hydride derived from NaBH_3_CN attacks 9C to produce compound **3** or **4**.

## 3. Materials and Methods

### 3.1. Materials

All the reagents were purchased from commercial suppliers and used as received. Δ^8^-THC ([α]_D_^23^ –79 (*c* 0.63, CHCl_3_)) was prepared using a previously reported method [14]. The NMR data for the Δ^8^-THC prepared in this manner matched those previously reported [20]. Δ^8^-THC acetate ([α]_D_^23^ –160 (*c* 0.72, CHCl_3_)) was prepared using a previously reported method [18]. The NMR data for the Δ^8^-THC acetate prepared in this manner matched those previously reported [18,21]. Δ^8^-THC and Δ^8^-THC acetate were used as synthesized neutral forms.

### 3.2. Analysis Information

Reaction mixtures were magnetically stirred and monitored by thin-layer chromatography (TLC) using pre-coated silica-gel plates. Column chromatography was performed using silica gel (60 μm). ^1^H (400 MHz) and ^13^C (100 MHz) NMR spectra were recorded on a JNM-ECZ400S NMR spectrometer (JEOL Ltd., Tokyo, Japan) at 296 K unless otherwise stated. Chemical shifts are given in parts per million (ppm) downfield from that of tetramethylsilane as an internal standard (0.00 ppm), and coupling constants (*J*) are reported in Hz. Splitting patterns are abbreviated as follows: singlet (s), doublet (d), triplet (t), multiplet (m), and broad (br). High-resolution mass spectra (HRMS) were recorded on an X500R QTOF electrospray ionization time-of-flight mass spectrometer (AB Sciex LLC, Marlborough, MA, USA). IR spectra were recorded on a Spectrum 100 FT-IR spectrometer equipped with an ATR (diamond) accessory (PerkinElmer, Inc., Waltham, MA, USA). Optical rotations were recorded on a P-1030 Polarimeter (JASCO Corporation, Tokyo, Japan).

### 3.3. Synthesis of ***1*** and ***2***

BH_3_·THF (1.0 M in THF, 0.40 mL, 0.40 mmol) was added to a stirred solution of Δ^8^-THC (63 mg, 0.20 mmol) in THF (2.0 mL, 0.1 M) at 23 °C under an argon atmosphere. The mixture was stirred at 23 °C for 22 h, then water (2.0 mL), 2 N NaOH aq. (2.0 mL) and 30% H_2_O_2_ aq. (2.0 mL) were added. After the mixture was stirred for 15 min, the reaction was quenched with 10% aqueous Na_2_S_2_O_3_, and the product was extracted with ethyl acetate (3×). The combined organic extracts were washed with brine, dried over Na_2_SO_4_, and concentrated under vacuum. The residue was purified using column chromatography (silica gel, hexane/ethyl acetate = 2/1) to obtain **1** (16.6 mg, 26%) and **2** (21.4 mg, 34%) as colorless oils.

**1**: *R*_f_ 0.37 (hexane/ethyl acetate = 2/1); [α]_D_^23^ –50 (*c* 0.49, CHCl_3_); ^1^H NMR (400 MHz, CDCl_3_) *δ* 6.25 (d, 1H, *J* = 1.6 Hz), 6.07 (d, 1H, *J* = 1.6 Hz), 4.80 (brs, 1H, OH), 3.89 (q, 1H, *J* = 2.8 Hz), 2.80 (td, 1H, *J* = 2.4, 13.6 Hz), 2.66 (dt, 1H, *J* = 2.8, 11.6 Hz), 2.48–2.36 (m, 2H), 2.04–1.95 (m, 2H), 1.77 (dtd, *J* = 1.6, 3.2, 13.6 Hz, 1H), 1.68–1.44 (m, 5H), 1.35 (s, 3H), 1.34–1.24 (m, 4H), 1.14 (d, 3H, *J* = 7.6 Hz), 1.09 (s, 3H), 0.88 (t, 3H, *J* = 7.2 Hz); ^13^C NMR (100 MHz, CDCl_3_) *δ* 159.2, 154.6, 142.6, 110.0, 109.8, 107.5, 76.5, 71.5, 42.3, 35.7, 35.4, 31.5, 30.60, 30.55, 30.5, 29.1, 27.4, 22.5, 19.0, 17.6, 14.0; IR (ATR) 3343, 2928, 1578, 1425, 1048 cm^−1^; HRMS (ESI) *m*/*z* calcd. for C_21_H_31_O_3_ ([M − H]^−^): 331.2279, found: 331.2278.

**2**: *R*_f_ 0.29 (hexane/ethyl acetate = 2/1); [α]_D_^23^ –69 (*c* 0.42, CHCl_3_); ^1^H NMR (400 MHz, CDCl_3_) *δ* 6.24 (d, 1H, *J* = 1.2 Hz), 6.08 (d, 1H, *J* = 1.2 Hz), 5.06 (brs, 1H, OH), 3.33 (dt, 1H, *J* = 4.4, 10.4 Hz), 3.12 (td, 1H, *J* = 3.2, 13.2 Hz), 2.48 (dt, 1H, *J* = 2.8, 11.2 Hz), 2.44–2.40 (m, 2H), 2.12 (ddd, 1H, *J* = 2.4, 4.8, 12.0 Hz), 1.68 (brs, 1H, OH), 1.61–1.51 (m, 4H), 1.38 (s, 3H), 1.35–1.25 (m, 4H), 1.20–1.10 (m, 1H), 1.09 (s, 3H), 1.08 (d, 3H, *J* = 7.2 Hz), 0.88 (t, 3H, *J* = 7.2 Hz), 0.93–0.84 (m, 1H); ^13^C NMR (100 MHz, CDCl_3_) *δ* 154.8, 154.7, 142.8, 109.9, 109.3, 107.6, 76.8, 76.6, 47.5, 40.3, 36.9, 36.6, 35.4, 35.0, 31.5, 30.6, 27.7, 22.5, 19.0, 18.4, 14.0; IR (ATR) 3340, 2925, 1578, 1425, 1027 cm^−1^; HRMS (ESI) *m*/*z* calcd. for C_21_H_31_O_3_ ([M − H]^−^): 331.2279, found: 331.2281.

### 3.4. Synthesis of ***5*** and ***6***

65% *m*CPBA (62 mg, 0.23 mmol) was added to a stirred solution of Δ^8^-THC acetate (92 mg, 0.26 mmol) in CH_2_Cl_2_ (10 mL, 0.026 M) at 0 °C under an argon atmosphere. After the mixture was stirred at 0 °C for 4 h, the reaction was quenched with 10% aqueous Na_2_S_2_O_3_, and the product was extracted with CH_2_Cl_2_ (3×). The combined organic extracts were washed with brine, dried over Na_2_SO_4_, and concentrated under vacuum. The residue was purified using column chromatography (silica gel, hexane/ethyl acetate = 4/1) and PTLC (hexane/ethyl acetate = 4/1) to obtain **5** (36.3 mg, 38%) and **6** (19.0 mg, 20%) as colorless oils.

**5**: *R*_f_ 0.34 (hexane/ethyl acetate = 4/1); ^1^H NMR (400 MHz, CDCl_3_) *δ* 6.52 (d, 1H, *J* = 1.6 Hz), 6.40 (d, 1H, *J* = 1.6 Hz), 3.11 (d, 1H, *J* = 5.6 Hz), 2.85 (dd, 1H, *J* = 3.6, 14.4 Hz), 2.56–2.43 (m, 3H), 2.31 (s, 3H), 2.18–2.06 (m, 1H), 1.63–1.53 (m, 4H), 1.49 (dd, 1H, *J* = 11.2, 14.4 Hz), 1.35 (s, 3H), 1.34 (s, 3H), 1.34–1.25 (m, 4H), 1.03 (s, 3H), 0.88 (t, 3H, *J* = 6.8 Hz); ^13^C NMR (100 MHz, CDCl_3_) *δ* 169.0, 154.3, 149.4, 142.7, 115.1, 115.0, 114.5, 76.5, 59.0, 58.5, 44.6, 35.8, 35.2, 31.4, 30.3, 28.1, 27.1, 26.5, 22.9, 22.4, 21.1, 18.2, 13.9; IR (ATR) 2928, 1767, 1371, 1204, 1035 cm^−1^; HRMS (ESI) *m*/*z* calcd. for C_23_H_33_O_4_ ([M + H]^+^): 373.2373, found: 373.2374.

**6**: *R*_f_ 0.31 (hexane/ethyl acetate = 4/1); ^1^H NMR (400 MHz, CDCl_3_) *δ* 6.54 (d, 1H, *J* = 1.6 Hz), 6.39 (d, 1H, *J* = 1.6 Hz), 3.09–3.07 (m, 1H), 2.68 (dd, 1H, *J* = 5.2, 15.2 Hz), 2.50–2.47 (m, 2H), 2.31 (s, 3H), 2.32–2.24 (m, 2H), 1.73–1.63 (m, 2H), 1.61–1.45 (m, 3H), 1.38 (s, 3H), 1.37 (s, 3H), 1.35–1.25 (m, 4H), 1.05 (s, 3H), 0.88 (t, 3H, *J* = 7.2 Hz); ^13^C NMR (100 MHz, CDCl_3_) *δ* 168.8, 154.3, 149.4, 143.0, 115.4, 115.3, 114.3, 59.3, 57.6, 39.3, 35.3, 34.8, 31.5, 31.2, 30.4, 28.3, 27.1, 24.9, 22.5, 21.2, 18.6, 14.0, one signal was overlapped with solvent; IR (ATR) 2928, 1765, 1370, 1193, 1031 cm^−1^; HRMS (ESI) *m*/*z* calcd. for C_23_H_33_O_4_ ([M + H]^+^): 373.2373, found: 373.2373.

### 3.5. Synthesis of ***7***

NaBH_3_CN (39 mg, 0.62 mmol) and BF_3_·OEt_2_ (0.10 mL, 0.80 mmol) were added to a stirred solution of **5** (115 mg, 0.309 mmol) in THF (10 mL, 0.03 M) at 0 °C under an argon atmosphere. After the mixture was stirred at 23 °C for 1 h, the reaction was quenched with water and the product extracted with ethyl acetate (3×). The combined organic extracts were washed with brine, dried with Na_2_SO_4_, and concentrated under vacuum. The residue was purified using column chromatography (silica gel, hexane/ethyl acetate = 2/1) to obtain **7** (16.0 mg, 14%) as a colorless oil.

**7**: *R*_f_ 0.43 (hexane/ethyl acetate = 2/1); ^1^H NMR (400 MHz, CDCl_3_) *δ* 6.55 (d, 1H, *J* = 1.6 Hz), 6.38 (d, 1H, *J* = 1.6 Hz), 3.91 (td, 1H, *J* = 4.8, 11.6 Hz), 2.55–2.46 (m, 4H),2.31 (s, 3H), 2.28–2.20 (m, 1H), 1.89–1.81 (m, 1H), 1.61–1.48 (m, 4H), 1.42–1.34 (m, 1H), 1.38 (s, 3H), 1.33–1.23 (m, 5H), 1.10 (s, 3H), 1.06 (d, 3H, *J* = 6.8 Hz), 0.88 (t, 3H, *J* = 6.8 Hz); ^13^C NMR (100 MHz, CDCl_3_) *δ* 169.2, 154.8, 149.5, 142.7, 115.6, 115.2, 114.2, 77.2, 69.8, 48.9, 43.0, 39.3, 35.3, 31.7, 31.5, 31.0, 30.4, 27.5, 23.9, 22.5, 21.2, 19.1, 14.0; IR (ATR) 3531, 2930, 1742, 1370, 1204 cm^−1^; HRMS (ESI) *m*/*z* calcd. for C_23_H_35_O_4_ ([M + H]^+^): 375.2530, found: 375.2525.

### 3.6. Synthesis of ***8***

Compound **8** was prepared following the standard procedure described for **7**.

**8**: 17% yield; colorless oil; *R*_f_ 0.49 (hexane/ethyl acetate = 2/1); ^1^H NMR (400 MHz, CDCl_3_) *δ* 6.54 (d, 1H, *J* = 1.6 Hz), 6.37 (d, 1H, *J* = 1.6 Hz), 3.96–3.91 (m, 1H), 2.51–2.46 (m, 2H), 2.40–2.27 (m, 2H), 2.28 (s, 3H), 1.99–1.90 (m, 2H), 1.78–1.69 (m, 1H), 1.63–1.53 (m, 2H), 1.41–1.24 (m, 7H), 1.37 (s, 3H), 1.07 (s, 3H), 1.02 (d, 3H, *J* = 6.8 Hz), 0.88 (t, 3H, *J* = 6.8 Hz); ^13^C NMR (100 MHz, CDCl_3_) *δ* 169.0, 154.6, 149.6, 142.8, 115.3, 115.2, 114.1, 76.9, 70.0, 41.3, 36.8, 35.7, 35.33, 35.30, 32.3, 31.5, 30.4, 27.4, 22.5, 21.2, 19.0, 18.2, 14.0; IR (ATR) 3529, 2929, 1765, 1368, 1206 cm^−1^; HRMS (ESI) *m*/*z* calcd. for C_23_H_35_O_4_ ([M + H]^+^): 375.2530, found: 375.2522.

### 3.7. Synthesis of ***3***

A solution of NaOH (14 mg, 0.35 mmol) in MeOH (4 mL, 0.01 M) was added to **7** (16 mg, 0.043 mmol) at 23 °C under an argon atmosphere. The mixture was stirred at 23 °C for 4 h. After it was concentrated under vacuum, the residue was purified by column chromatography (silica gel, hexane/ethyl acetate = 2/1) to obtain **3** (11.8 mg, 83%) as a colorless oil.

**3**: *R*_f_ 0.28 (hexane/ethyl acetate = 2/1); [α]_D_^23^ –52 (*c* 0.66, CHCl_3_); ^1^H NMR (400 MHz, CDCl_3_) *δ* 6.25 (d, 1H, *J* = 1.6 Hz), 6.07 (d, 1H, *J* = 1.6 Hz), 4.83 (brs, 1H, OH), 3.93 (td, 1H, *J* = 4.8, 11.6 Hz), 3.06 (td, 1H, *J* = 2.8, 13.6 Hz), 2.64 (dt, 1H, *J* = 2.8, 11.6 Hz), 2.44–2.40 (m, 2H), 2.29–2.20 (m, 1H), 1.88–1.82 (m, 1H), 1.63 (brs, 1H, OH), 1.59–1.49 (m, 3H), 1.38 (s, 3H), 1.34–1.25 (m, 6H), 1.12 (d, 3H, *J* = 8.0 Hz), 1.11 (s, 3H), 0.88 (t, 3H, *J* = 7.2 Hz); ^13^C NMR (100 MHz, CDCl_3_) *δ* 155.0, 154.6, 142.7, 109.9, 109.4, 107.6, 76.6, 73.1, 47.7, 35.4, 34.5, 34.4, 31.5, 31.1, 30.6, 28.4, 27.5, 22.5, 19.2, 14.0, 11.8; IR (ATR) 3444, 2934, 1571, 1408, 1012 cm^−1^; HRMS (ESI) *m*/*z* calcd. for C_21_H_31_O_3_ ([M − H]^−^): 331.2279, found: 331.2276.

### 3.8. Synthesis of ***4***

Compound **4** was prepared following the standard procedure described for **3**.

**4**: 62% yield; colorless oil; *R*_f_ 0.48 (hexane/ethyl acetate = 2/1); [α]_D_^23^ –27 (*c* 0.33, CHCl_3_); ^1^H NMR (400 MHz, CDCl_3_) *δ* 6.25 (d, 1H, *J* = 1.6 Hz), 6.08 (d, 1H, *J* = 1.6 Hz), 4.72 (brs, 1H, OH), 3.98–3.94 (m, 1H), 2.83 (td, 1H, *J* = 3.2, 13.2 Hz), 2.50–2.40 (m, 3H), 2.01–1.90 (m, 2H), 1.86–1.76 (m, 1H), 1.59–1.52 (m, 3H), 1.41–1.25 (m, 5H), 1.37 (s, 3H), 1.23–1.13 (m, 1H), 1.08 (s, 3H), 1.02 (d, 3H, *J* = 6.8 Hz), 0.88 (t, 3H, *J* = 6.8 Hz); ^13^C NMR (100 MHz, CDCl_3_) *δ* 154.9, 154.6, 142.7, 110.1, 109.6, 107.5, 77.2, 70.4, 41.5, 36.6, 35.4, 31.9, 31.5, 30.6, 27.6, 22.5, 18.9, 18.1, 14.0, several signals were overlapped; IR (ATR) 3338, 2927, 1576, 1426, 1042 cm^−1^; HRMS (ESI) *m*/*z* calcd. for C_21_H_31_O_3_ ([M − H]^−^): 331.2279, found: 331.2276.

## 4. Conclusions

Using two simple strategies, we achieved the syntheses of (8*R*)OH-(6a*R*,9*R*,10a*R*)-HHC (**1**), (8*S*)OH-(6a*R*,9*S*,10a*R*)-HHC (**2**), (8*S*)OH-(6a*R*,9*R*,10a*R*)-HHC (**3**), and (8*R*)OH-(6a*R*,9*S*,10a*R*)-HHC (**4**), which are the metabolites of (9*R*)-HHC and (9*S*)-HHC. For the synthesis of (8*R*)OH-(6a*R*,9*R*,10a*R*)-HHC (**1**) and (8*S*)OH-(6a*R*,9*S*,10a*R*)-HHC (**2**), we utilized a conventional hydroboration–oxidation reaction, which provided the desired products (8*R*)OH-(6a*R*,9*R*,10a*R*)-HHC (**1**) and (8*S*)OH-(6a*R*,9*S*,10a*R*)-HHC (**2**). In this reaction, the yields of **1** and **2** varied depending on the reaction conditions. However, unexpectedly, compound **3** was always obtained, albeit in small amounts. Although the reason for obtaining compound **3** is unknown, it is likely that the hydroboration reaction proceeded via a different mechanism. Regarding the synthesis of (8*S*)OH-(6a*R*,9*R*,10a*R*)-HHC (**3**) and (8*R*)OH-(6a*R*,9*S*,10a*R*)-HHC (**4**), regioselective reduction was attempted; however, only moderate selectivity was achieved. Nevertheless, all four diastereomers were successfully isolated, and their absolute configurations were fully determined. Further synthetic refinements and a more detailed mechanistic investigation into the formation of compounds **1**–**4**, supported by DFT calculations, will be reported in future work.

## Figures and Tables

**Table 1:** Optimization of the reaction conditions.

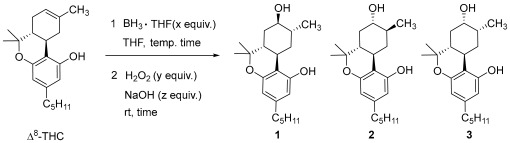								
	Reaction 1			Reaction 2				
Entry	x (Equiv.)	Temp. (°C)	Time (h)	Y (Equiv.)	Z (Equiv.)	Time (h)	1 (%)	2 + 3 (%) (Ratio)
1	2	−20	2	10	10	1	24	20 (5:1)
2	2	−10	21	10	10	30	26	34 (4:1)
3	2	0	2	10	10	15	40	35 (7:1)
4	2	0	2.5	>>10	10	60	14	25 (5:1)
5	2.5	0	3	>>10	>>10	15	26	8 (10:1)
6	2.2	23	4	>>10	>>10	15	15	30 (2:1)

## Data Availability

Data are contained within the article and Supplementary Material.

## Supplementary Materials

The following supporting information can be downloaded at https://www.mdpi.com/article/10.3390/molecules31020289/s1. Figures S1–S6: NMR spectra of compound **1**; Figures S7–S12: NMR spectra of compound **2**; Figures S13–S18: NMR spectra of compound **3**; Figures S19–S24: NMR spectra of compound **4**; Figures S25 and S26: NMR spectra of compound **5**; Figures S27 and S28: NMR spectra of compound **6**; Figures S29 and S30: NMR spectra of compound **7**; Figures S31 and S32: NMR spectra of compound **8**; Figures S33–S38: NMR spectra of Δ^8^-THC; Figures S39–S44: NMR spectra of Δ^8^-THC acetate. Table S1: Assignment of the peaks in the ^1^H and ^13^C NMR spectra of **1**. Table S2: Assignment of the peaks in the ^1^H and ^13^C NMR spectra of **2**. Table S3: Assignment of the peaks in the ^1^H and ^13^C NMR spectra of **3**. Table S4: Assignment of the peaks in the ^1^H and ^13^C NMR spectra of **4**.

## References

[1] United Nations Office on Drugs and Crime (2022). World Drug Report 2022.

[2] Rock E.M., Parker L.A., Murillo-Rodriguez E., Pandi-Perumal S.R., Monti J.M. (2020). Cannabinoids and Neuropsychiatric Disorders.

[3] Teixeira H.M. (2025). Phytocanabinoids and synthetic cannabinoids: From recreational consumption to potential therapeutic use–A review. Front. Toxcol..

[4] Ujváry I. (2024). Hexahydrocannabinol and closely related semi-synthetic cannabinoids: A comprehensive review. Drug Test. Anal..

[5] Hively R.L., Mosher W.A., Hoffmann F.W. (1966). Isolation of *trans*-Δ^6^-Tetrahydrocannabinol from marijuana. J. Am. Chem. Soc..

[6] Docampo-Palacios M.L., Ramirez G.A., Tesfatsion T.T., Okhovat A., Pittiglio M., Ray K.P., Cruces W. (2023). Saturated cannabinoids: Update on synthesis strategies and biological studies of these emerging cannabinoid analogs. Molecules.

[7] Seccamani P., Franco C., Protti S., Porta A., Profumo A., Caprioglio D., Salamone S., Mannucci B., Merli D. (2021). Photochemistry of cannabidiol (CBD) revised. A combined preparative and spectrometric investigation. J. Nat. Prod..

[8] Tanaka R., Kikura-Hanajiri R. (2024). Identification of hexahydrocannabinol (HHC), dihydro-*iso*-tetrahydrocannabinol (dihydro-*iso*-THC) and hexahydrocannabiphorol (HHCP) in electronic cigarette cartridge products. Forensic Toxicol..

[9] Harvey D.J., Brown N.K. (1990). A method based on catalytic hydrogenation for the identification of monohydroxy metabolites of isomeric tetrahydrocannabinols. Rapid Commun. Mass Spectrom..

[10] Di Trana A., Di Giorgi A., Sprega G., Carlier J., Kobidze G., Montanari E., Taoussi O., Bambagiotti G., Fede M.S., Lo Faro A.F. (2024). Disposition of hexahydrocannabinol epimers and their metabolites in biological matrices following a single administration of smoked hexahydrocannabinol: A preliminary study. Pharmaceuticals.

[11] Schirmer W., Auwärter V., Kaudewitz J., Schürch S., Weinmann W. (2023). Identification of human hexahydrocannabinol metabolites in urine. Eur. J. Mass Spectrom. Behav..

[12] Lindbom K., Norman C., Baginski S., Krebs L., Stalberga D., Rautio T., Wu X., Kronstrand R., Gréen H. (2025). Human metabolism of the semi-synthetic cannabinoids hexahydrocannabinol, hexahydrocannabiphorol and their acetates using hepatocytes and urine samples. Drug Test. Anal..

[13] Mechoulam R., Lander N., Varkony T.H., Kimmel I., Becker O., Ben-Zvi Z., Edery H., Porath G. (1980). Stereochemical requirements for cannabinoid activity. J. Med. Chem..

[14] Mechoulam R., Braun P., Gaoni Y. (1972). Syntheses of Δ^1^-tetrahydrocannabinol and related cannabinoids. J. Am. Chem. Soc..

[15] Carey F.A., Sundberg R.J. (2002). Advanced Organic Chemistry, Part B: Reactions and Synthesis.

[16] Hilario-Martinez J.C., Murillo F., Garcia-Mendez J., Dzib E., Sandoval-Ramirez J., Munoz-Hernandez M.A., Bernes S., Kurti L., Duarte F., Merino G. (2020). *trans*-Hydroboration–oxidation products in Δ^5^-steroids via a hydroboration-retro-hydroboration mechanism. Chem. Sci..

[17] Dethe D.H., Erande R.D., Mahapatra S., Das S., Kumar B.V. (2015). Protecting group free enantiospecific total syntheses of structurally diverse natural products of the tetrahydrocannabinoid family. Chem. Commun..

[18] Inayama S., Sawa A., Hosoya E. (1974). The oxidation of Δ^1^- and Δ^6^-tetrahydrocannabinol with selenium dioxide. Chem. Pharm. Bull..

[19] Yamamoto I., Narimatsu S., Watanabe K., Yoshimura H. (1981). Synthesis of 8α, 9α- and 8β, 9β-epoxyhexahydrocannabinols. Chem. Pharm. Bull..

[20] Choi Y.H., Hazekamp A., Peltenburg-Looman A.M.G., Frédérich M., Erkelens C., Lefeber A.W.M., Verpoorte R. (2004). NMR assignments of the major cannabinoids and cannabiflavonoids isolated from flowers of *Cannabis sativa*. Phytochem. Anal..

[21] Binder M., Franke I., Schmidt B., Dietrich W. (1982). ^13^C-NMR. Spectra of natural and semi-synthetic cannabinoids. Helv. Chim. Acta.

