# Supplementary material for: Stereoselective Synthesis and Structural Confirmation of All Four 8-Hydroxyhexahydrocannabinol Stereoisomers
Source: Molecules. 2026 Jan 13;31(2):289. doi: 10.3390/molecules31020289 (PMC12844277; doi:10.3390/molecules31020289)

Supporting Information

Stereoselective synthesis and structural confirmation of all four 8-hydroxyhexahydrocannabinol stereoisomers

Kei Ieuji [1], Kayo Nakamura [1,*] and Hideyo Takahashi [1,*]
[1] Faculty of Pharmaceutical Sciences, Tokyo University of Science, 6-3-1 Niijuku, Katsushika-ku, Tokyo 125-8585, Japan

kayo_nakamura@rs.tus.ac.jp, hide-tak@rs.tus.ac.jp

Contents

1. NMR Spectra of compound **1**........S2
2. NMR Spectra of compound **2**........S9
3. NMR Spectra of compound **3**........S16
4. NMR Spectra of compound **4**........S23
5. NMR Spectra of compound **5**........S30
6. NMR Spectra of compound **6**........S33
7. NMR Spectra of compound **7**........S36
8. NMR Spectra of compound **8**........S39
9. NMR Spectra of Δ^8-THC........S42
10. NMR Spectra of Δ^8-THC acetate........S47

1. NMR Spectra of **1**

Figure S1. ^{1}H NMR (400 MHz, $CDCl_3$, ppm) of **1**

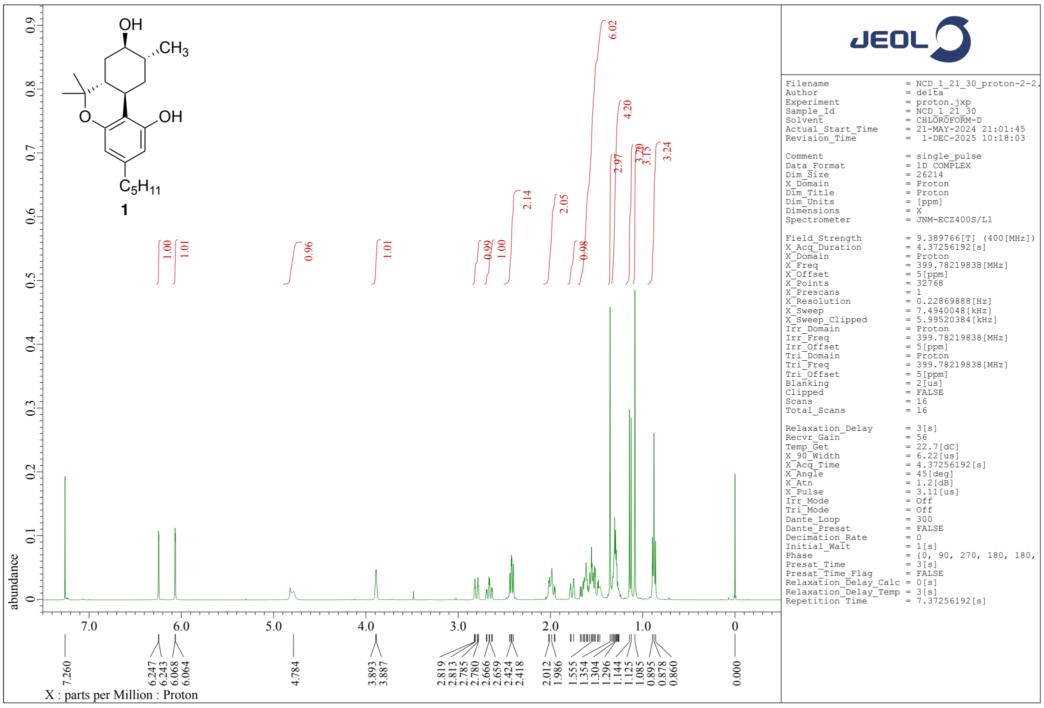

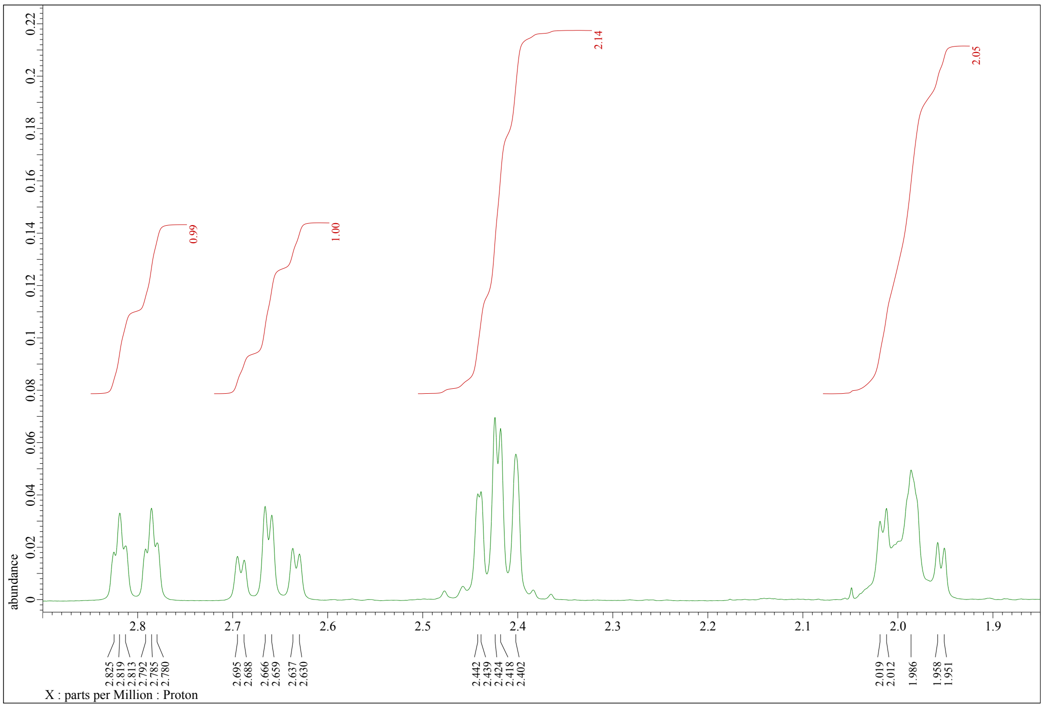

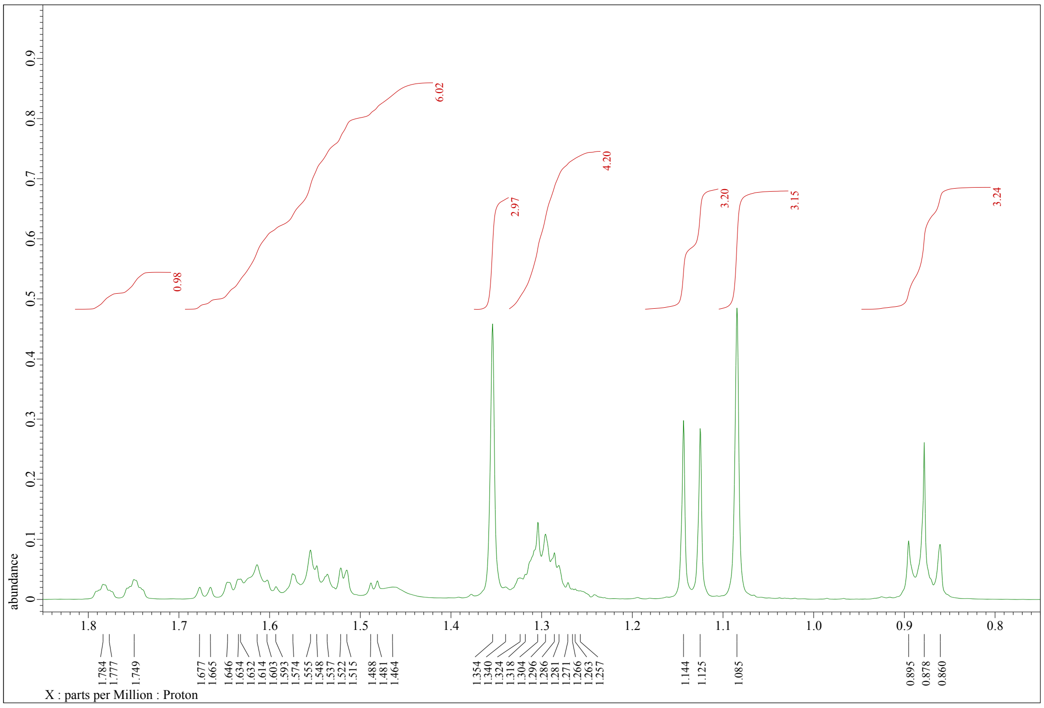

Figure S2. ^{13}C NMR (100 MHz, $CDCl_3$, ppm) of **1**

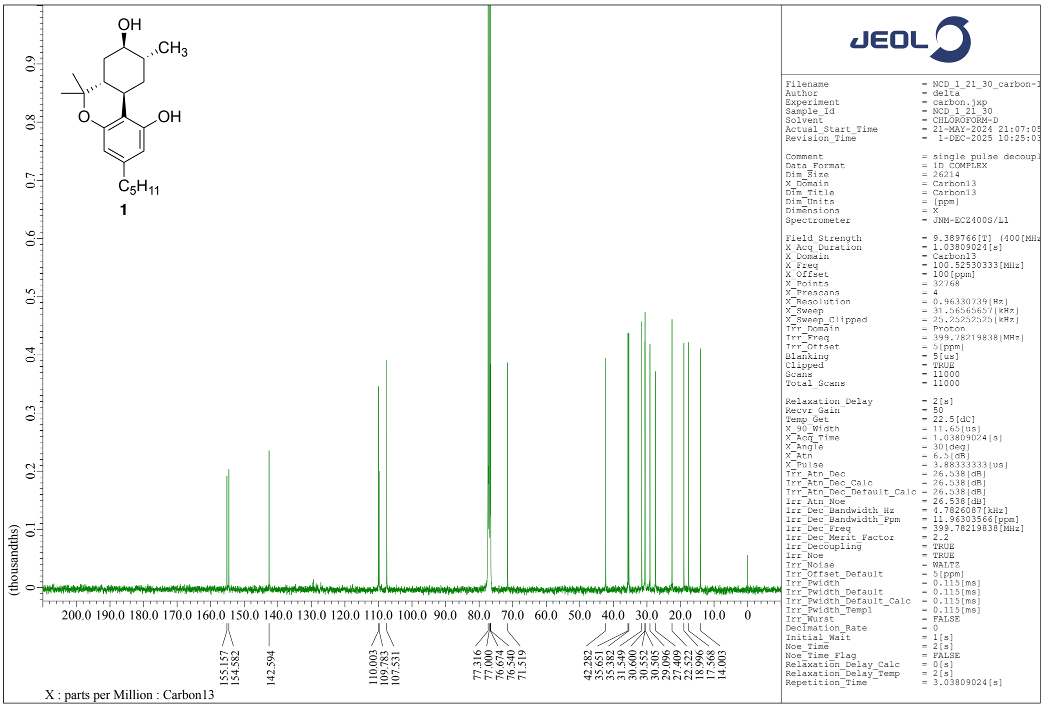

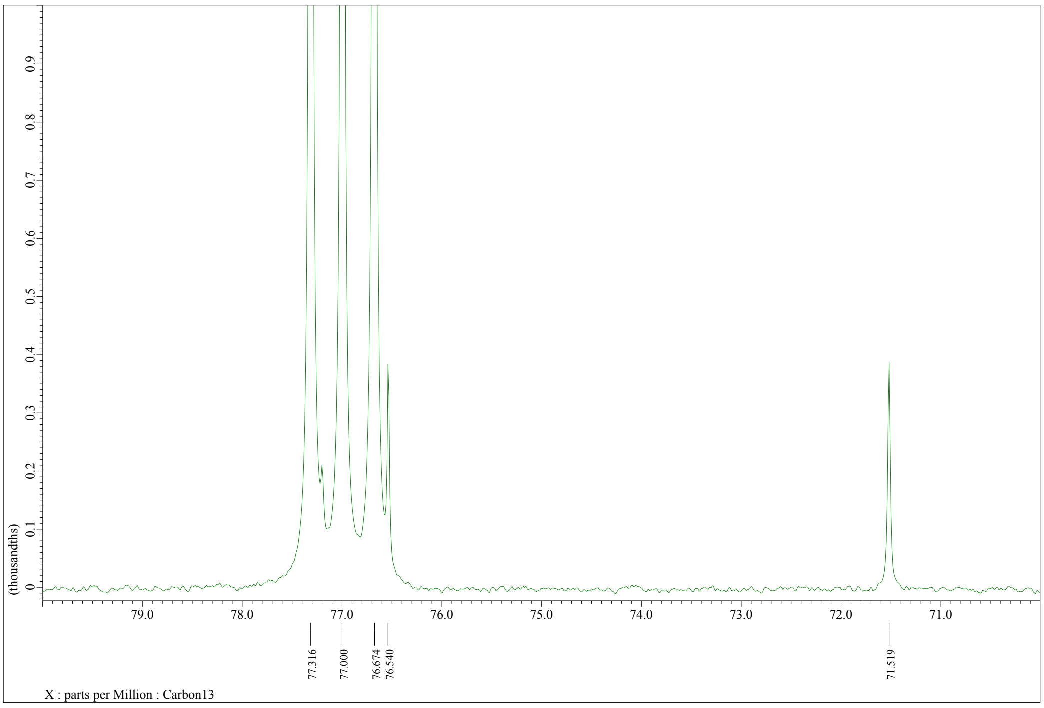
77.316
77.000
76.674
76.540
71.519
(thousandths)
X : parts per Million : Carbon13

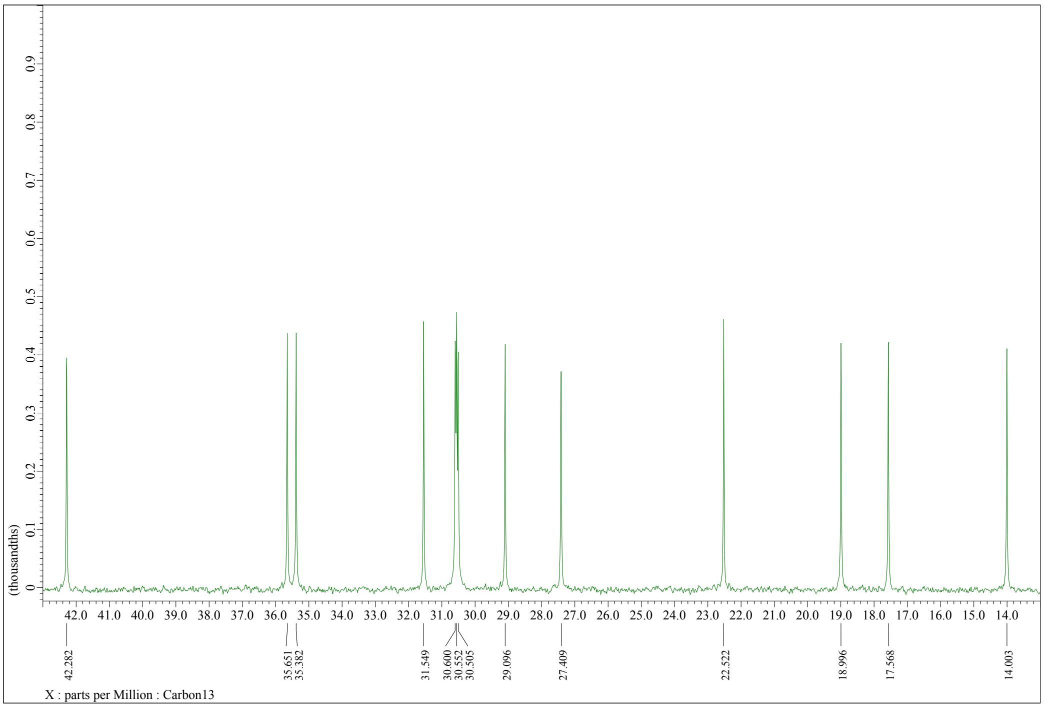
42.282
35.651
35.382
31.549
30.600
30.552
30.505
29.096
27.409
22.522
18.996
17.568
14.003
(thousandths)
X : parts per Million : Carbon13

Figure S3. NOESY spectrum of **1**

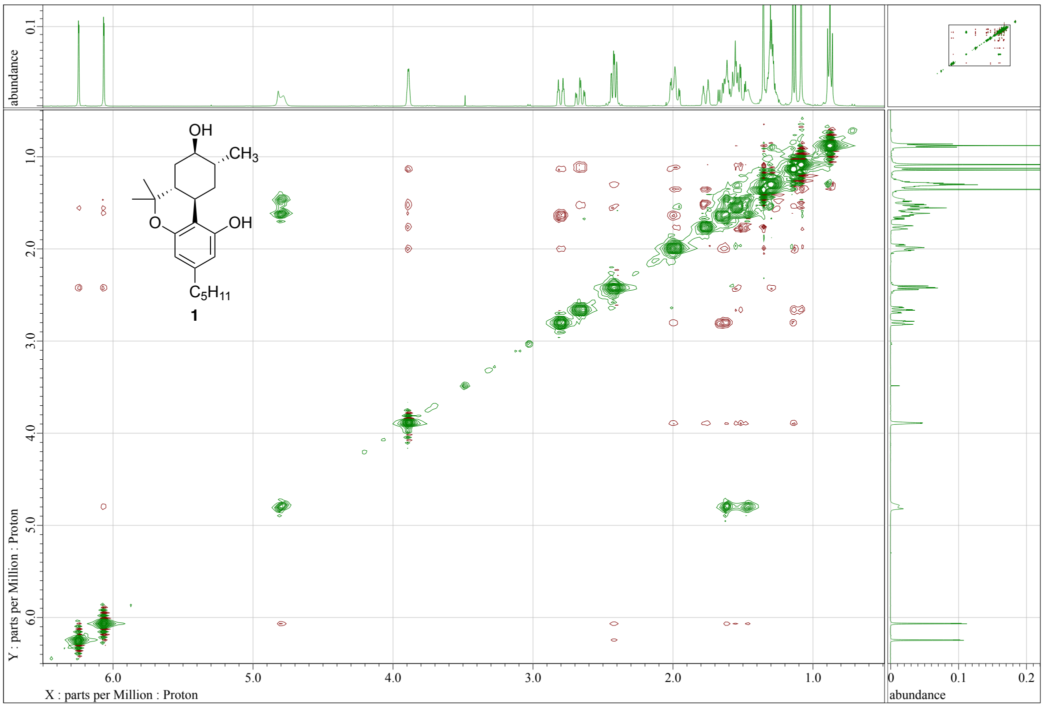

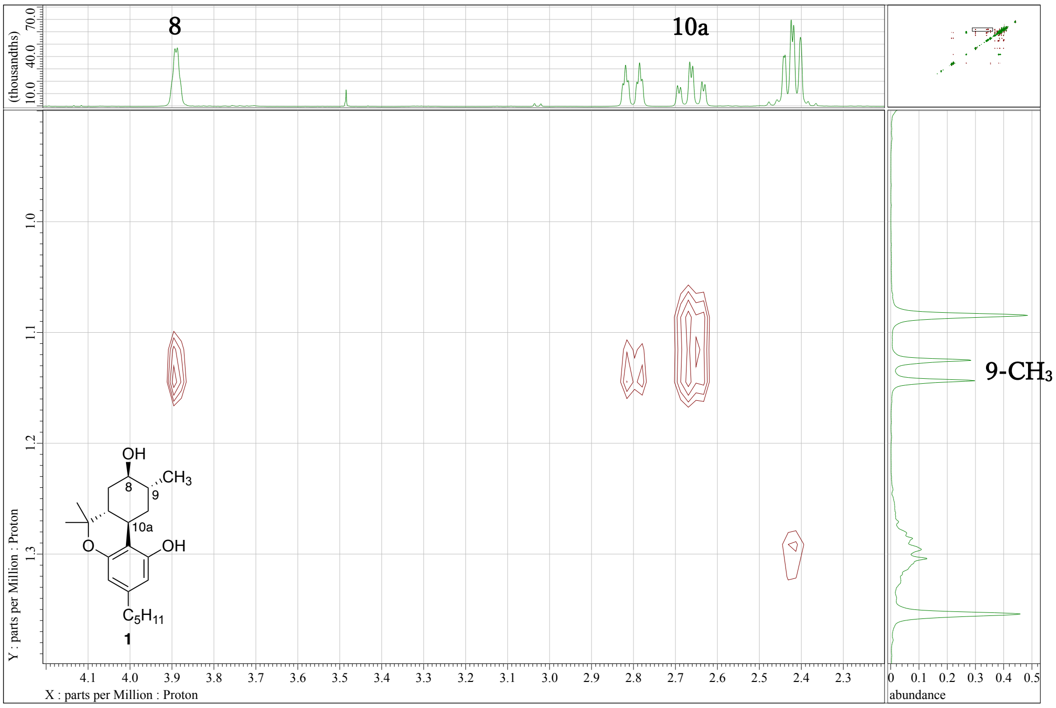

Figure S4. COSY spectrum of **1**

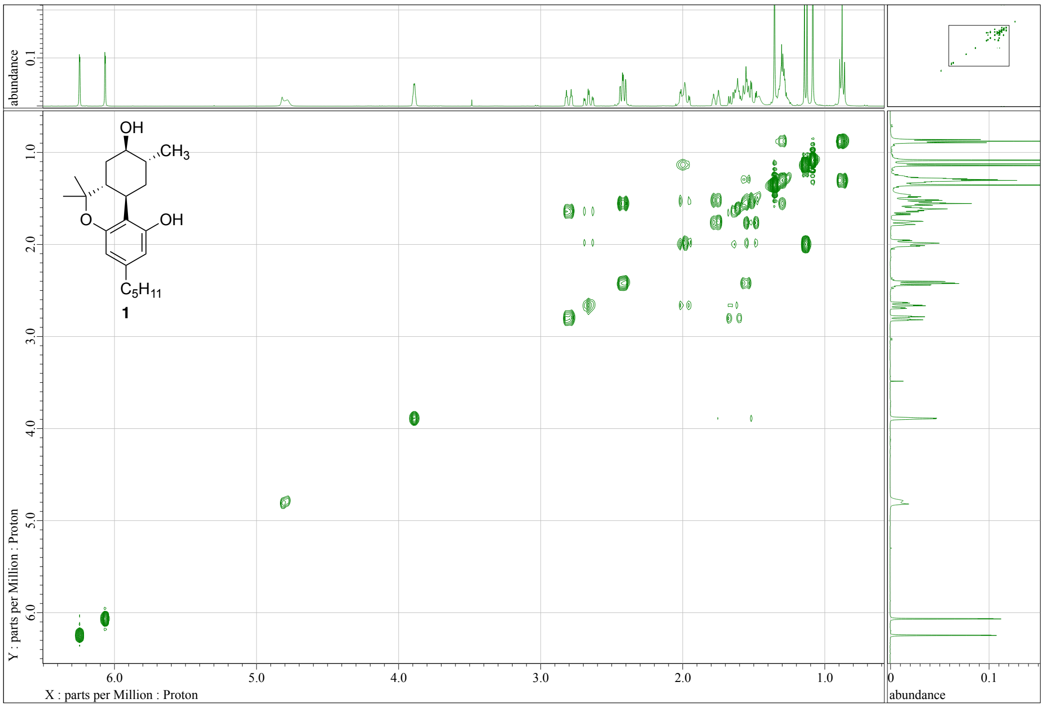

Figure S5. HSQC spectrum of **1**

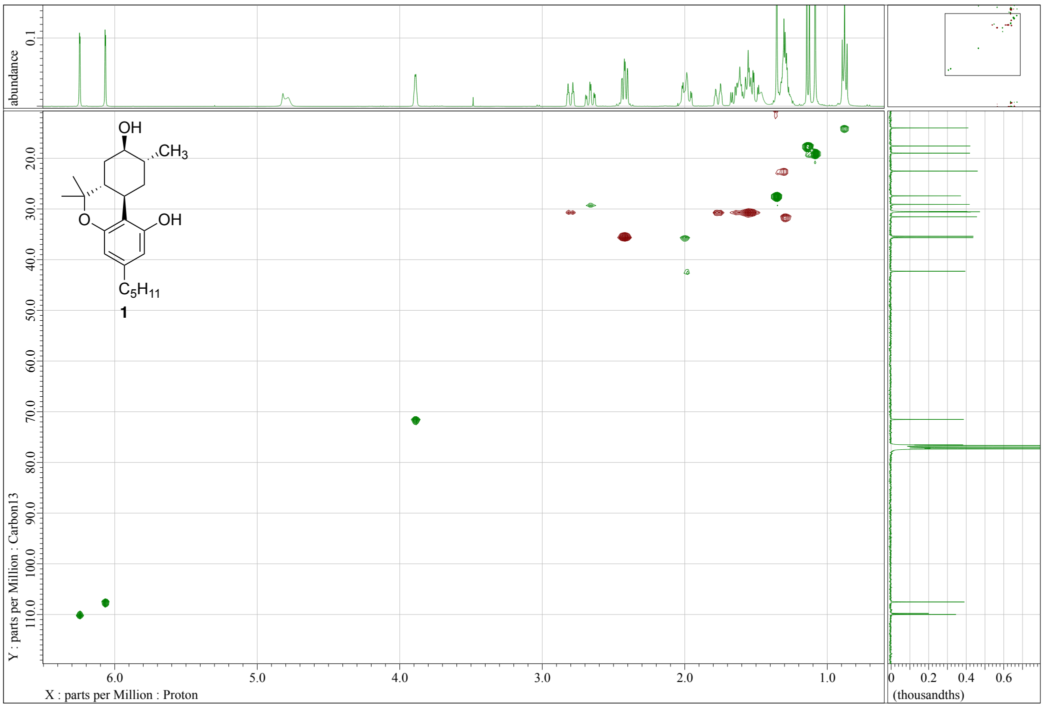

Figure S6. HMBC spectrum of **1**

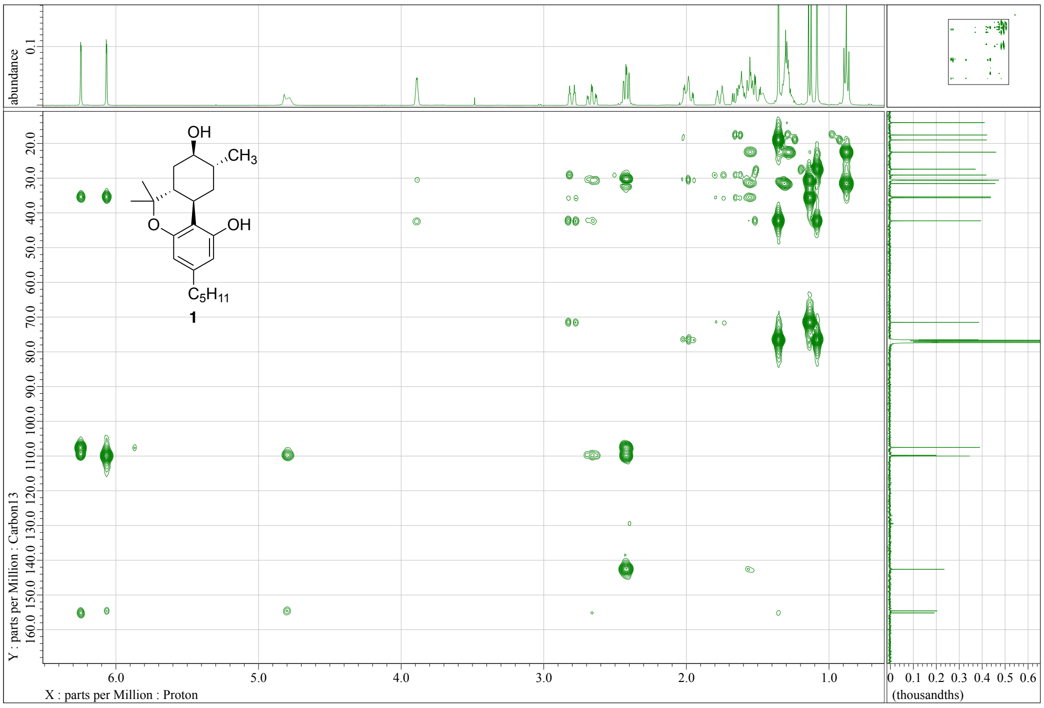

Table S1. Assignment of the peaks in the ^{1}H and ^{13}C NMR spectra of **1**.

	^{1}H	^{13}C		^{1}H	^{13}C
1		154.6	10	2.80 (td, 1H, 10α)	30
				1.68–1.44 (m, 1H, 10β)	
1-OH	4.80 (brs, 1H)		10a	2.66 (dt, 1H)	29.1
2	6.07 (d, 1H)	107.5	10b		109.8
3		142.6	11	1.14 (d, 3H)	17.6
4	6.25 (d, 1H)	110.0	12	1.09 (s, 3H)	19.0
4a		155.2	13	1.35 (s, 3H)	27.4
6		76.5	1’	2.48–2.36 (m, 2H)	35.4
6a	2.04–1.95 (m, 1H)	42.3	2’	1.68–1.44 (m, 2H)	30
7	1.68–1.44 (m, 1H, 7α)	30	3’	1.34–1.24 (m, 2H)	31.5
	1.77 (dtd, 1H, 7β)				
8	3.89 (q, 1H)	71.5	4’	1.34–1.24 (m, 2H)	22.5
8-OH	1.68–1.44 (m, 1H)		5’	0.88 (t, 3H)	14.0
9	2.04–1.95 (m, 1H)	35.7			

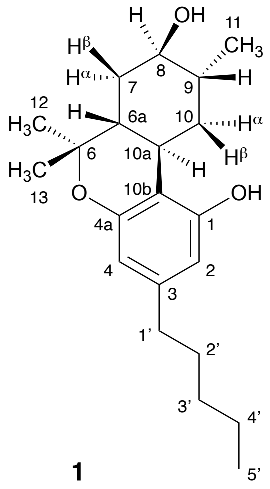

1

2. NMR Spectra of **2**

Figure S7. ^{1}H NMR (400 MHz, $CDCl_3$, ppm) of **2**

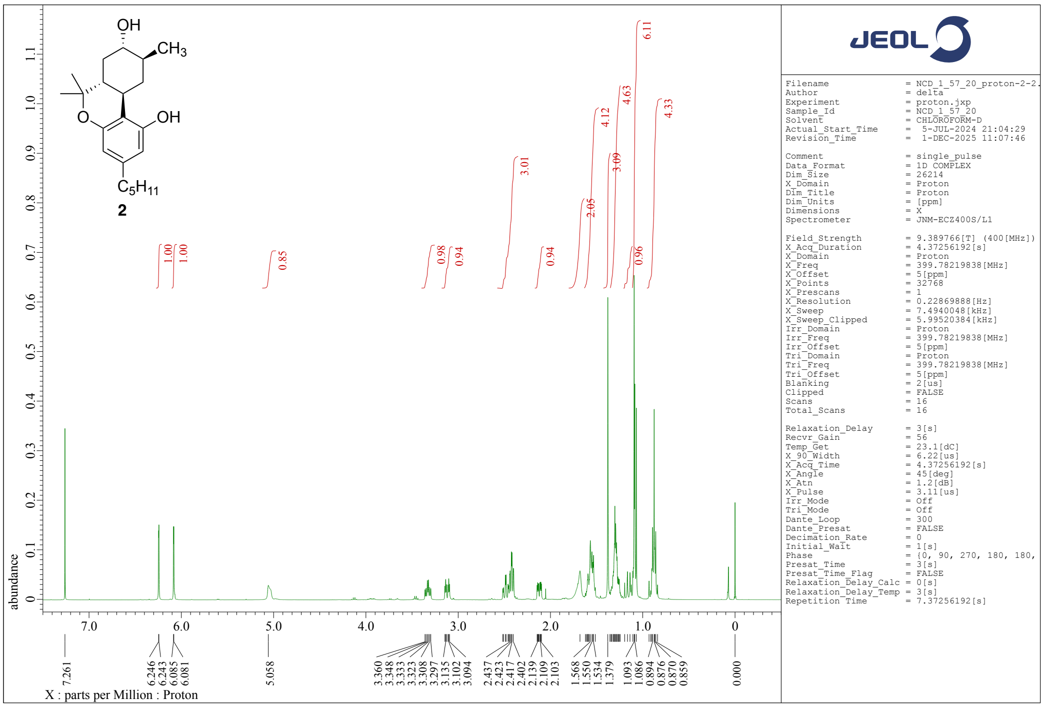

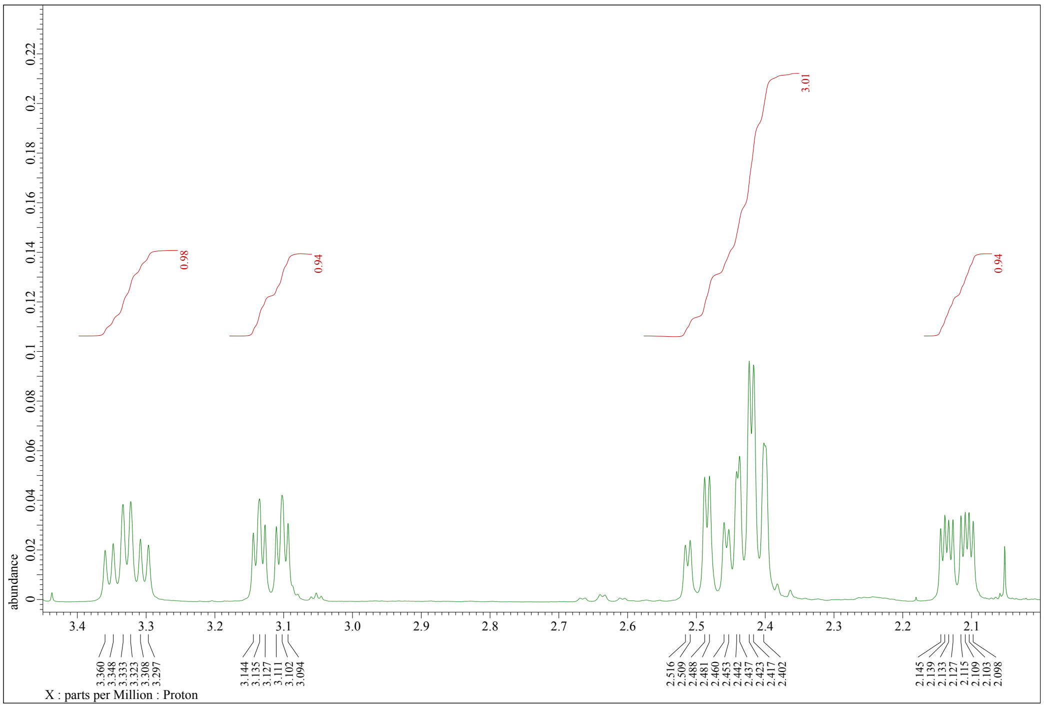

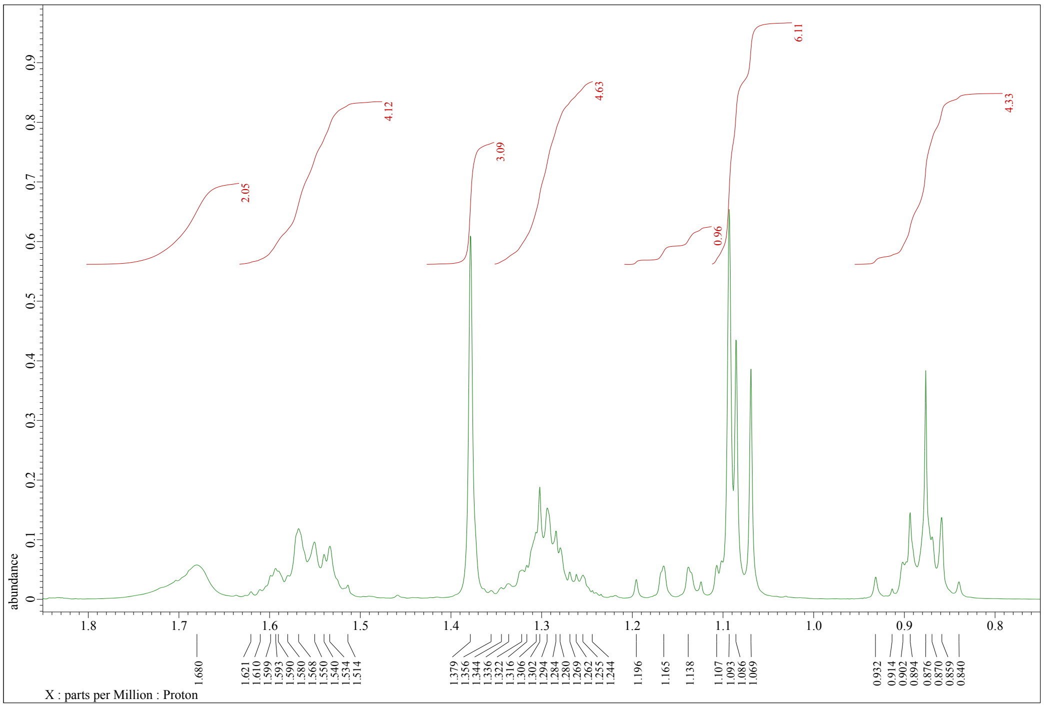

Figure S8. ^{13}C NMR (100 MHz, $CDCl_3$, ppm) of **2**

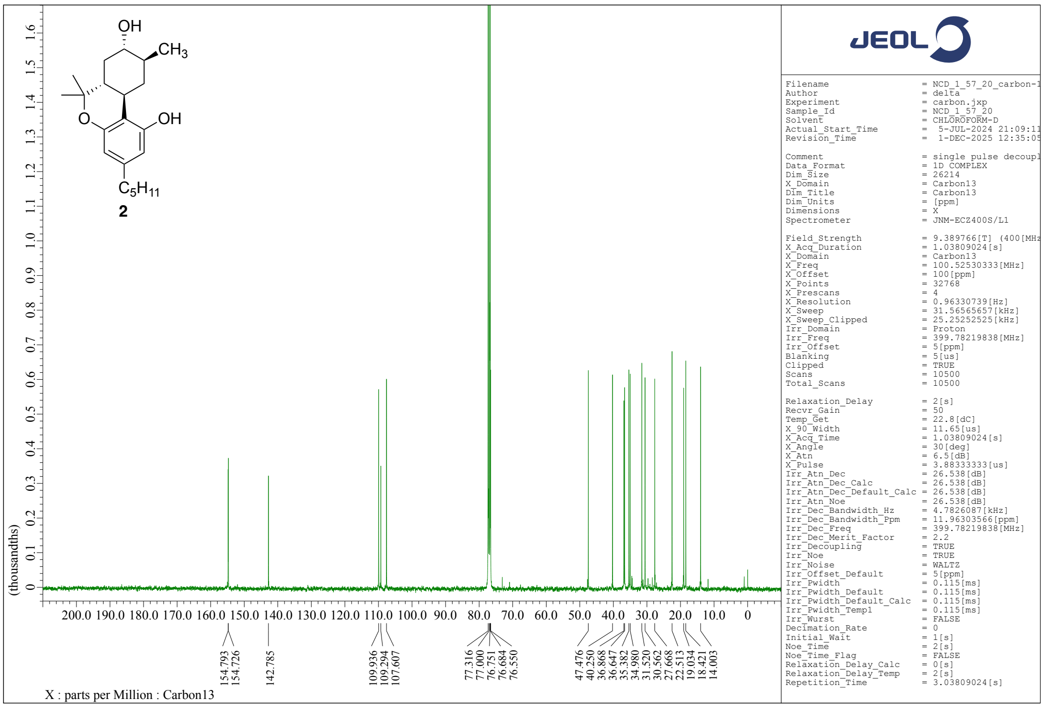

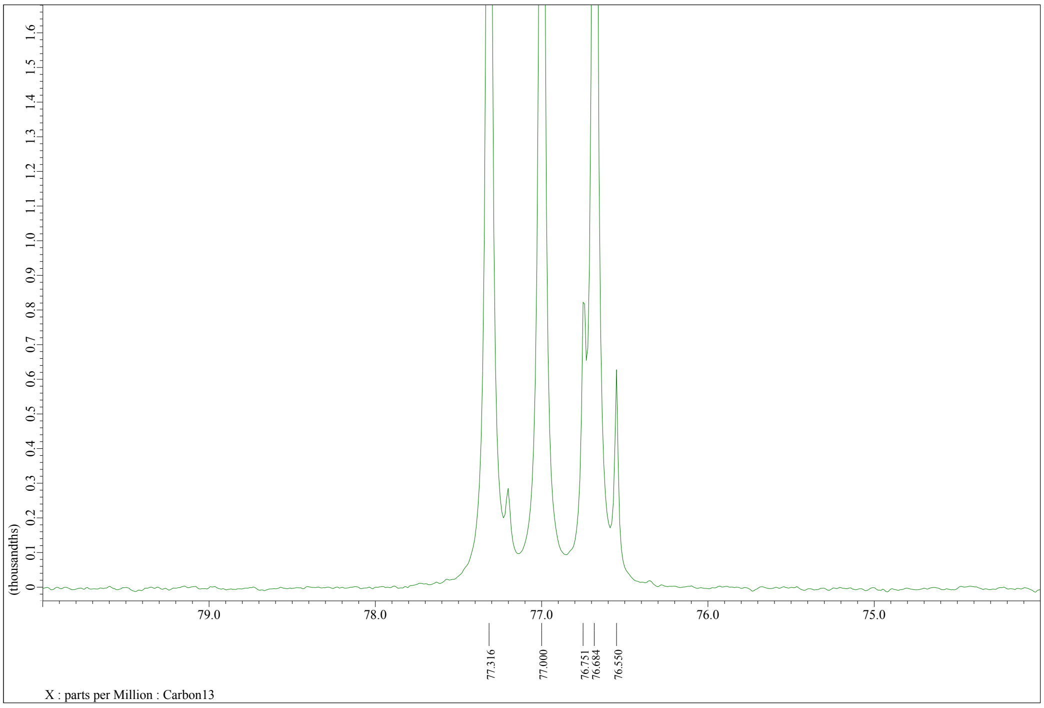
(thousandths)
79.0
78.0
77.0
76.0
75.0
77.316
77.000
76.751
76.684
76.550
X : parts per Million : Carbon13

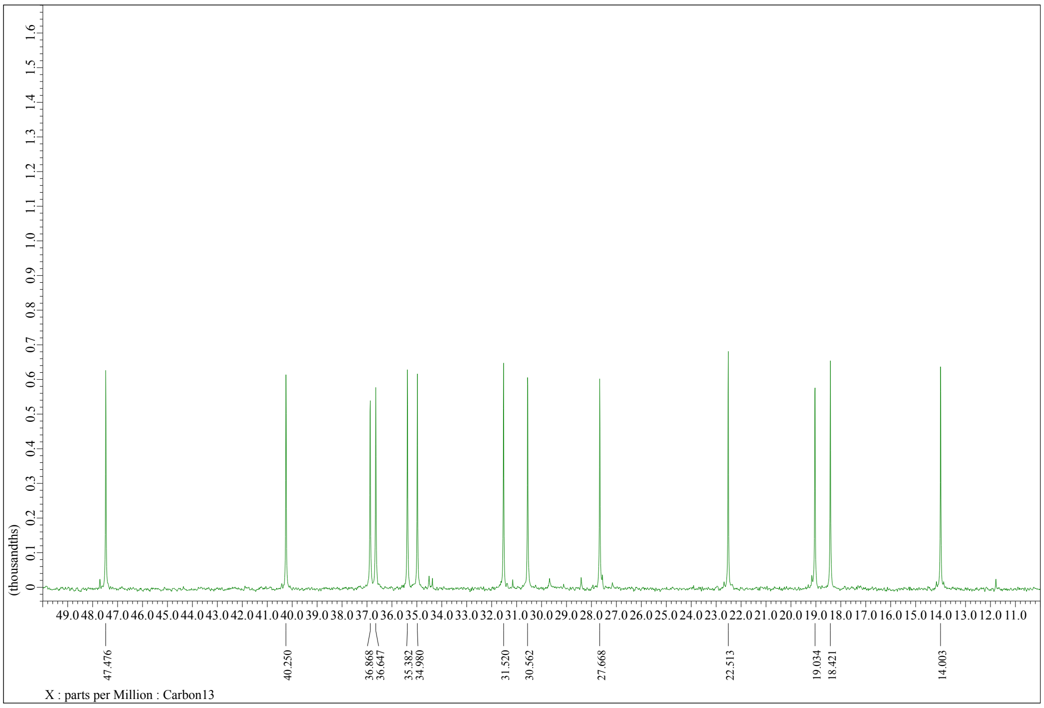
(thousandths)
47.476
40.250
36.868
36.647
35.382
34.980
31.520
30.562
27.668
22.513
19.034
18.421
14.003
X : parts per Million : Carbon13

Figure S9. NOESY spectrum of **2**

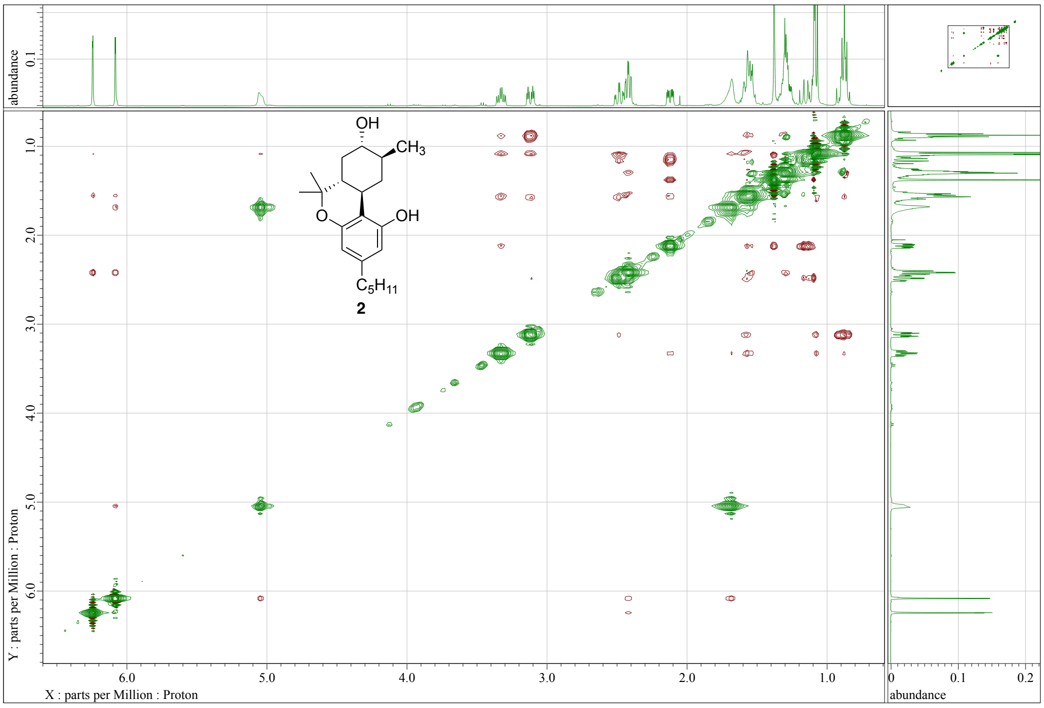

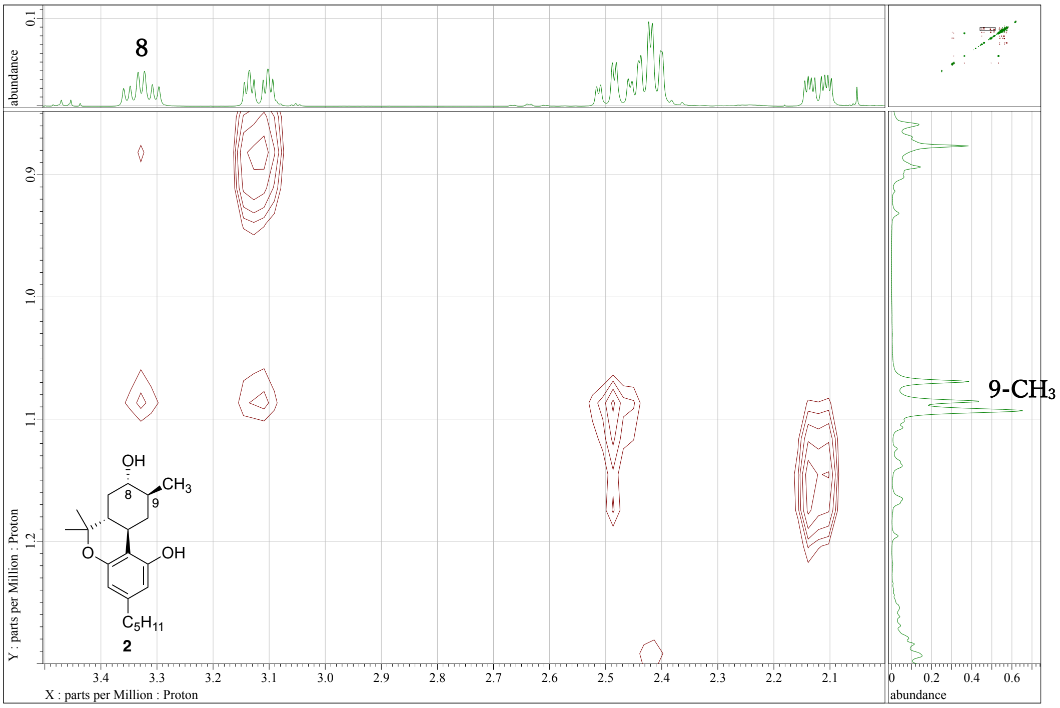

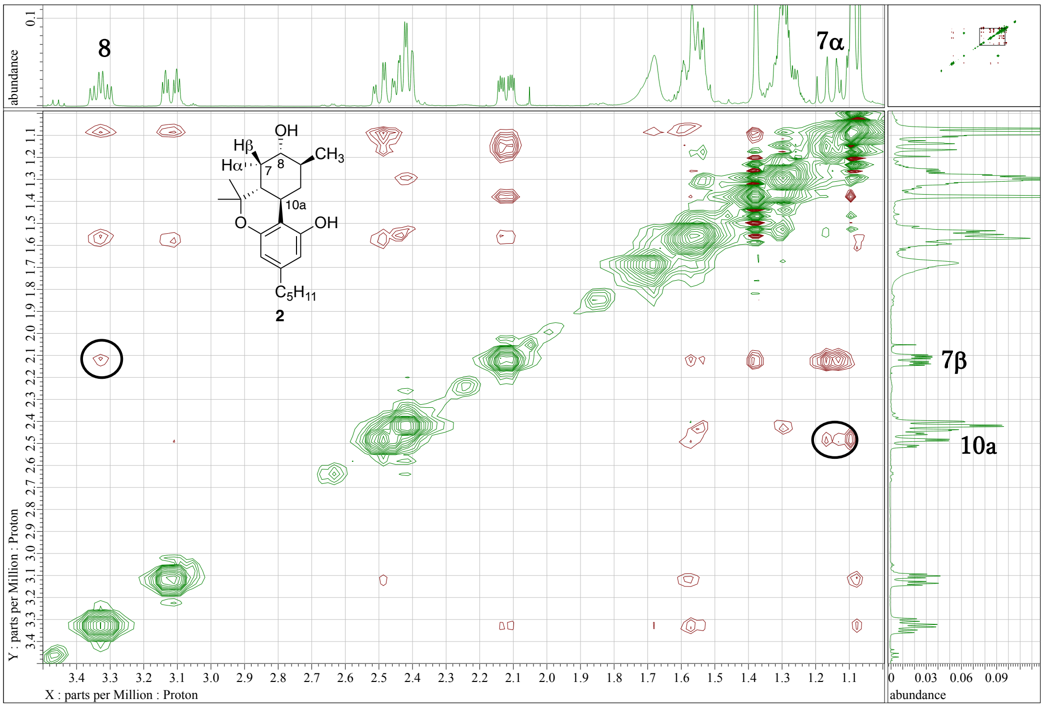

Figure S10. COSY spectrum of **2**

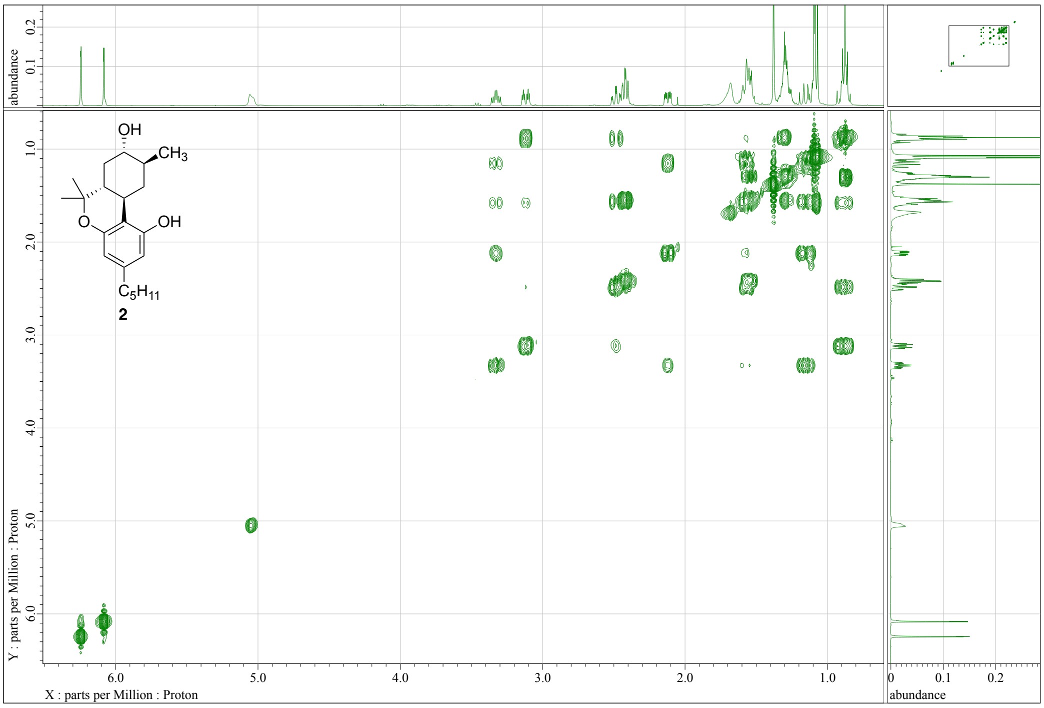

Figure S11. HSQC spectrum of **2**

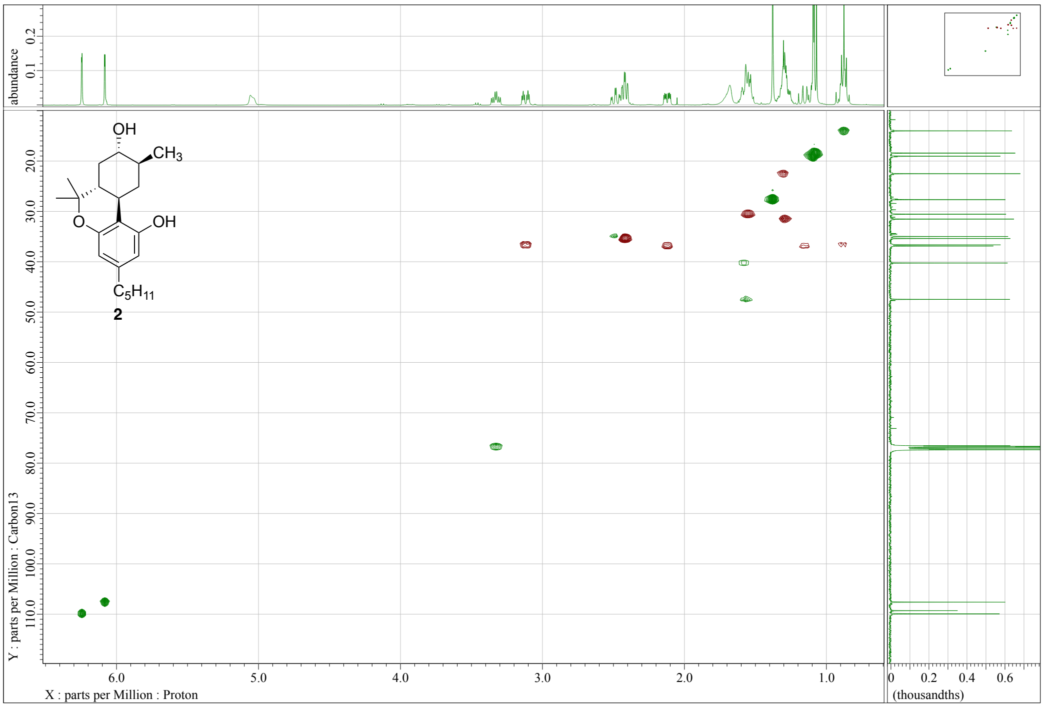

Figure S12. HMBC spectrum of **2**

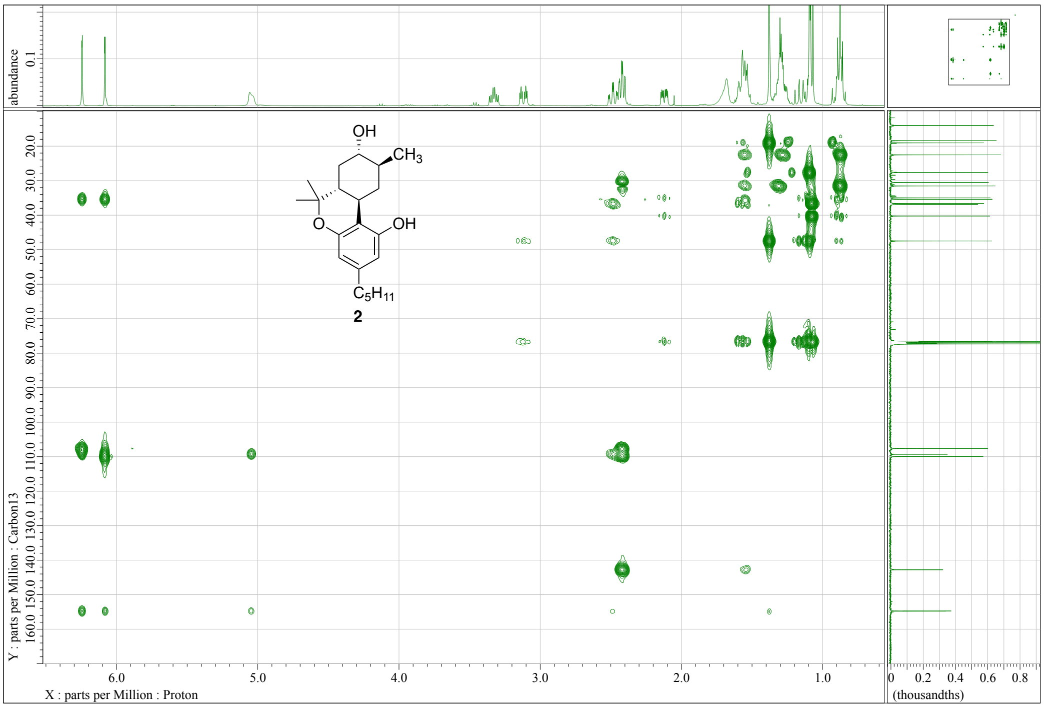

Table S2. Assignment of the peaks in the ^{1}H and ^{13}C NMR spectra of **2**.

	^{1}H	^{13}C		^{1}H	^{13}C
1		154	10	3.12 (td, 1H, 10α)	36.6
				1.77 (dtd, 1H, 10β)	
1-OH	5.06 (brs, 1H)		10a	2.48 (dt, 1H)	35.0
2	6.08 (d, 1H)	107.6	10b		109.3
3		142.8	11	1.08 (d, 3H)	19
4	6.24 (d, 1H)	109.9	12	1.09 (s, 3H)	19
4a		154	13	1.38 (s, 3H)	27.7
6		76	1’	2.44–2.40 (m, 2H)	35.4
6a	1.61–1.51 (m, 1H)	47.5	2’	1.61–1.51 (m, 2H)	30.6
7	1.20–1.10 (m, 1H, 7α)	36.9	3’	1.35–1.25 (m, 2H)	31.5
	2.12 (ddd, 1H, 7β)				
8	3.33 (dt, 1H)	76	4’	1.35–1.25 (m, 2H)	22.5
8-OH	1.68 (brs, 1H)		5’	0.88 (t, 3H)	14.0
9	1.61–1.51 (m, 1H)	40.3			

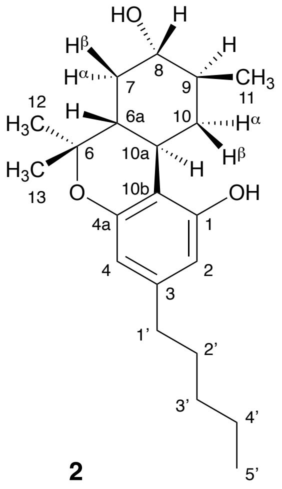

2

3. NMR Spectra of **3**

Figure S13. ^{1}H NMR (400 MHz, $CDCl_3$, ppm) of **3**

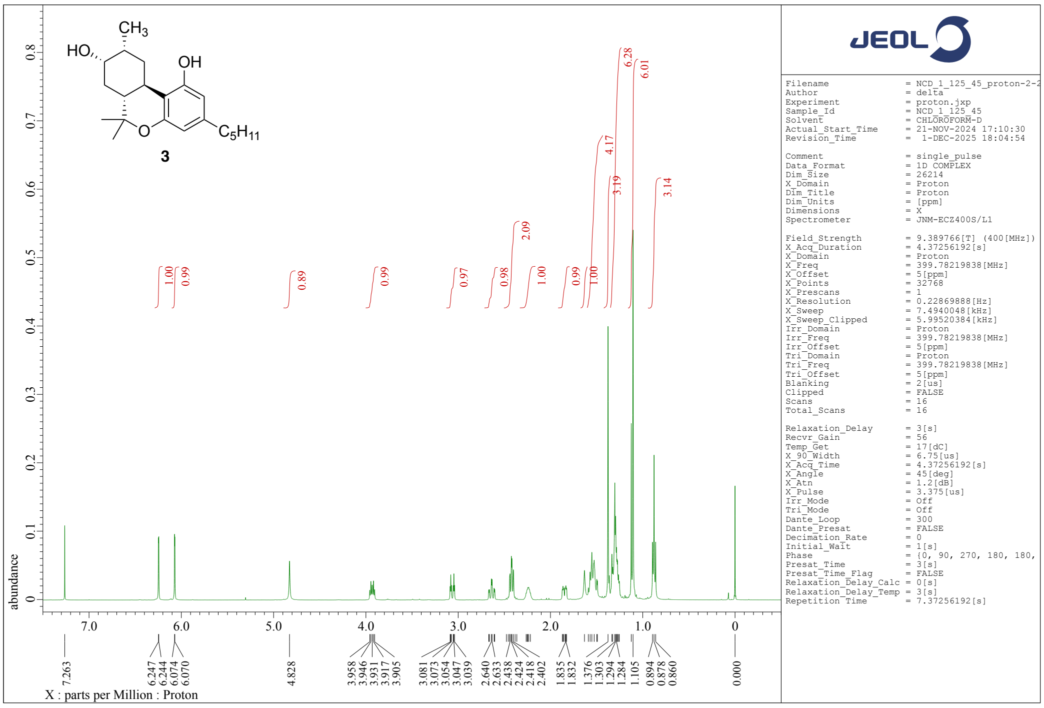

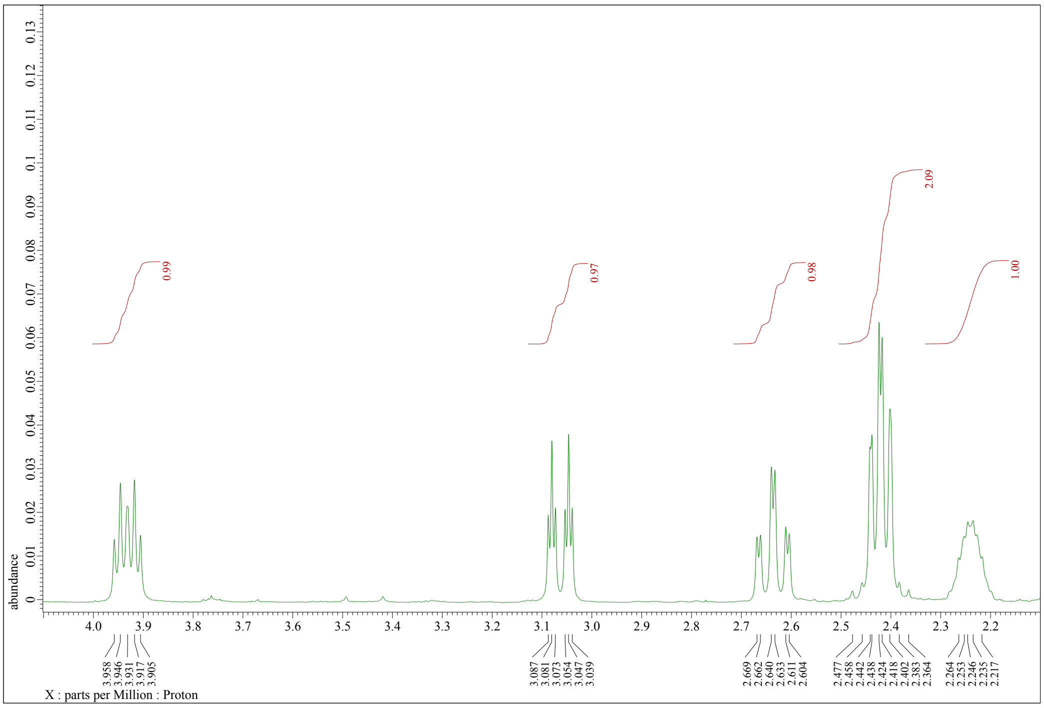

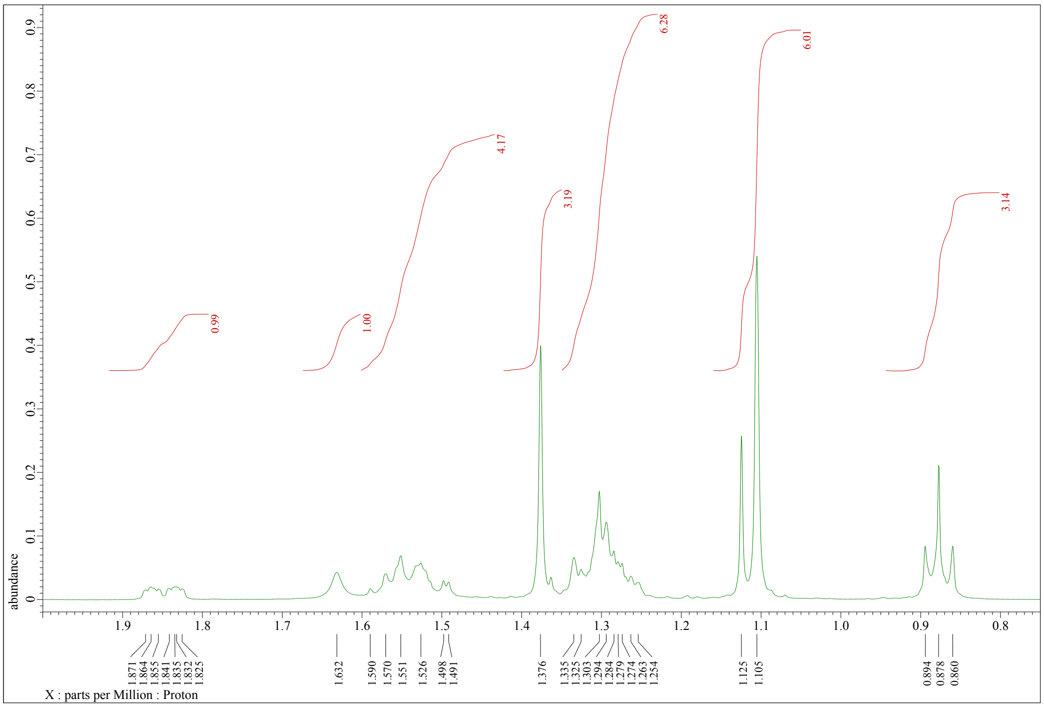

Figure S14. ^{13}C NMR (100 MHz, $CDCl_3$, ppm) of **3**

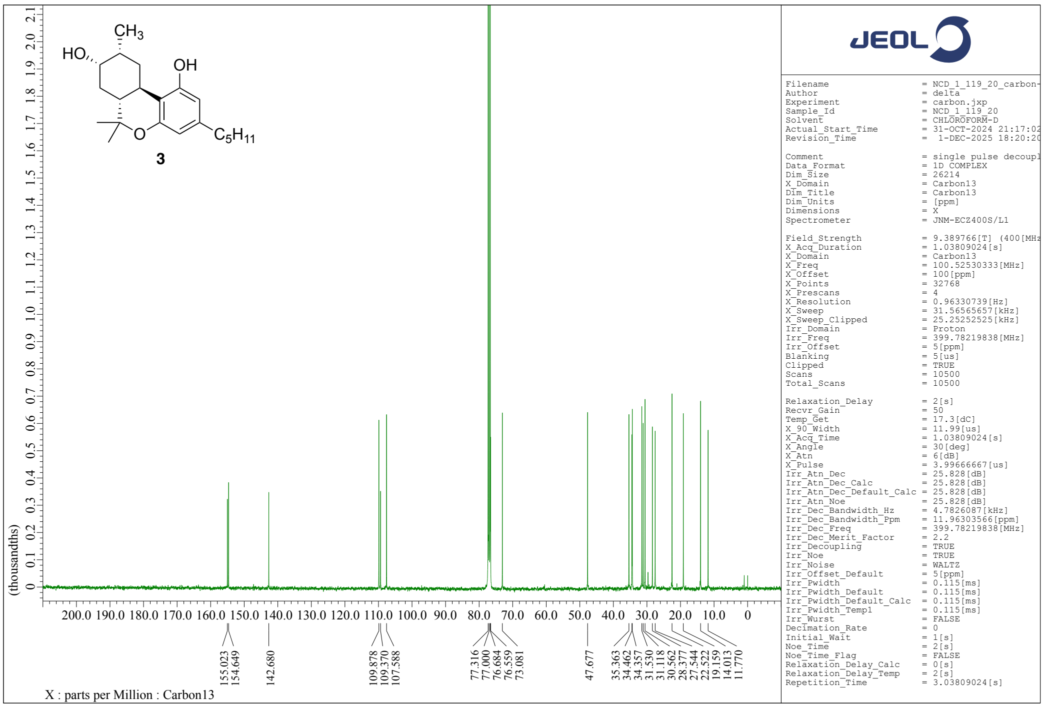

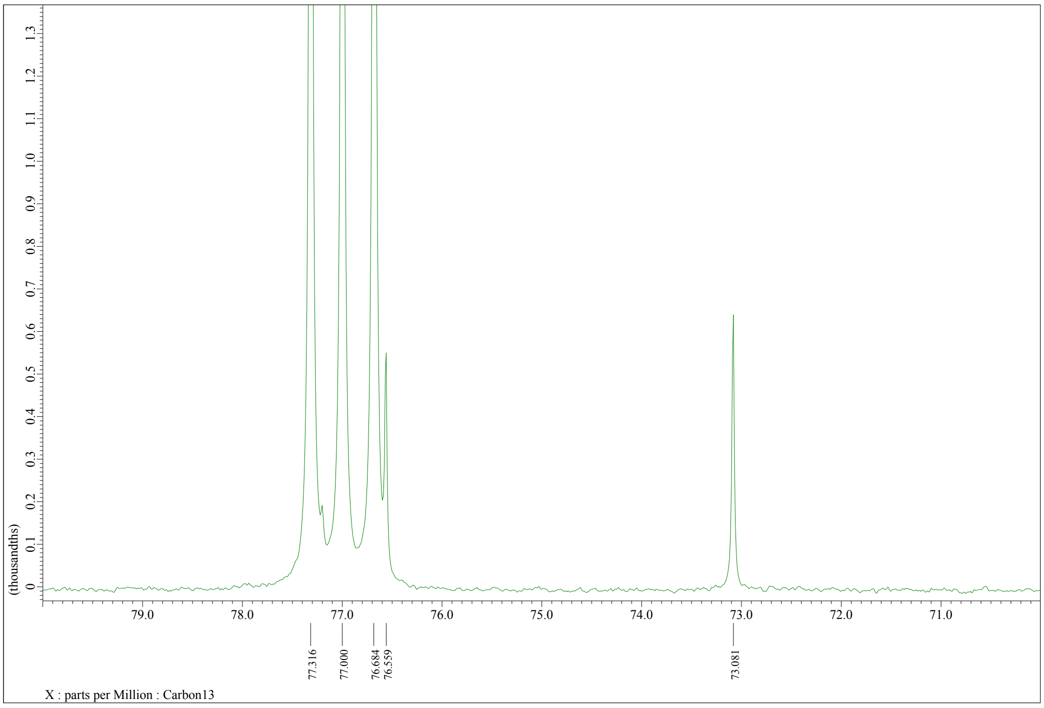
(thousandths)
77.316
77.000
76.684
76.559
73.081
X : parts per Million : Carbon13

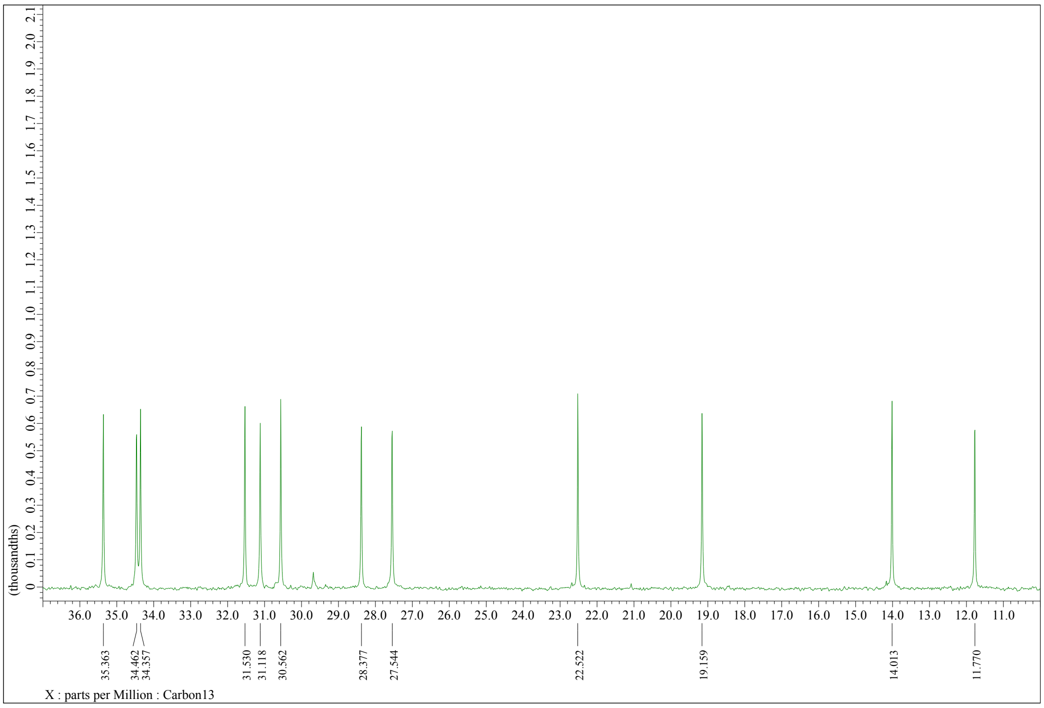
(thousandths)
35.363
34.462
34.357
31.530
31.118
30.562
28.377
27.544
22.522
19.159
14.013
11.770
X : parts per Million : Carbon13

Figure S15. NOESY spectrum of **3**

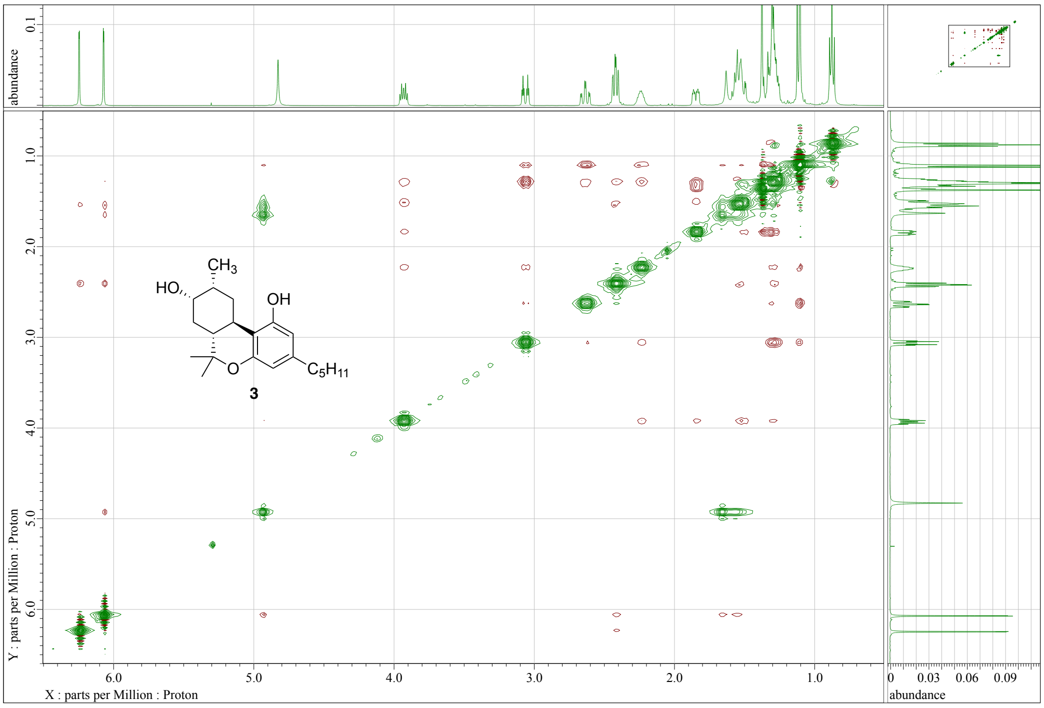

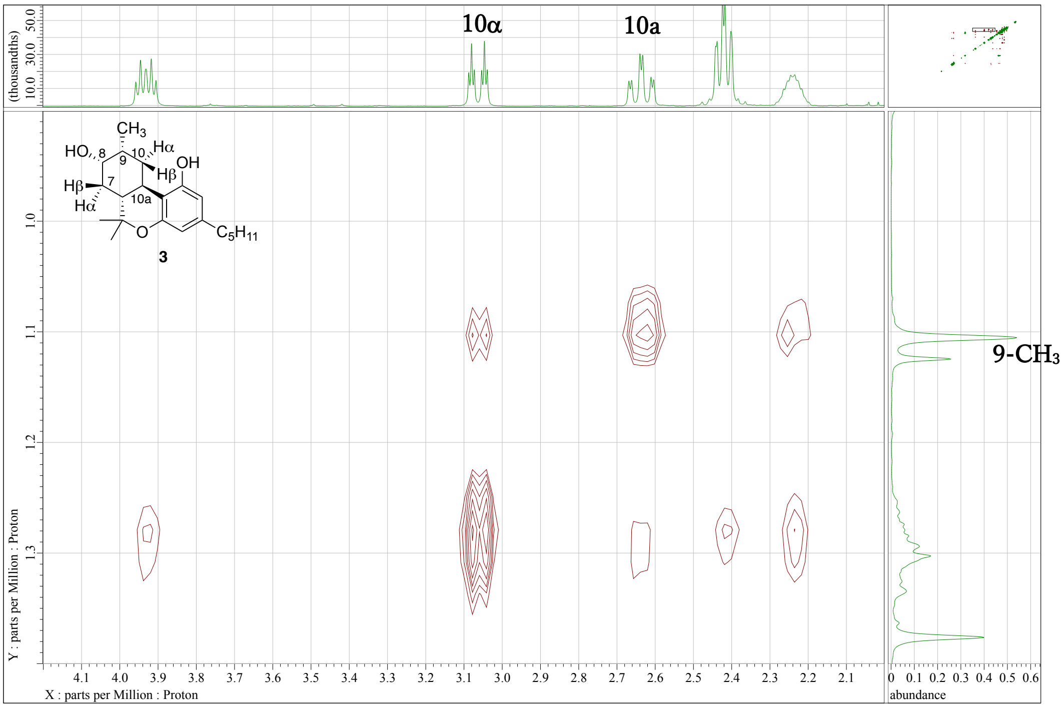

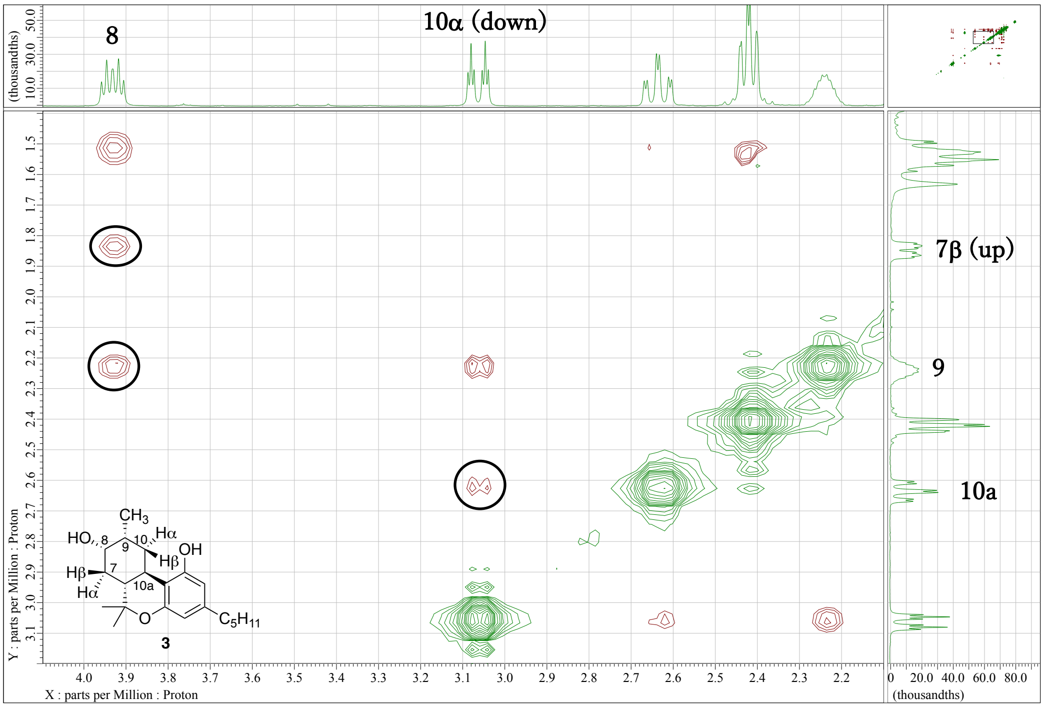

Figure S16. COSY spectrum of **3**

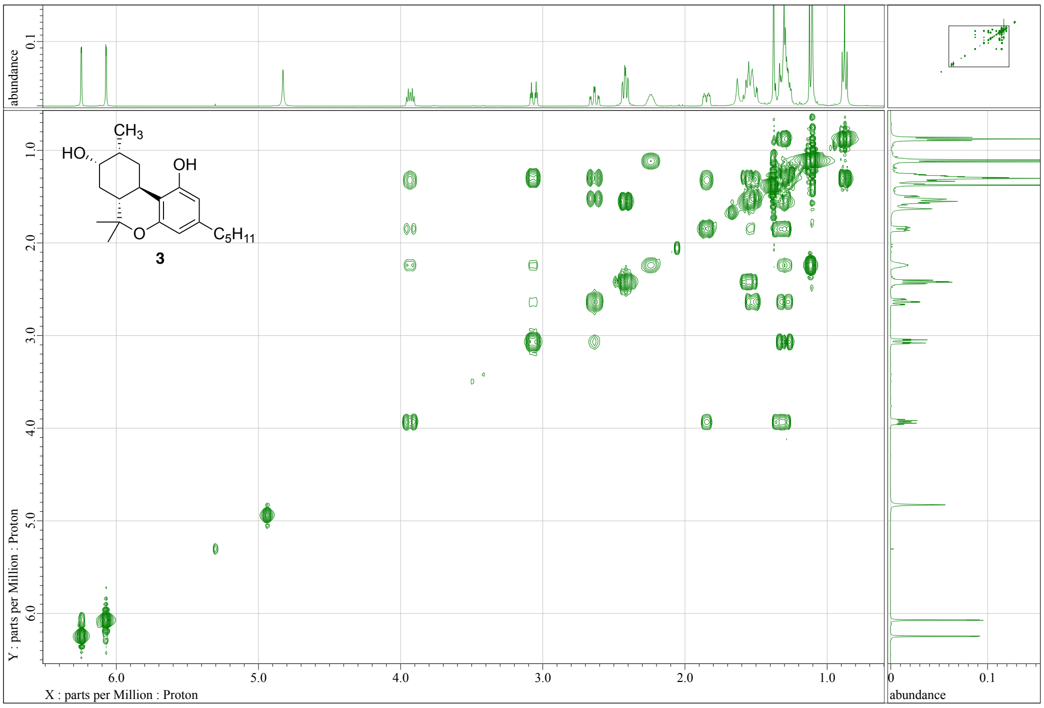

Figure S17. HSQC spectrum of **3**

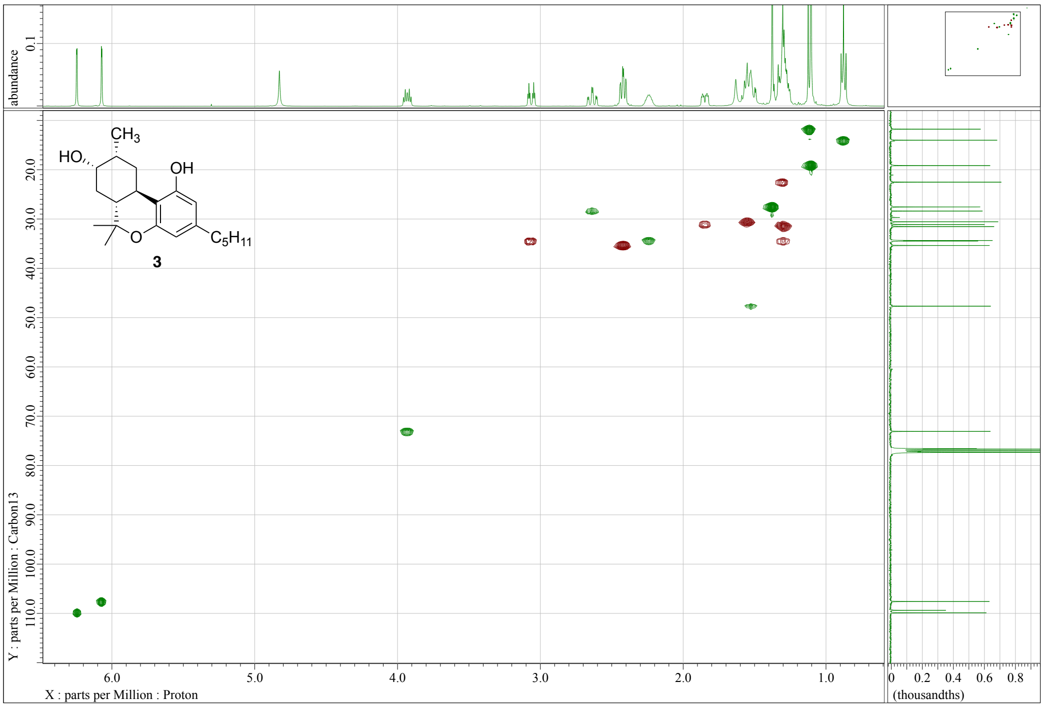

Figure S18. HMBC spectrum of **3**

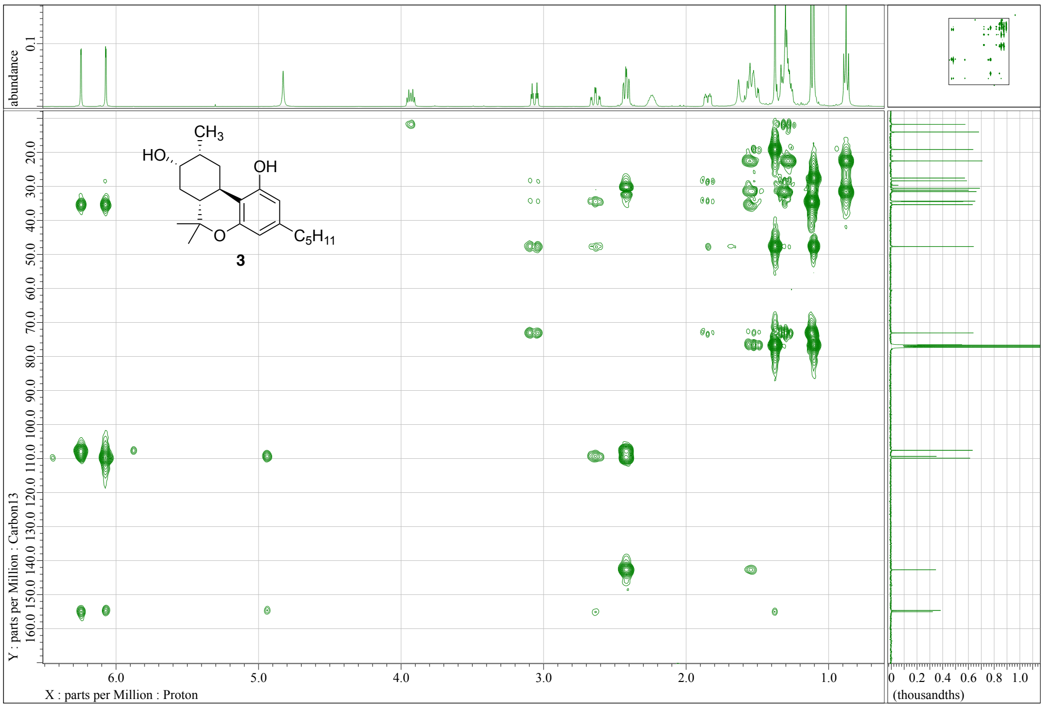

Table S3. Assignment of the peaks in the ^{1}H and ^{13}C NMR spectra of **3**.

	^{1}H	^{13}C		^{1}H	^{13}C
1		154.6	10	3.06 (td, 1H, 10α)	34
				1.34–1.25 (m, 1H, 10β)	
1-OH	4.83 (brs, 1H)		10a	2.64 (dt, 1H)	28.4
2	6.07 (d, 1H)	107.5	10b		107.6
3		142.7	11	1.12 (d, 3H)	11.8
4	6.25 (d, 1H)	109.9	12	1.11 (s, 3H)	19.2
4a		155.0	13	1.38 (s, 3H)	27.5
6		76.6	1’	2.44–2.40 (m, 2H)	35.4
6a	1.59–1.49 (m, 1H)	47.7	2’	1.59–1.49 (m, 2H)	30.6
7	1.34–1.25 (m, 1H, 7α)	31	3’	1.34–1.25 (m, 2H)	31
	1.88–1.82 (m, 1H, 7β)				
8	3.93 (td, 1H)	73.1	4’	1.34–1.25 (m, 2H)	22.5
8-OH	1.63 (brs, 1H)		5’	0.88 (t, 3H)	14.0
9	2.29–2.20 (m, 1H)	34			

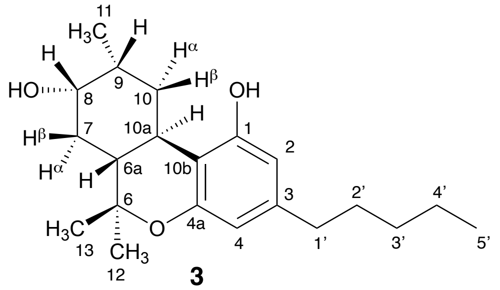

3

4. NMR Spectra of **4**

Figure S19. ^{1}H NMR (400 MHz, $CDCl_3$, ppm) of **4**

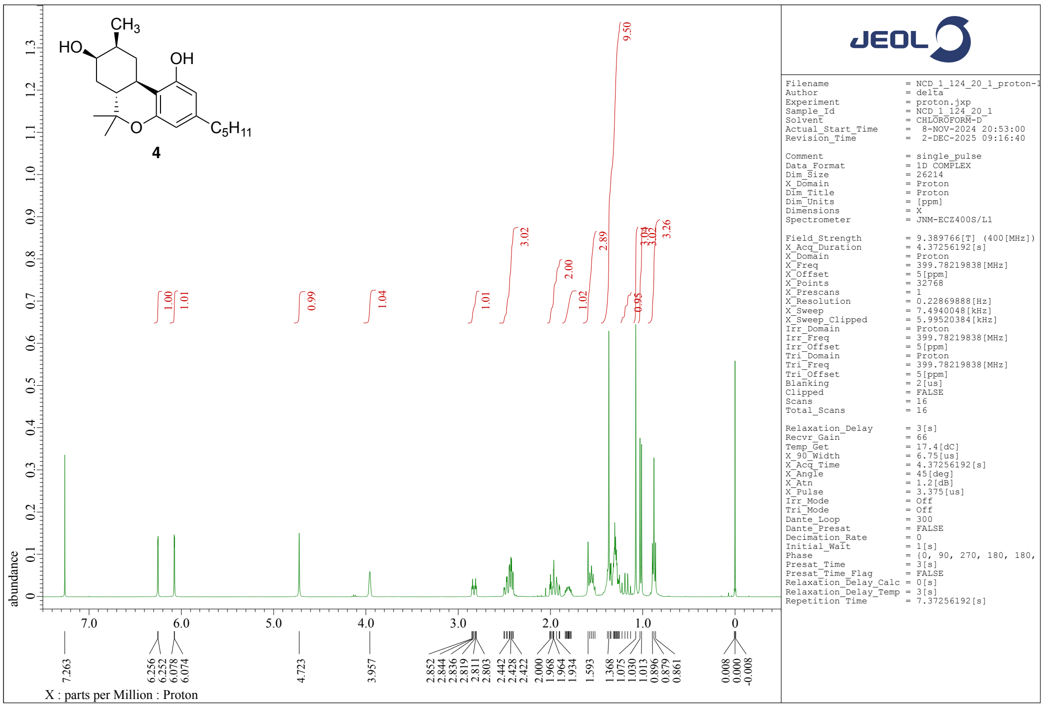

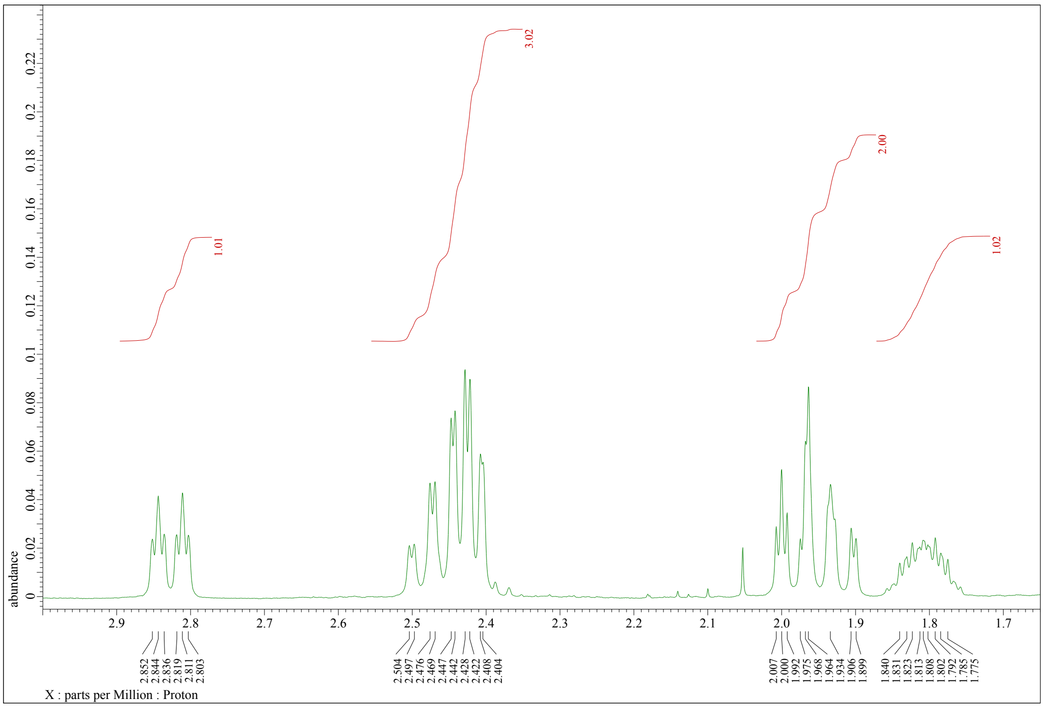

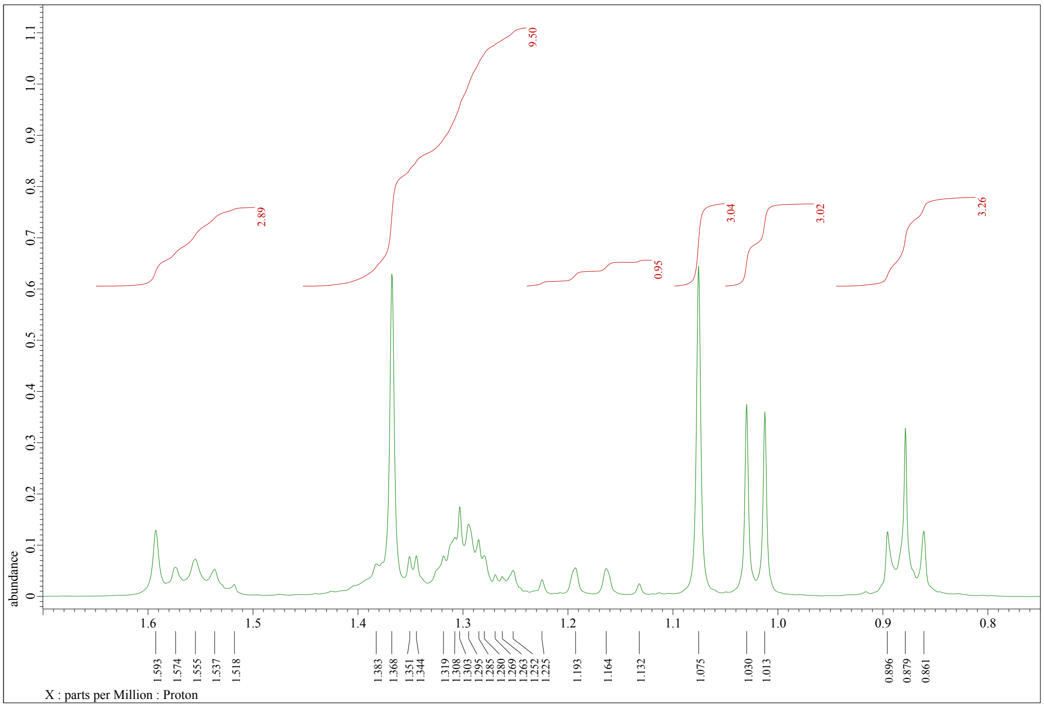

Figure S20. ^{13}C NMR (100 MHz, $CDCl_3$, ppm) of **4**

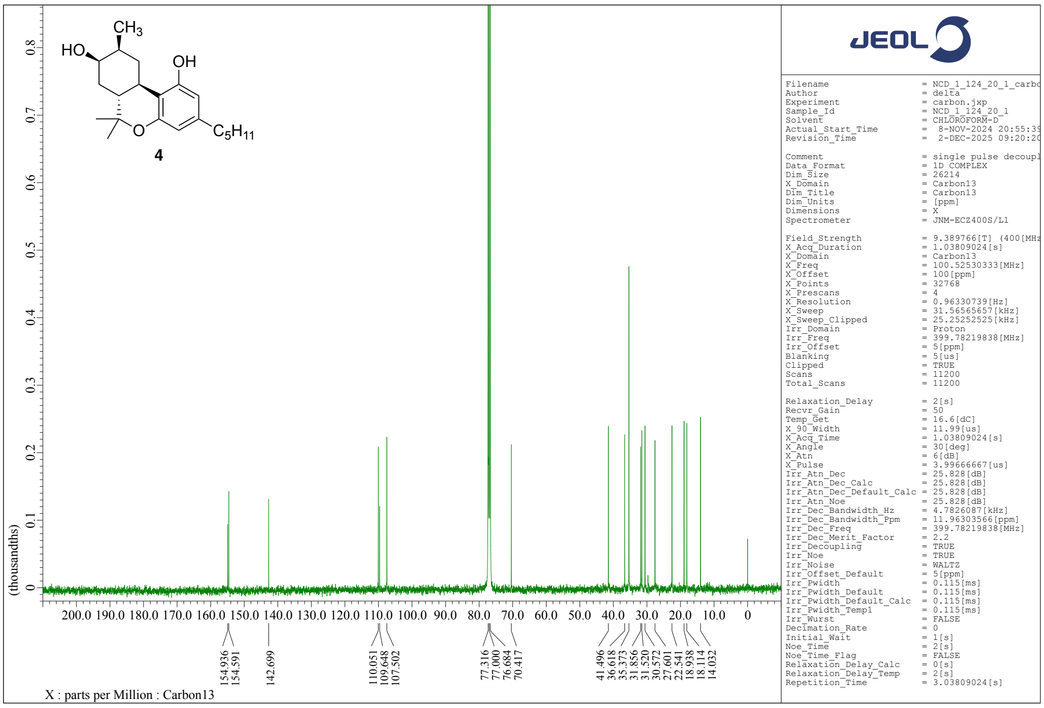

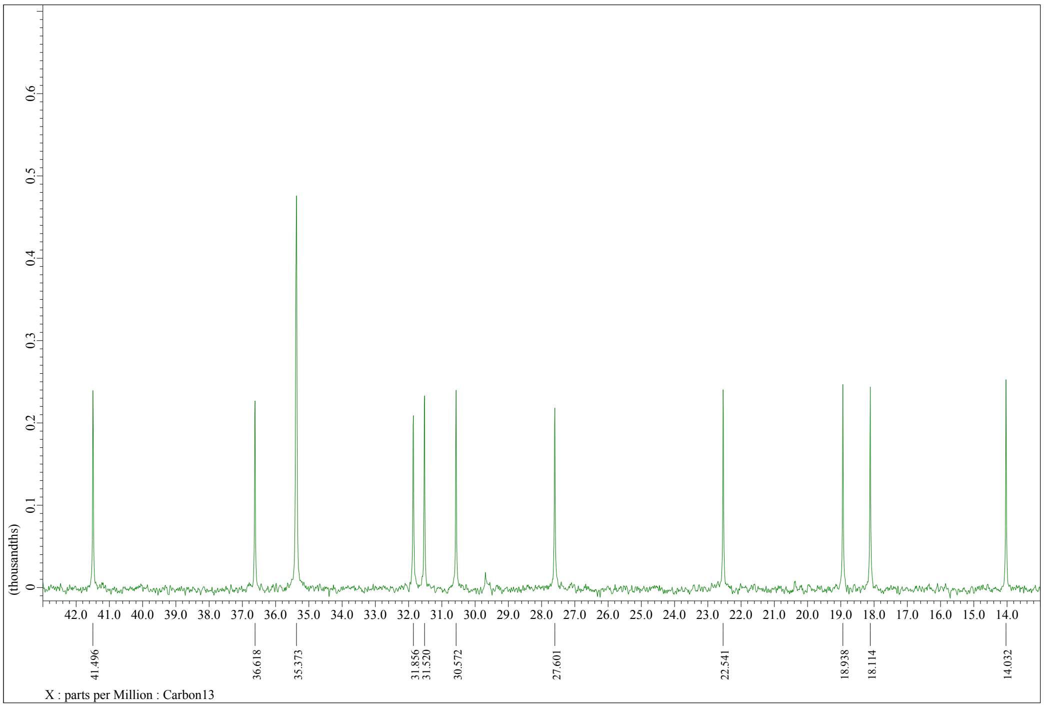

Figure S21. NOESY spectrum of **4**

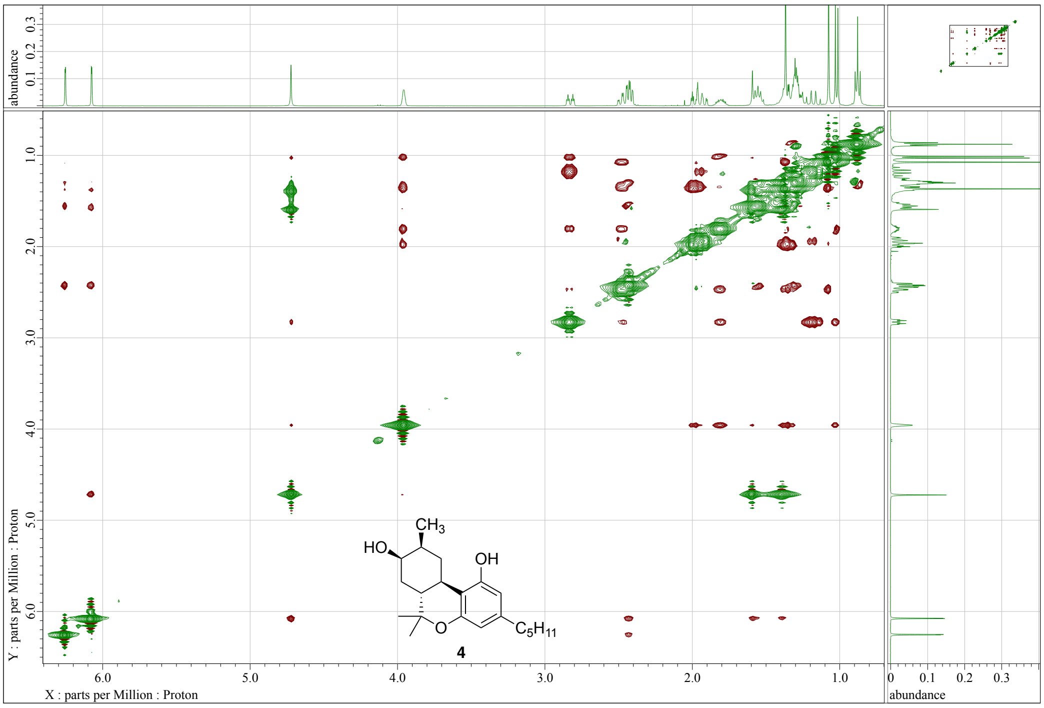

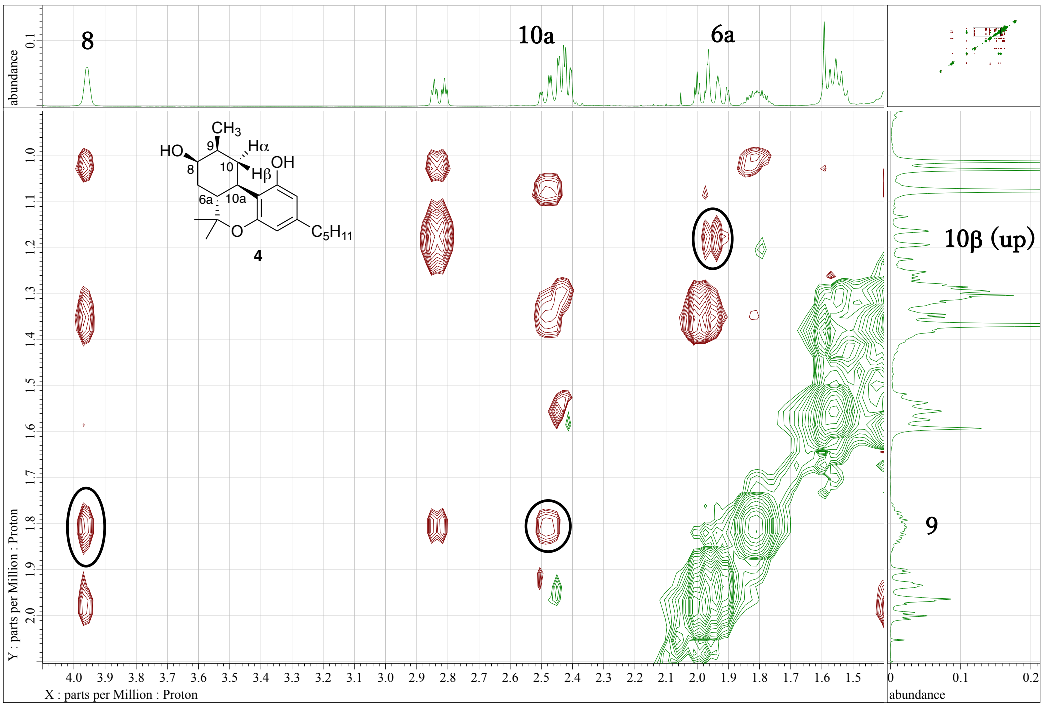

Figure S22. COSY spectrum of **4**

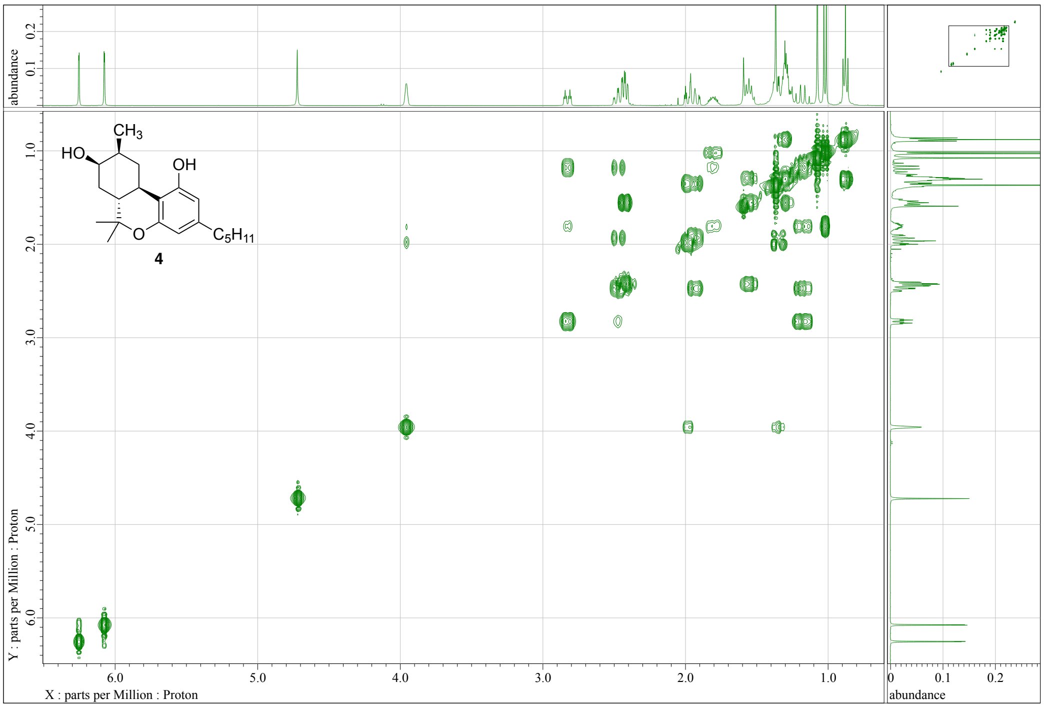

Figure S23. HSQC spectrum of **4**

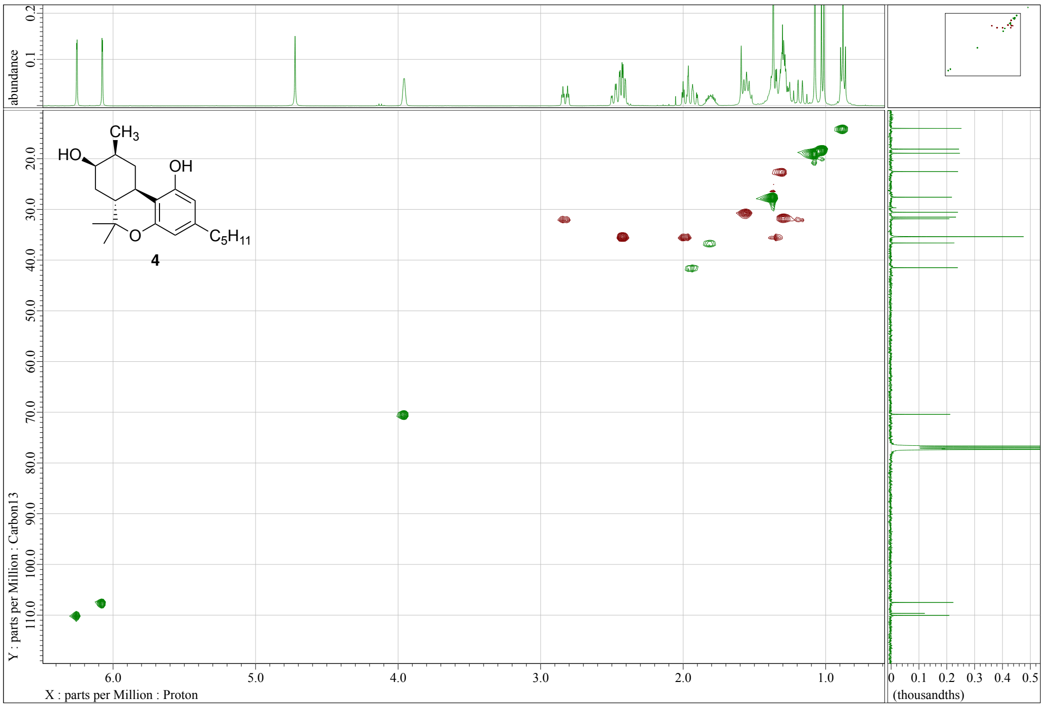

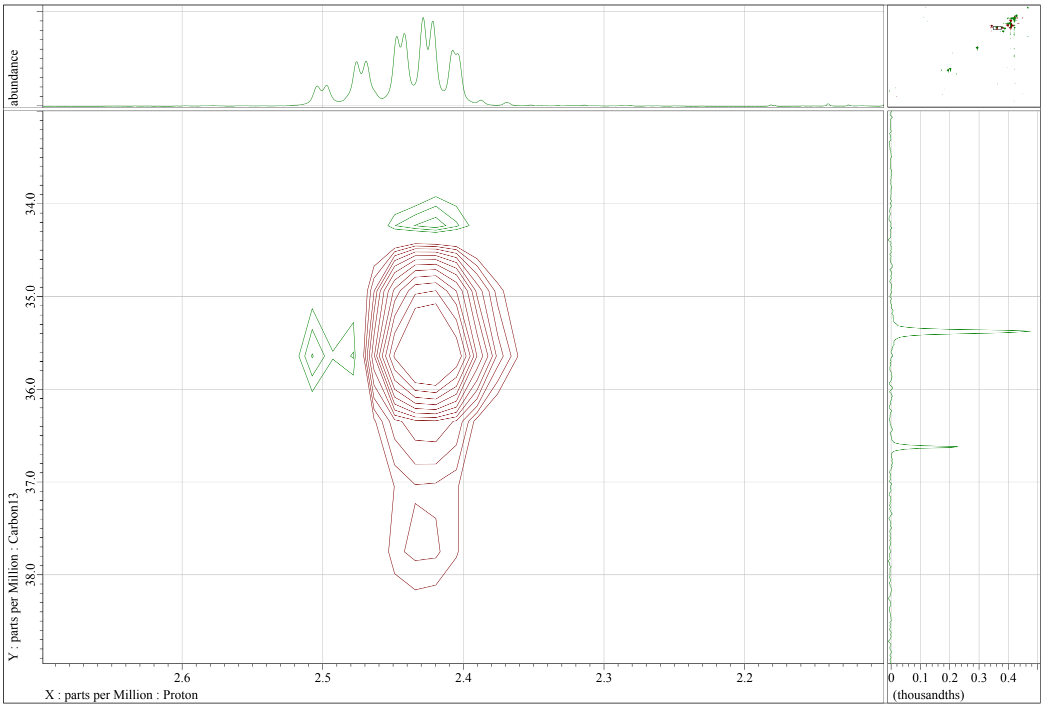

Figure S24. HMBC spectrum of **4**

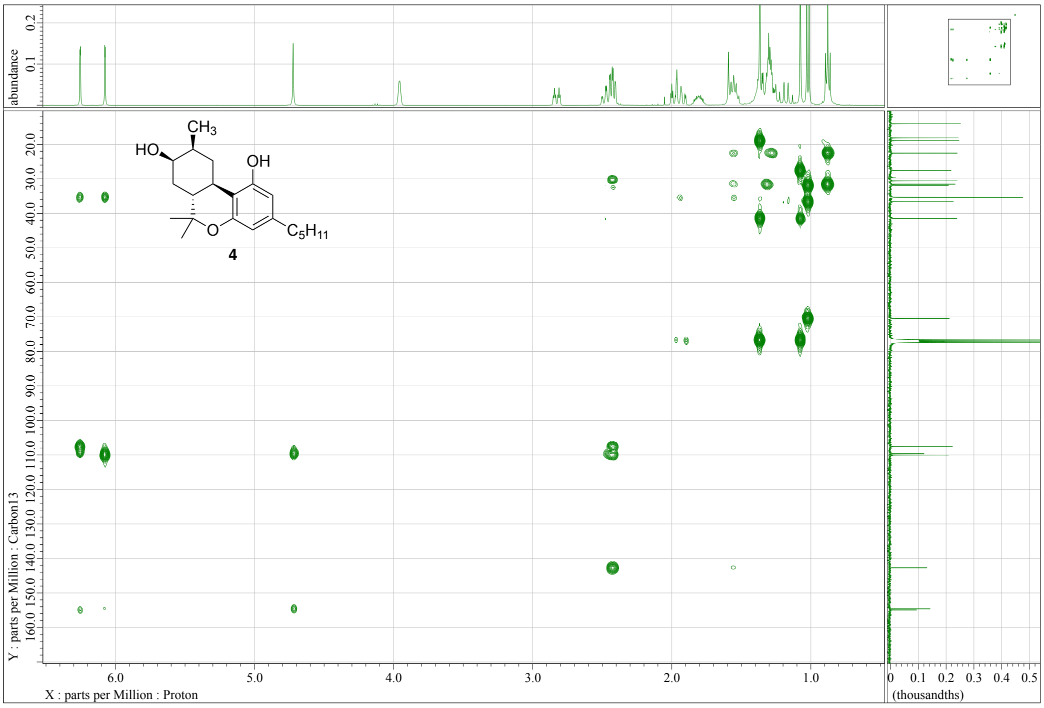

Table S4. Assignment of the peaks in the ^{1}H and ^{13}C NMR spectra of **4**.

	^{1}H	^{13}C		^{1}H	^{13}C
1		154.6	10	2.83 (td, 1H, 10α)	31.9
				1.23–1.13 (m, 1H, 10β)	
1-OH	4.72 (brs, 1H)		10a	2.50–2.40 (m, 1H)	35.4
2	6.08 (d, 1H)	107.5	10b		109.6
3		142.7	11	1.02 (d, 3H)	18.1
4	6.25 (d, 1H)	110.1	12	1.08 (s, 3H)	18.9
4a		154.9	13	1.37 (s, 3H)	27.6
6		77.2	1’	2.50–2.40 (m, 2H)	35.4
6a	2.01–1.90 (m, 1H)	41.5	2’	1.59–1.52 (m, 2H)	30.6
7	1.41–1.25 (m, 1H, 7α)	35.4	3’	1.41–1.25 (m, 2H)	31.5
	2.01–1.90 (m, 1H, 7β)				
8	3.98–3.94 (m, 1H)	70.4	4’	1.41–1.25 (m, 2H)	22.5
8-OH	1.59–1.52 (m, 1H)		5’	0.88 (t, 3H)	14.0
9	1.86–1.76 (m, 1H)	36.6			

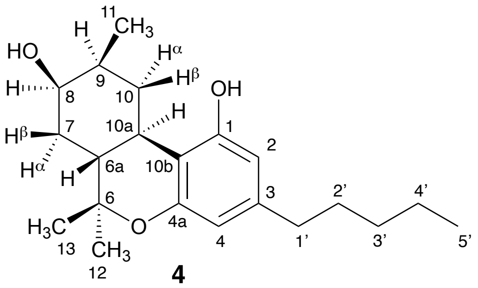

4

5. NMR Spectra of **5**

Figure S25. ^{1}H NMR (400 MHz, $CDCl_3$, ppm) of **5**

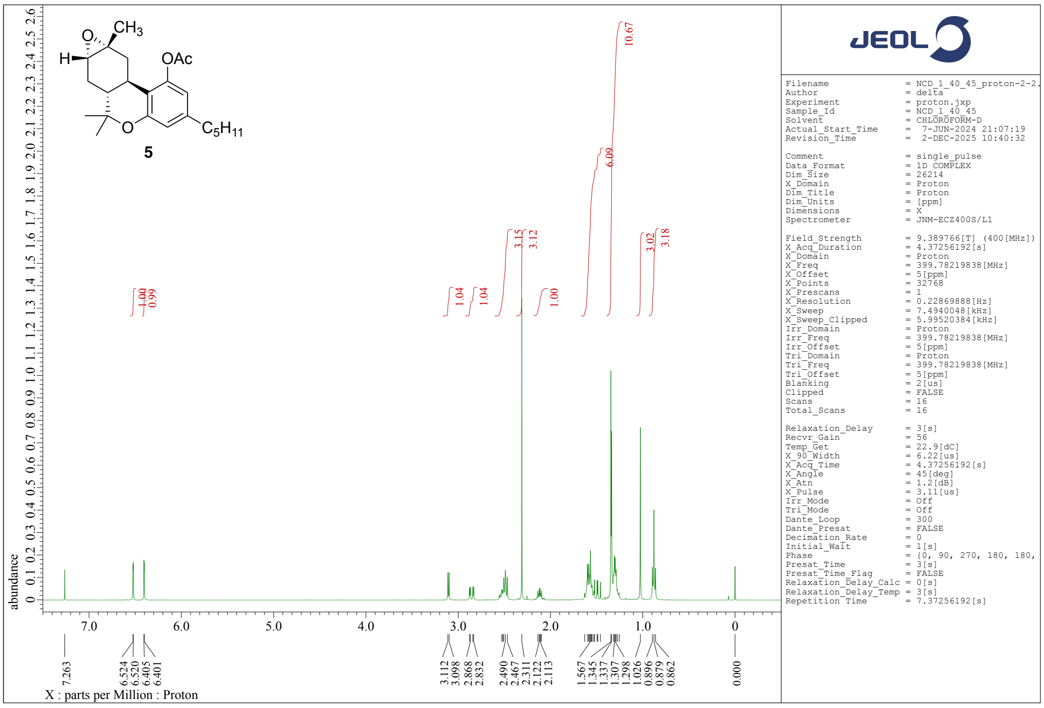

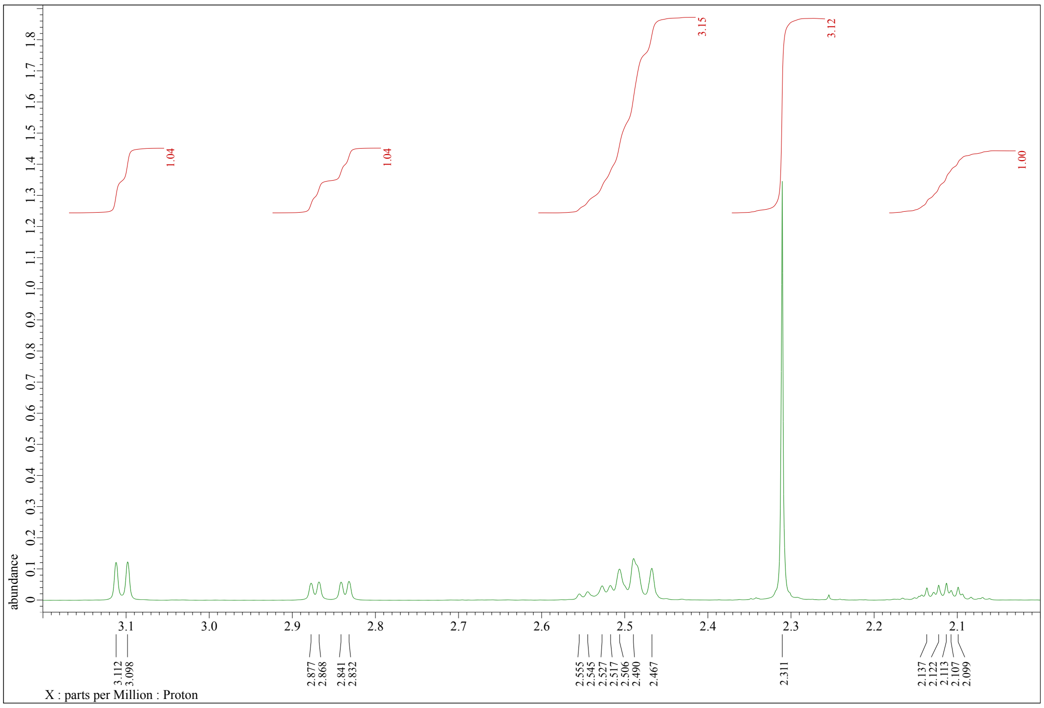

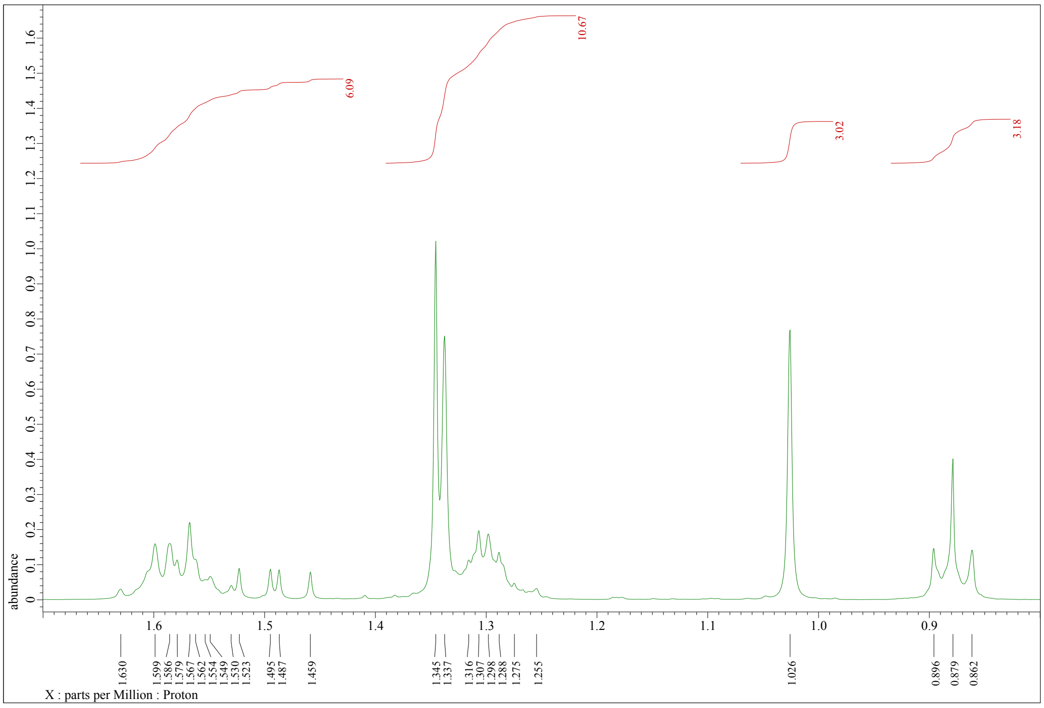

Figure S26. ^{13}C NMR (100 MHz, $CDCl_3$, ppm) of **5**

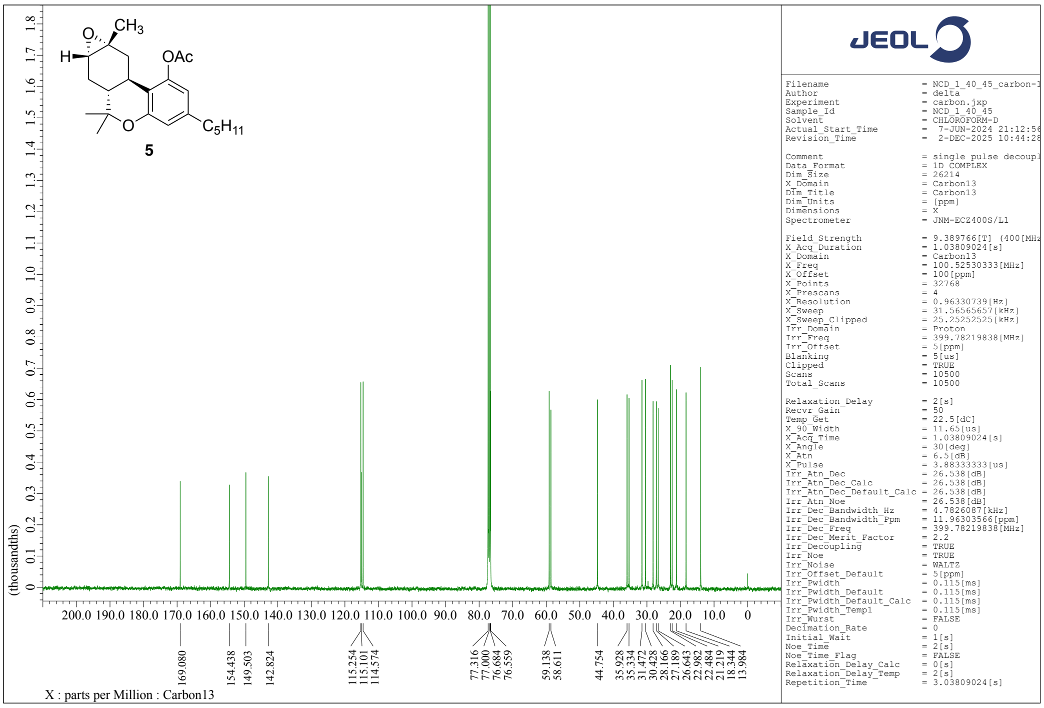

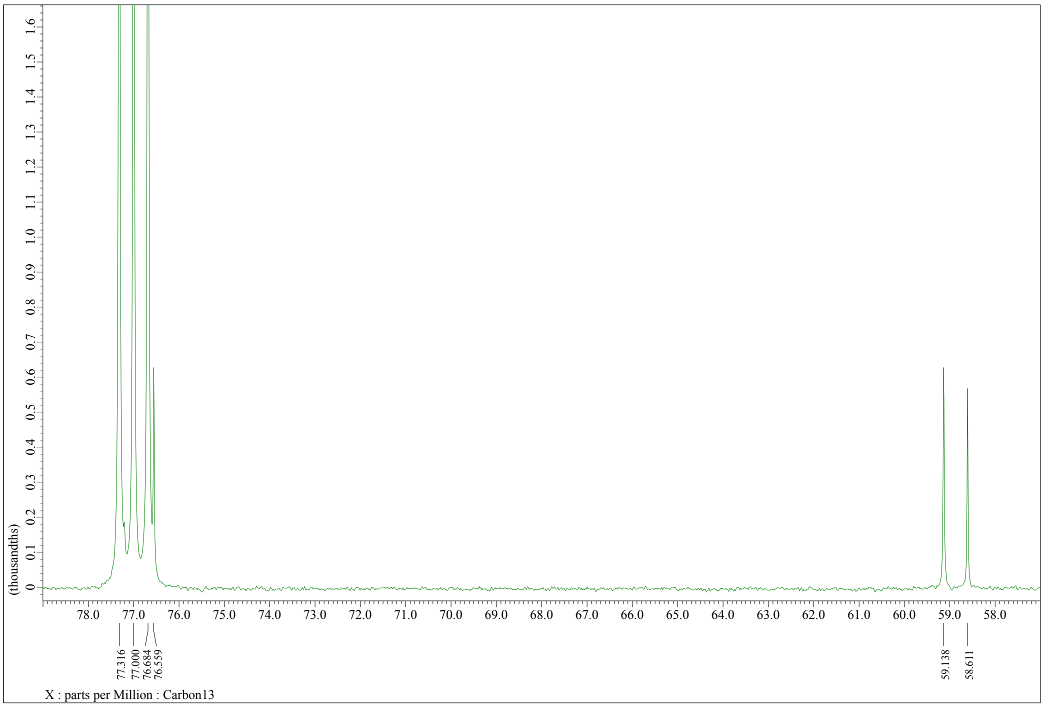
(thousandths)
X : parts per Million : Carbon13
77.316
77.000
76.684
76.559
59.138
58.611

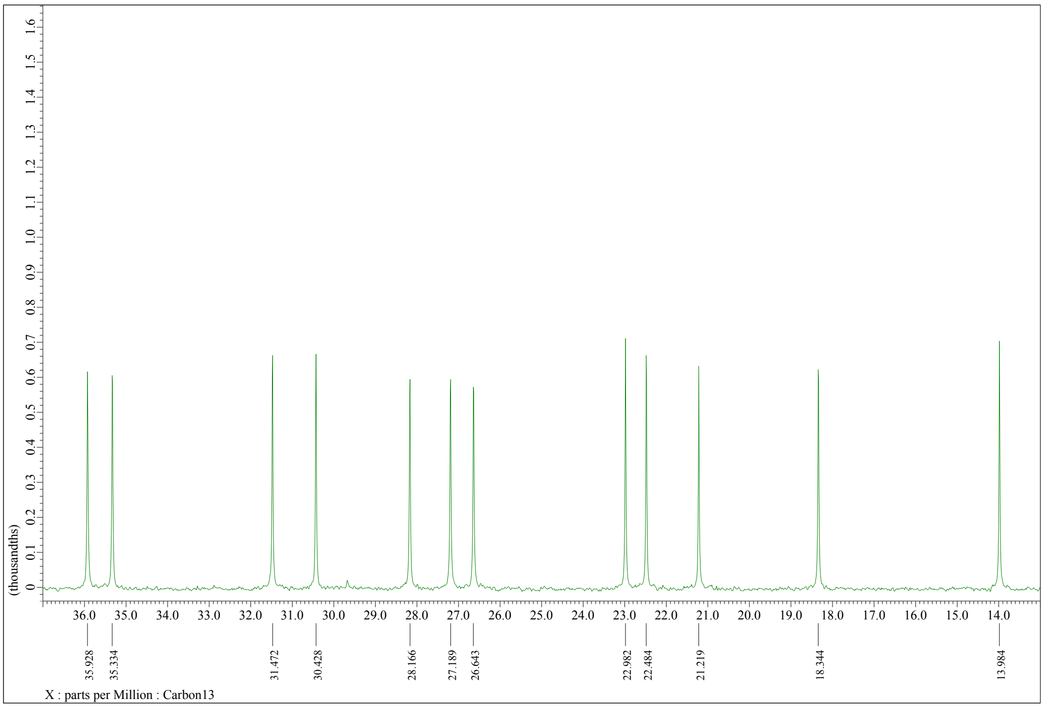
(thousandths)
X : parts per Million : Carbon13
35.928
35.334
31.472
30.428
28.166
27.189
26.643
22.982
22.484
21.219
18.344
13.984

6. NMR Spectra of **6**

Figure S27. ^{1}H NMR (400 MHz, $CDCl_3$, ppm) of **6**

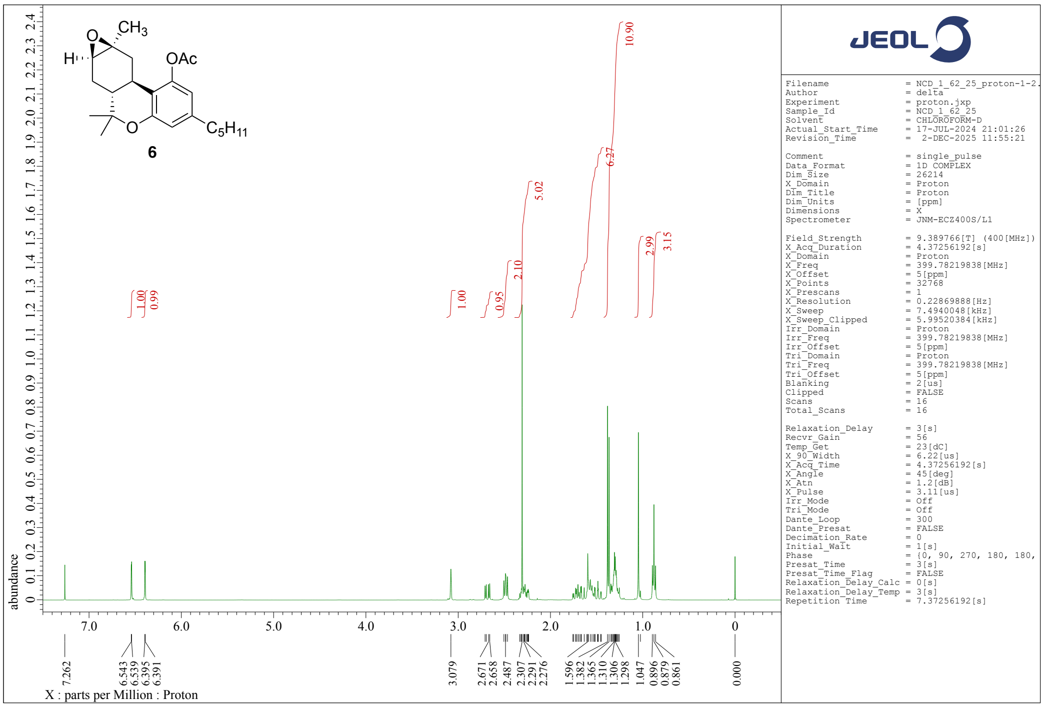

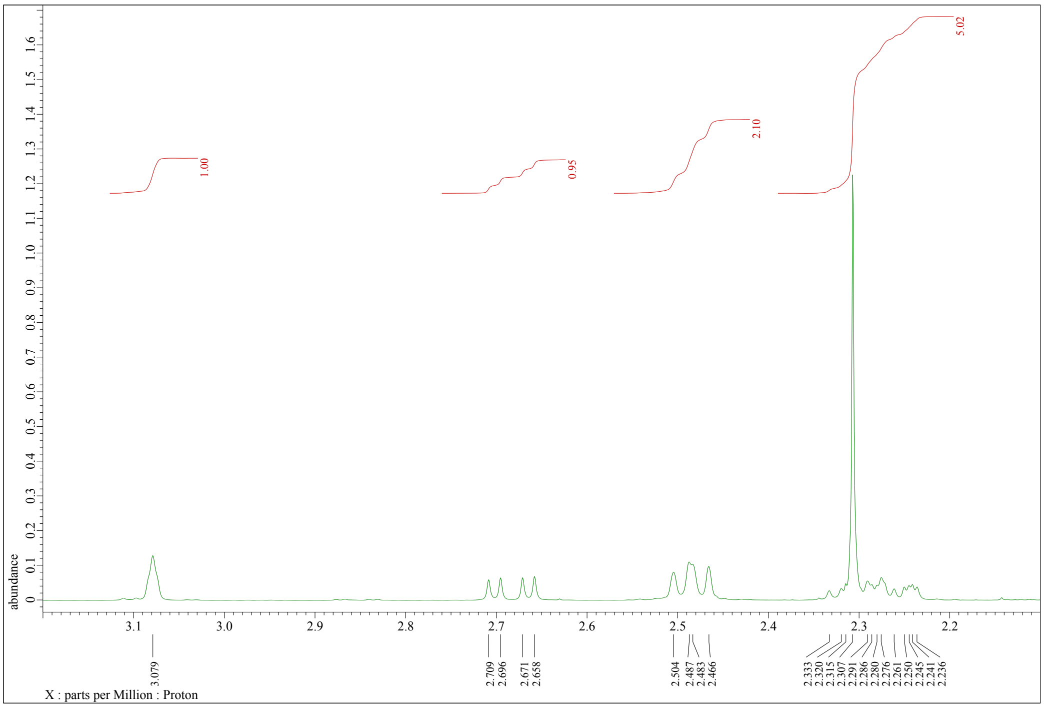

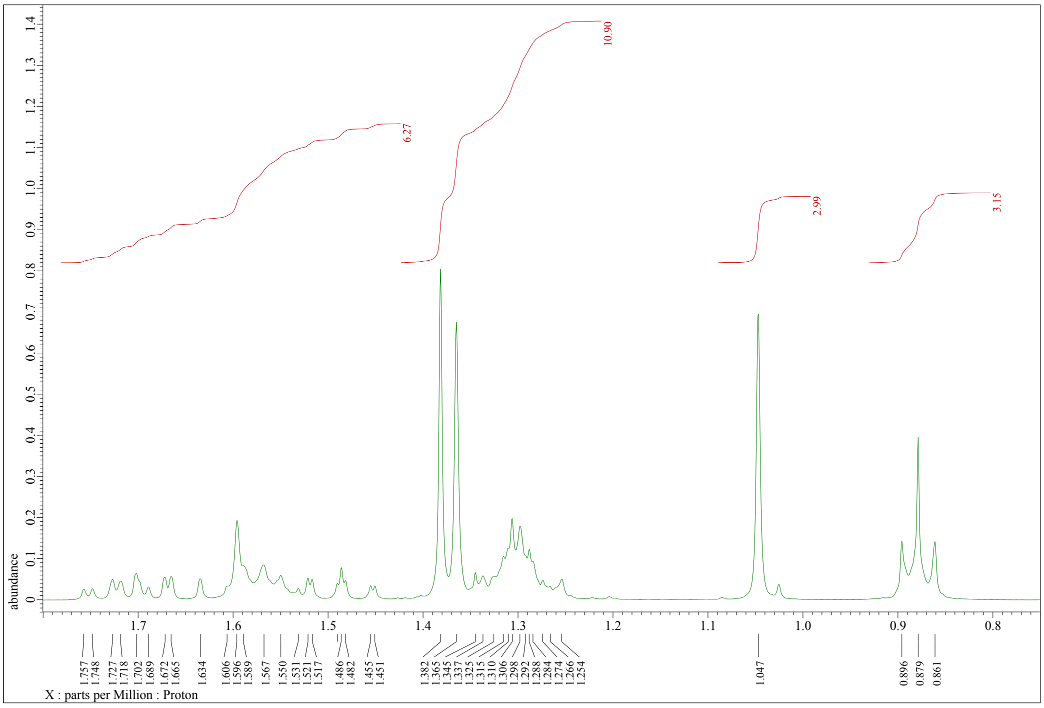

Figure S28. ^{13}C NMR (100 MHz, $CDCl_3$, ppm) of **6**

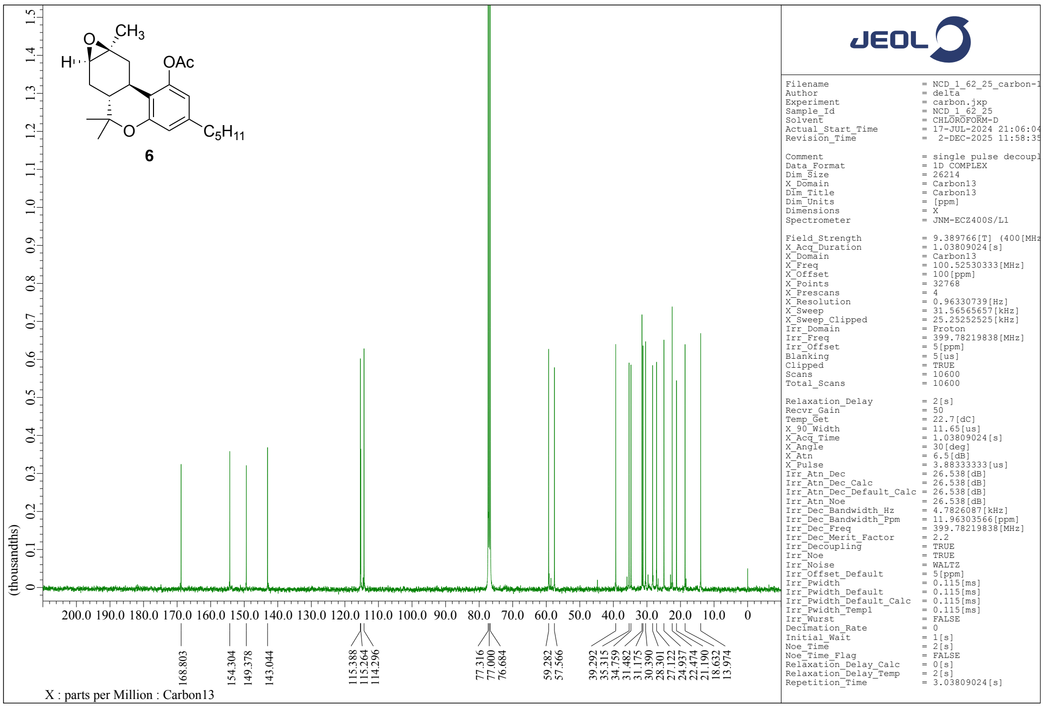

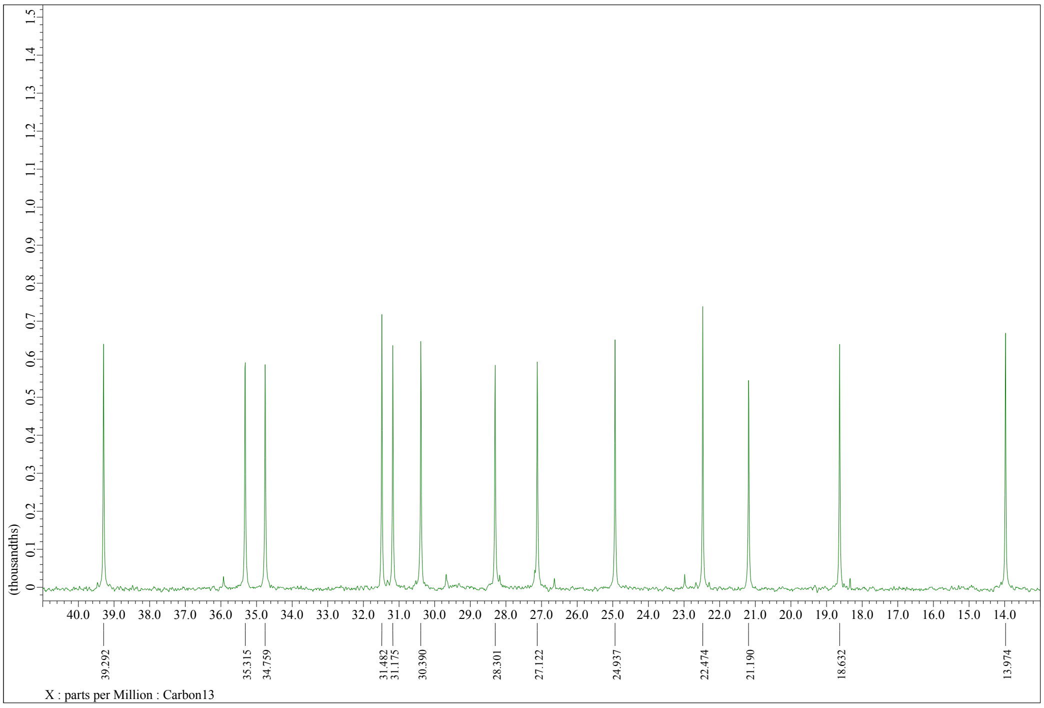
(thousandths)
39.292
35.315
34.759
31.482
31.175
30.390
28.301
27.122
24.937
22.474
21.190
18.632
13.974
X : parts per Million : Carbon13

7. NMR Spectra of **7**

Figure S29. ^{1}H NMR (400 MHz, $CDCl_3$, ppm) of **7**

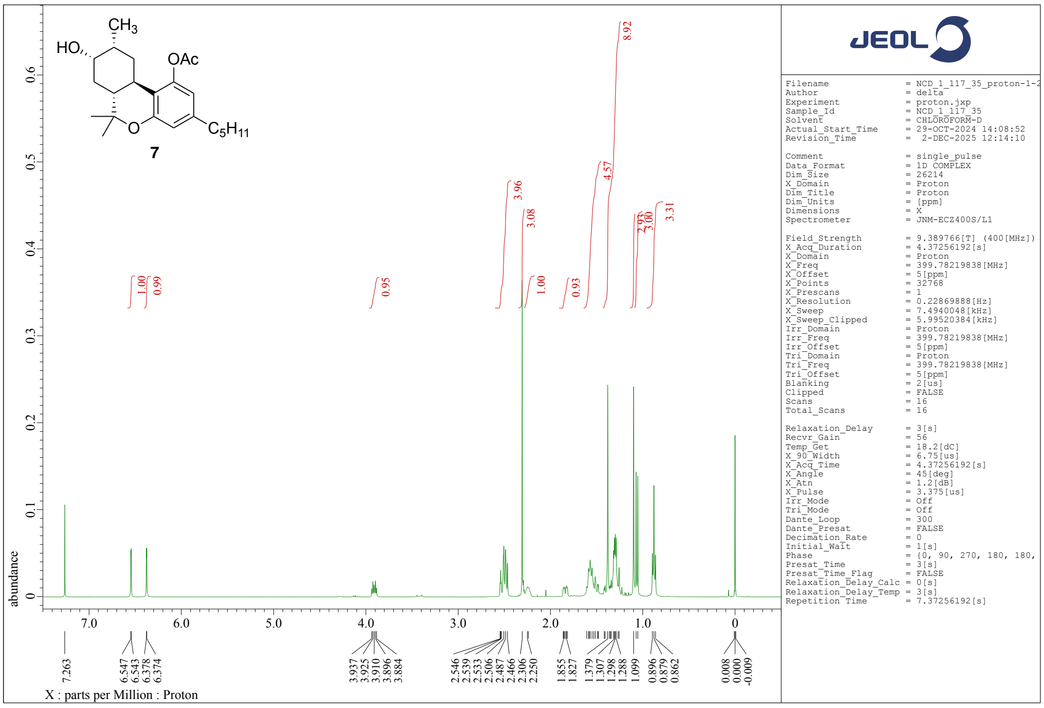

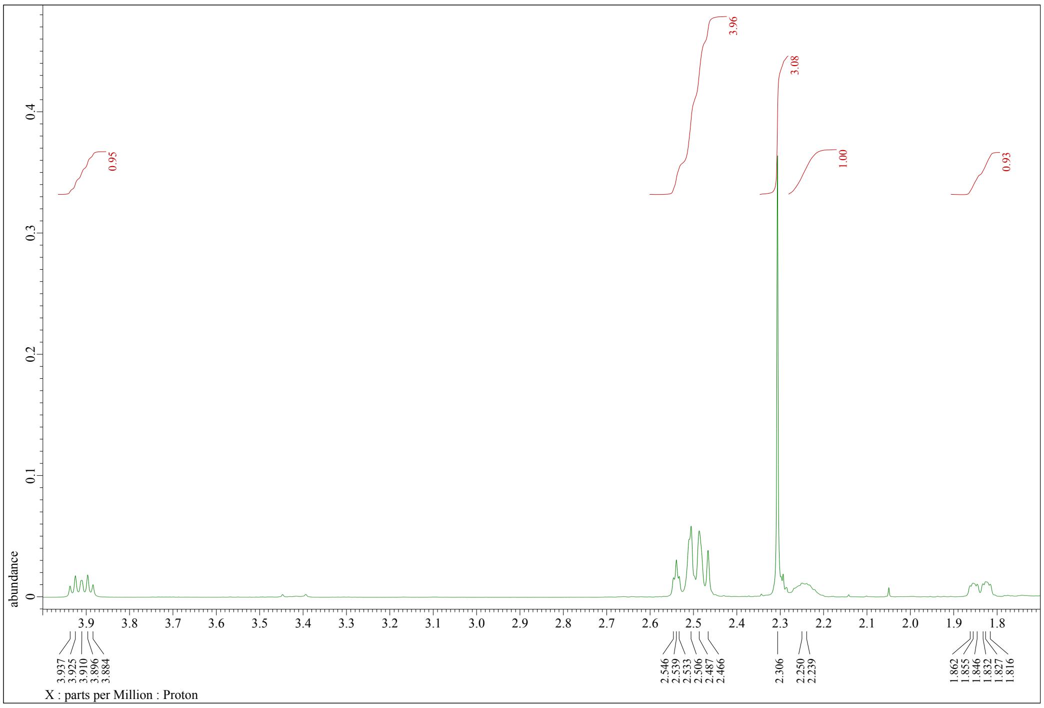

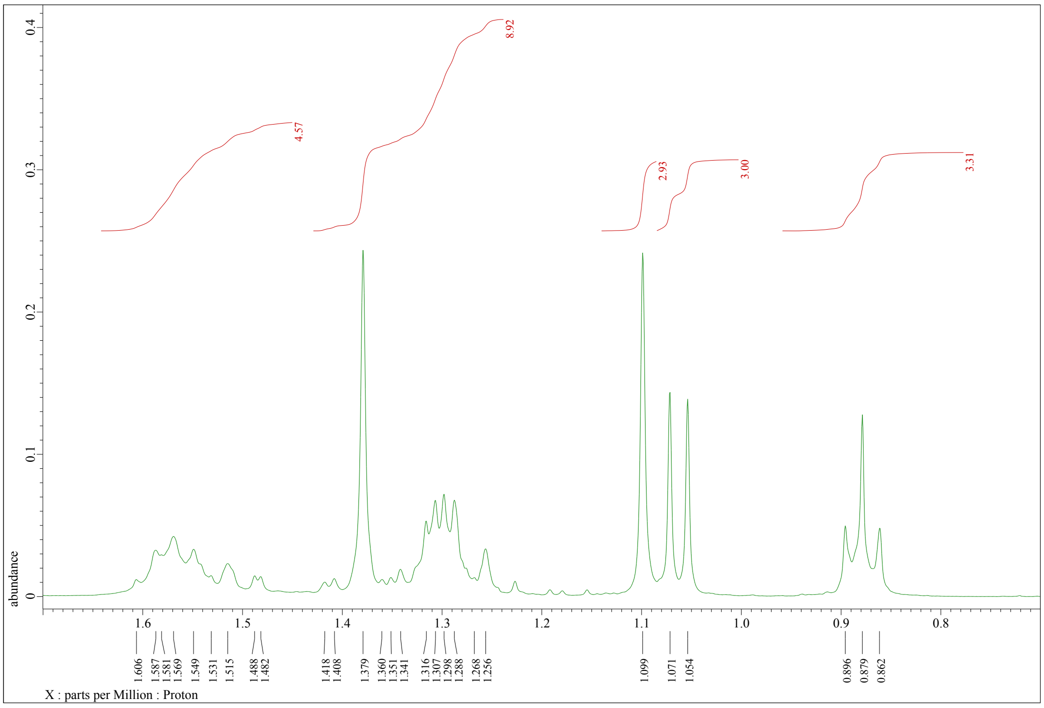

Figure S30. ^{13}C NMR (100 MHz, $CDCl_3$, ppm) of **7**

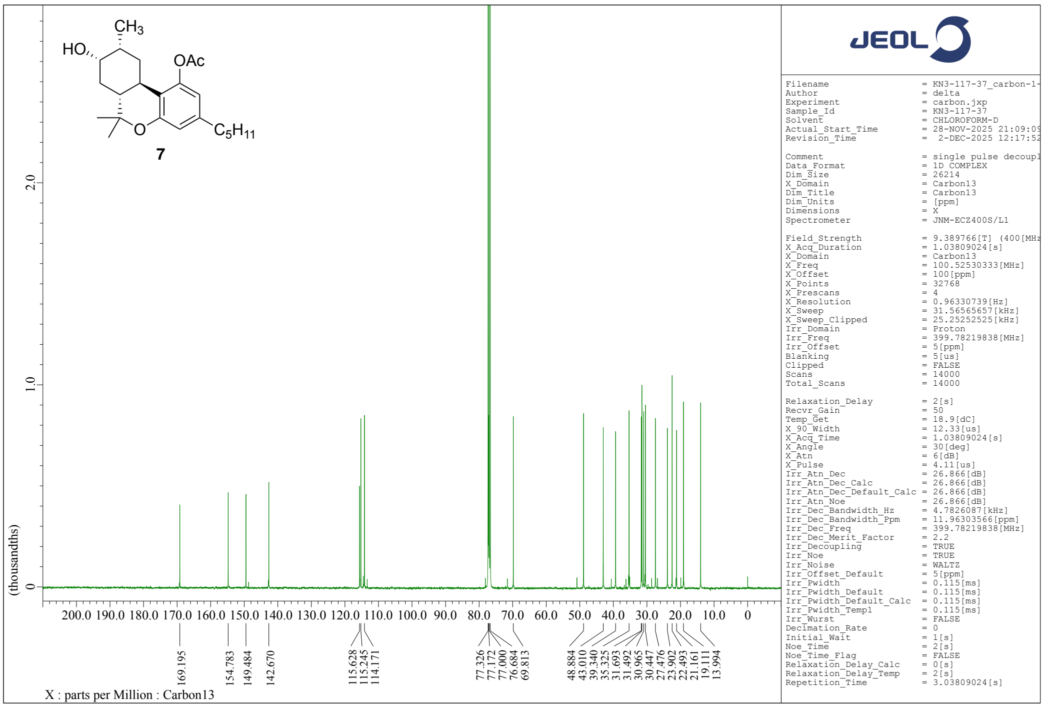

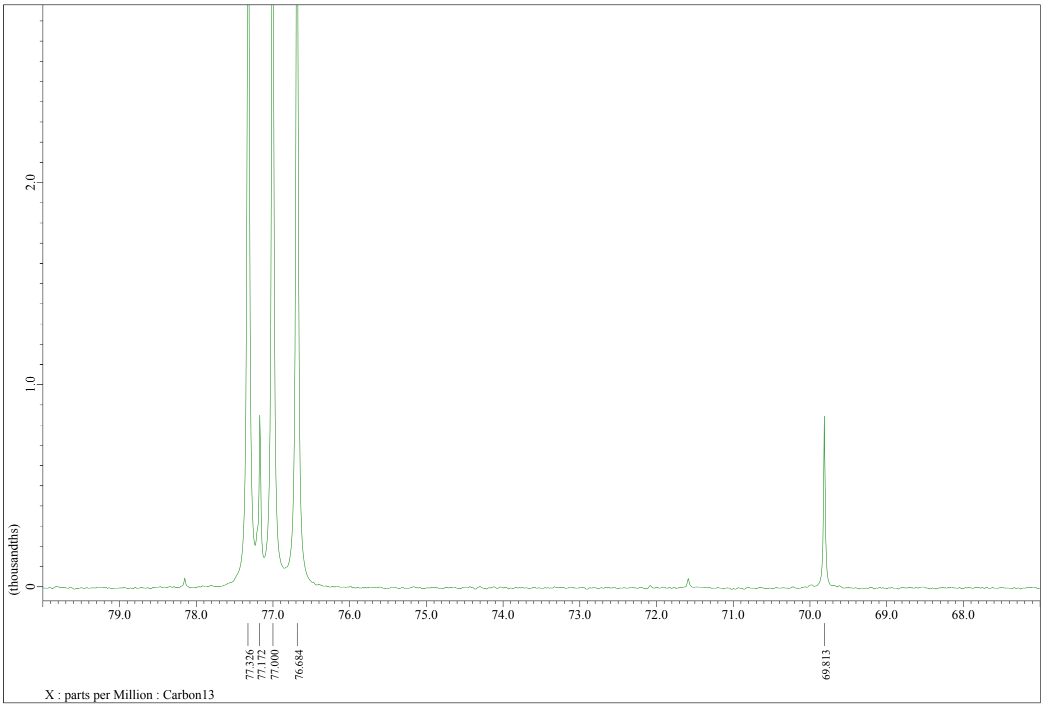
(thousandths)
0
1.0
2.0
79.0
78.0
77.0
76.0
75.0
74.0
73.0
72.0
71.0
70.0
69.0
68.0
77.326
77.172
77.000
76.684
69.813
X : parts per Million : Carbon13

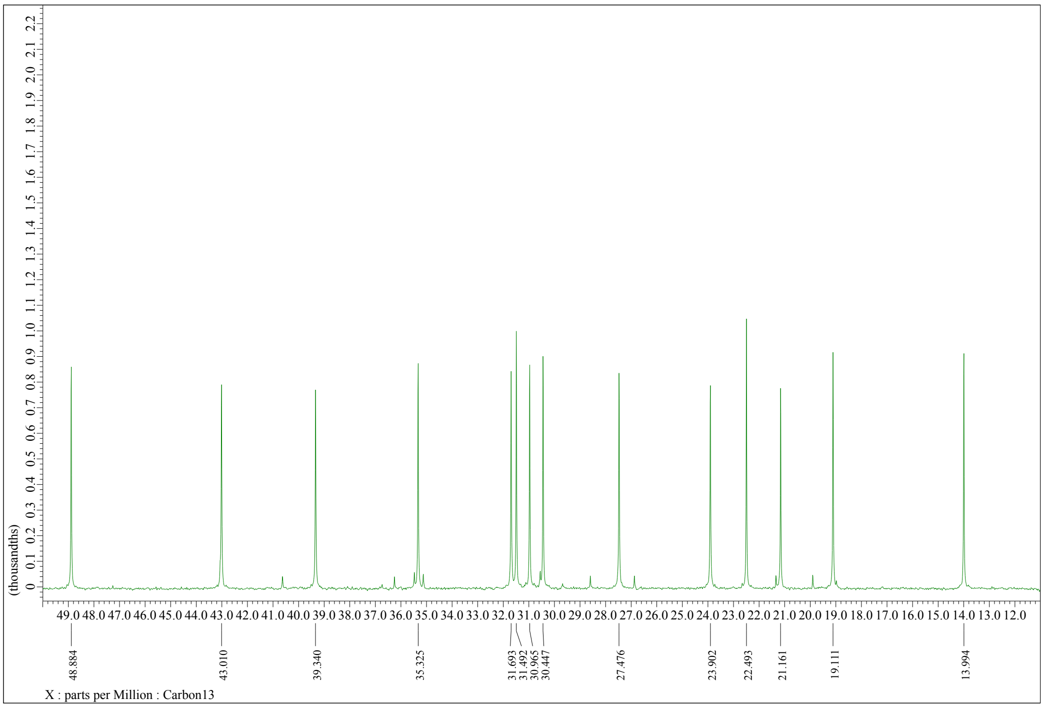
(thousandths)
0
0.1
0.2
0.3
0.4
0.5
0.6
0.7
0.8
0.9
1.0
1.1
1.2
1.3
1.4
1.5
1.6
1.7
1.8
1.9
2.0
2.1
2.2
49.0 48.0 47.0 46.0 45.0 44.0 43.0 42.0 41.0 40.0 39.0 38.0 37.0 36.0 35.0 34.0 33.0 32.0 31.0 30.0 29.0 28.0 27.0 26.0 25.0 24.0 23.0 22.0 21.0 20.0 19.0 18.0 17.0 16.0 15.0 14.0 13.0 12.0
48.884
43.010
39.340
35.325
31.693
31.492
30.965
30.447
27.476
23.902
22.493
21.161
19.111
13.994
X : parts per Million : Carbon13

8. NMR Spectra of **8**

Figure S31. ^{1}H NMR (400 MHz, $CDCl_3$, ppm) of **8**

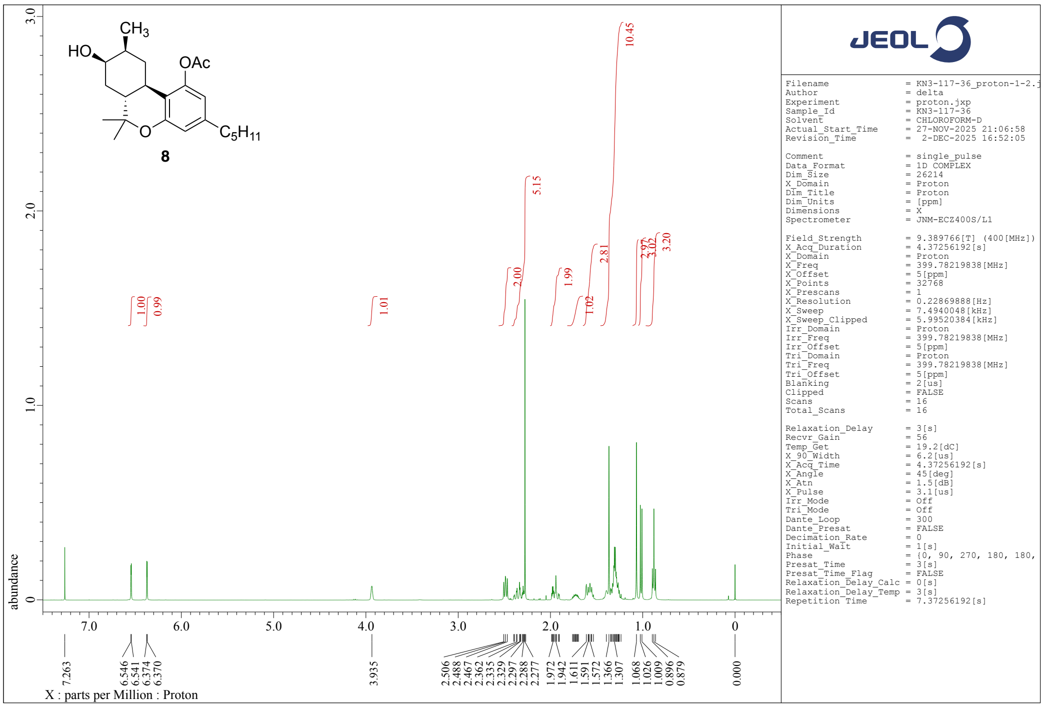

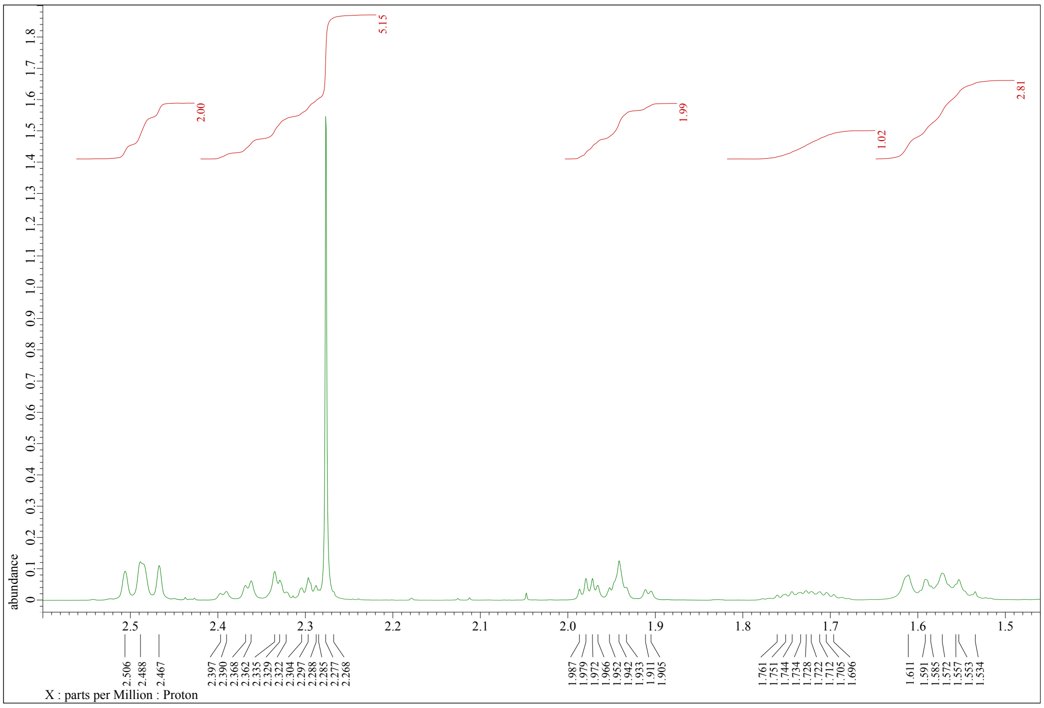

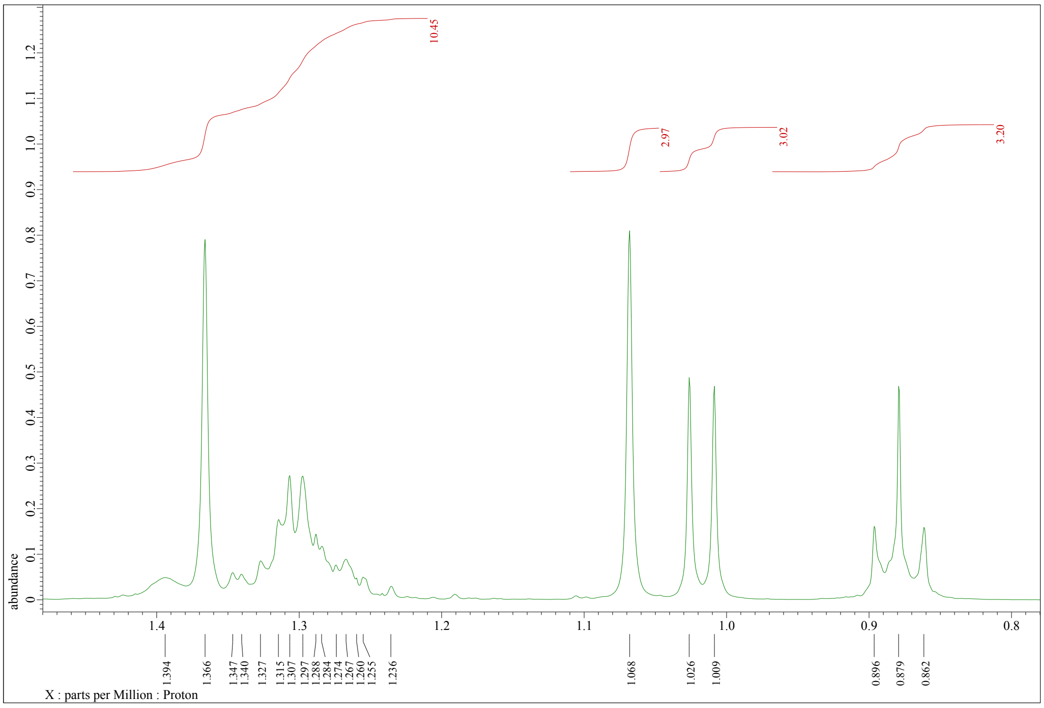

Figure S32. ^{13}C NMR (100 MHz, $CDCl_3$, ppm) of **8**

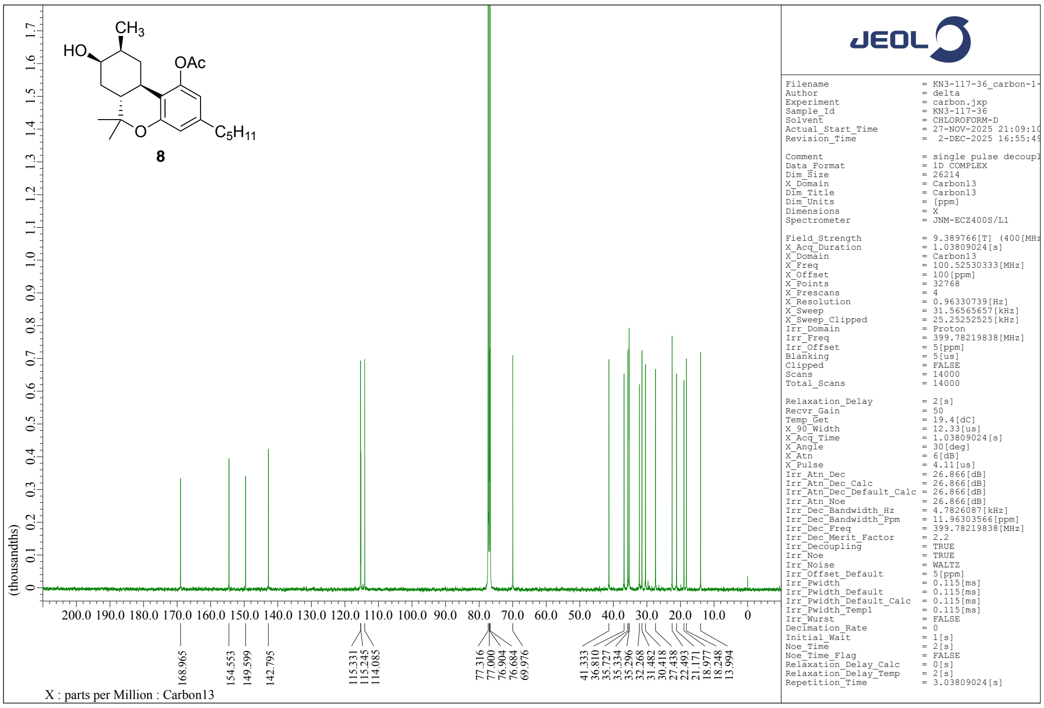

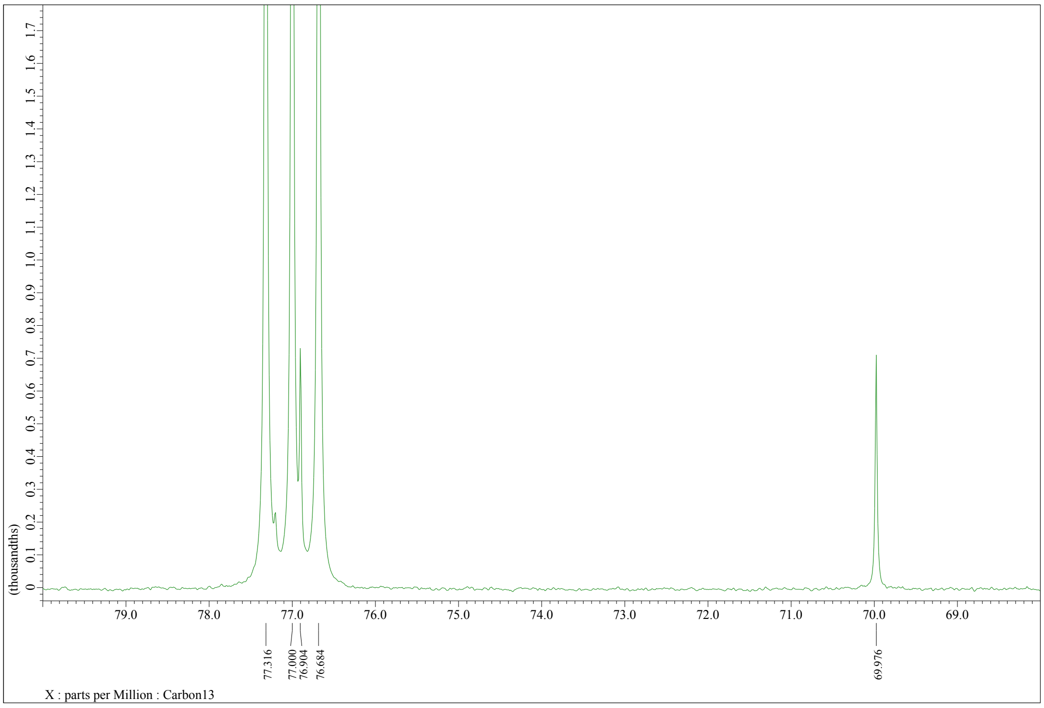
(thousandths)
X : parts per Million : Carbon13
77.316
77.000
76.904
76.684
69.976

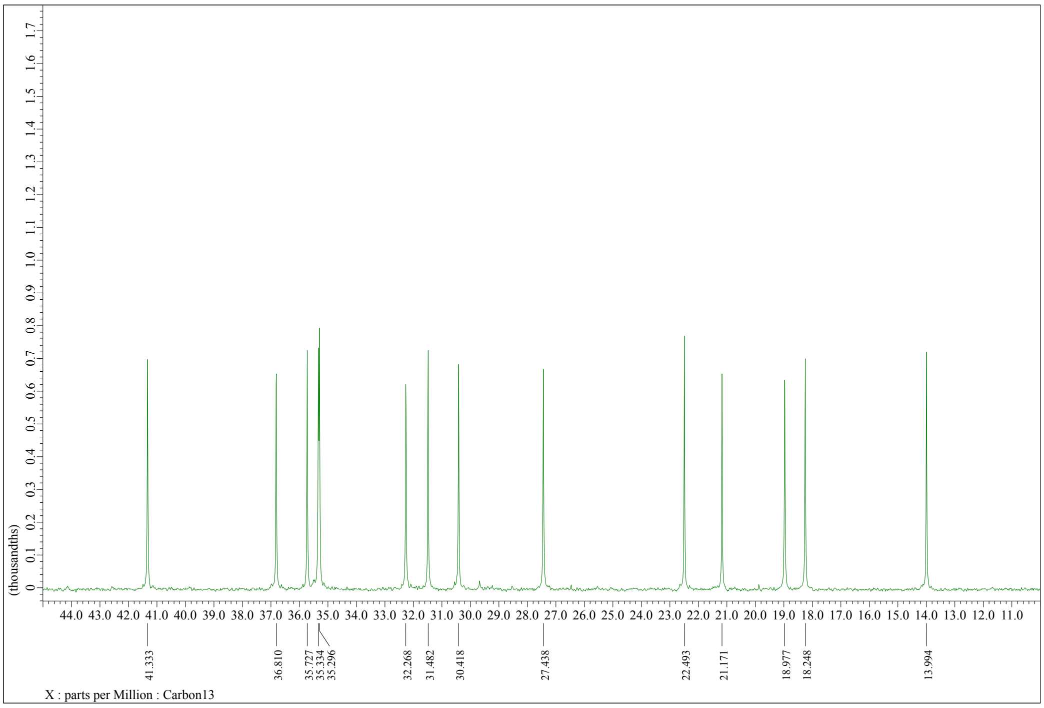
(thousandths)
X : parts per Million : Carbon13
41.333
36.810
35.727
35.334
35.296
32.268
31.482
30.418
27.438
22.493
21.171
18.977
18.248
13.994

9. NMR Spectra of Δ⁸-THC

Figure S33. ^{1}H NMR (400 MHz, $CDCl_3$, ppm) of Δ⁸-THC

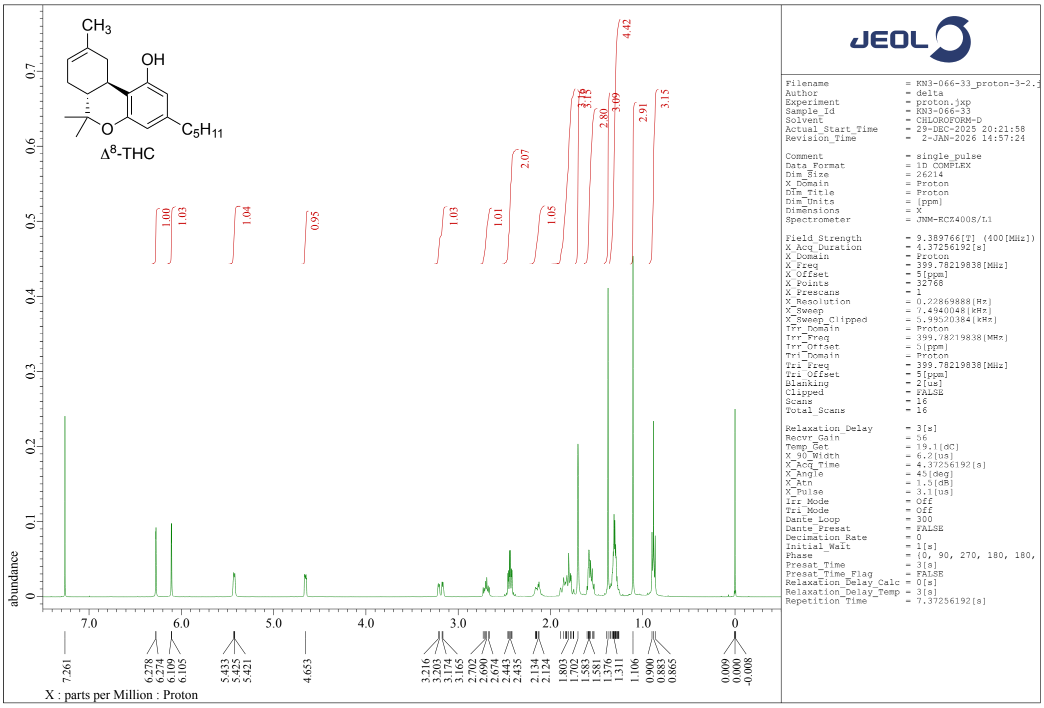

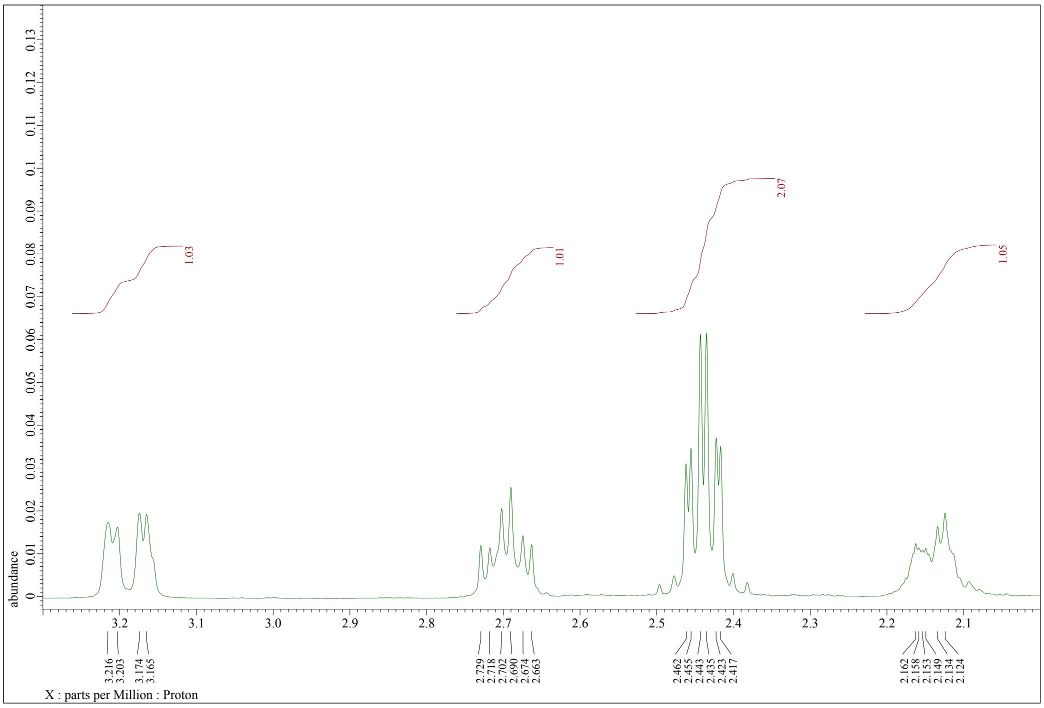

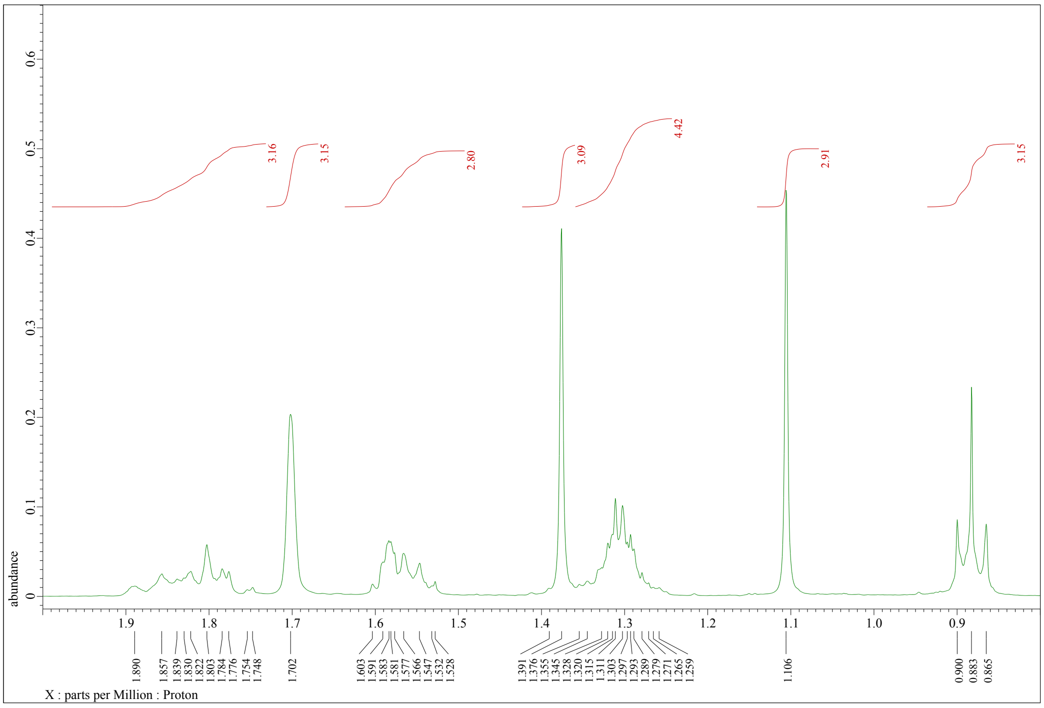

Figure S34. ^{13}C NMR (100 MHz, $CDCl_3$, ppm) of Δ^{8}-THC

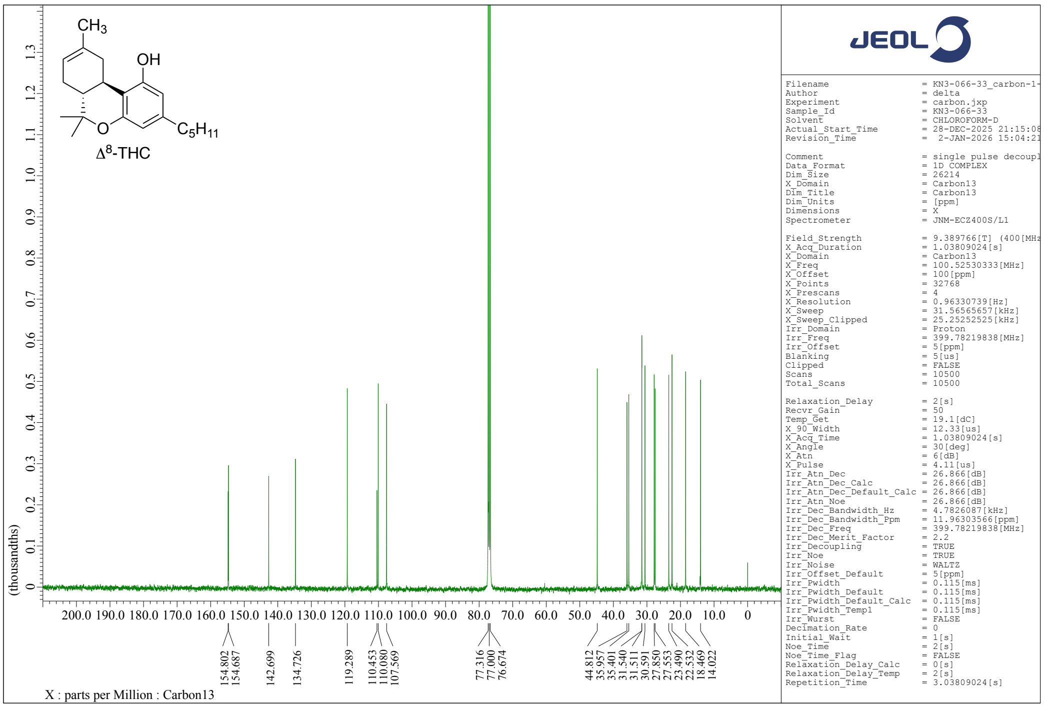

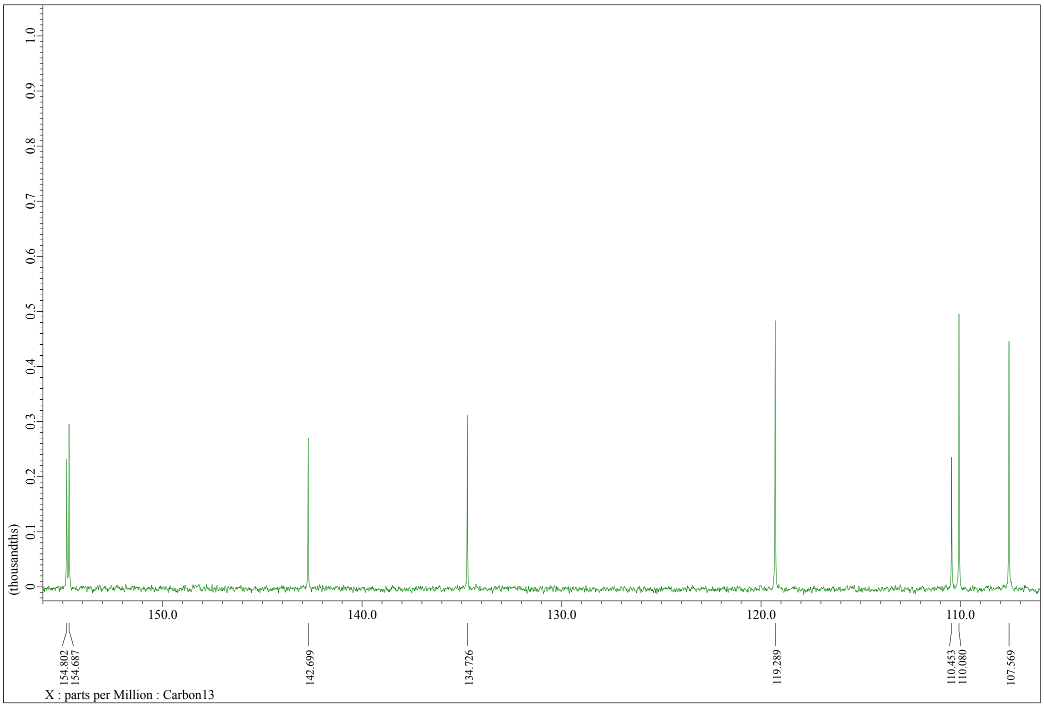

(thousandths)
154.802
154.687
142.699
134.726
119.289
110.453
110.080
107.569
X : parts per Million : Carbon13

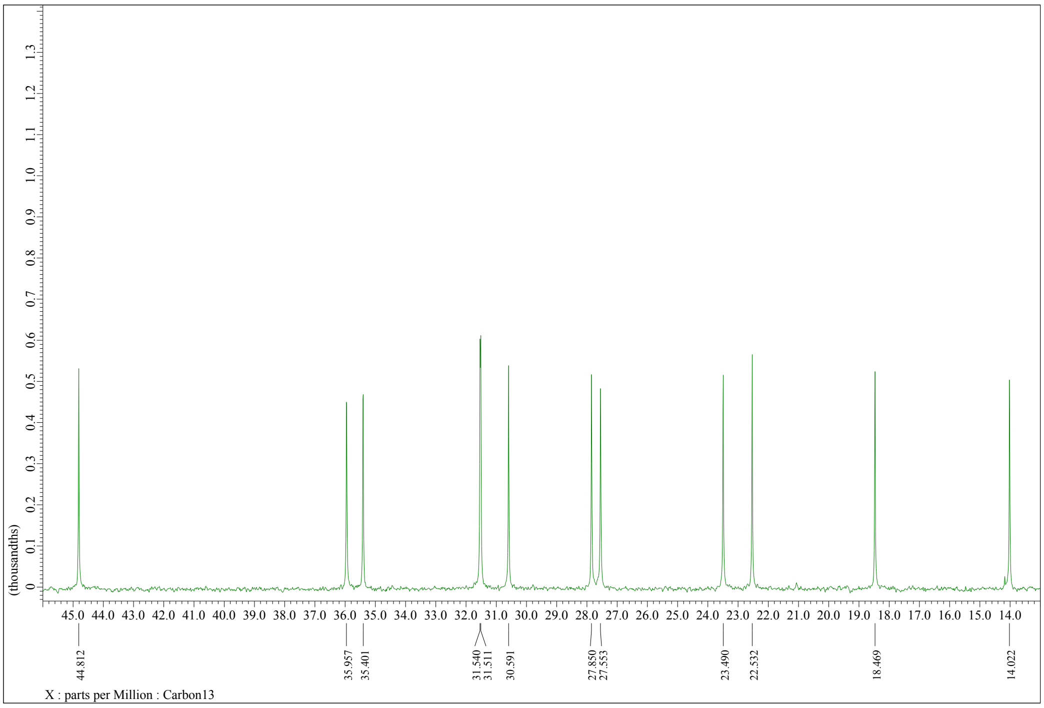

(thousandths)
44.812
35.957
35.401
31.540
31.511
30.591
27.850
27.553
23.490
22.532
18.469
14.022
X : parts per Million : Carbon13

Figure S35. NOESY spectrum of Δ^8-THC

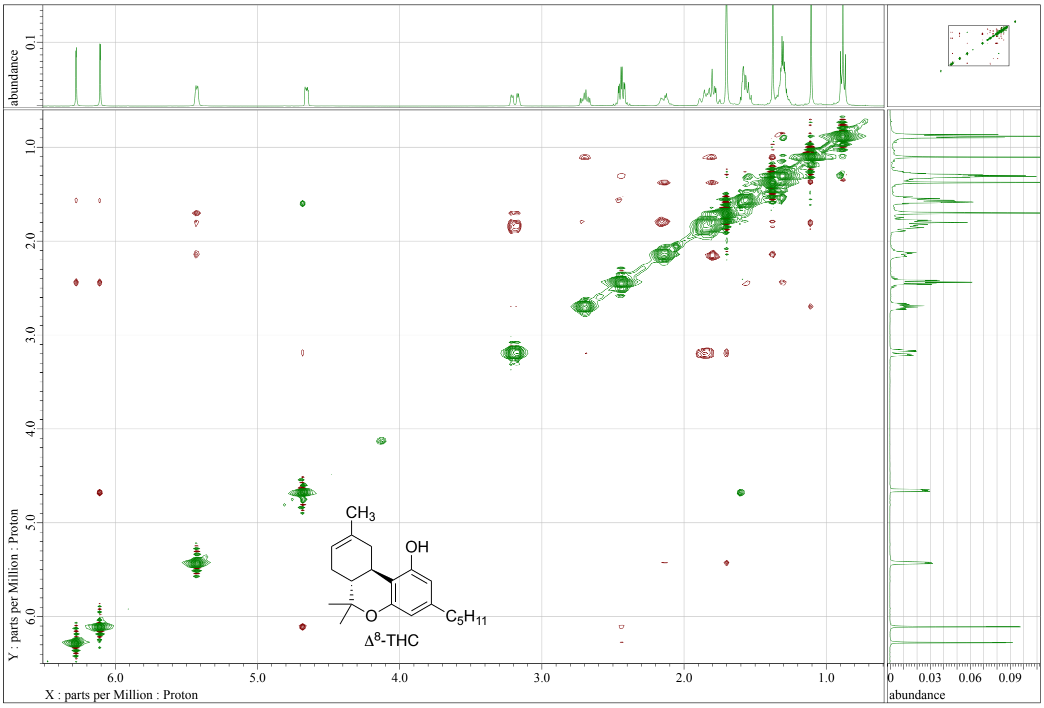

Figure S36. COSY spectrum of Δ^8-THC

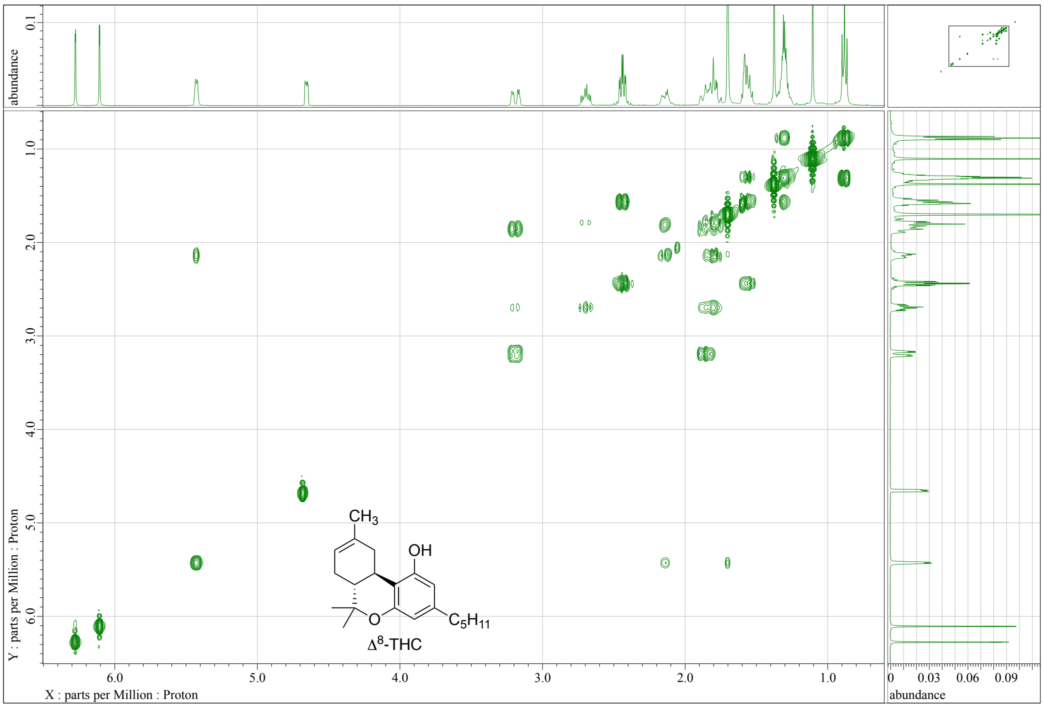

Figure S37. HSQC spectrum of Δ[8]-THC

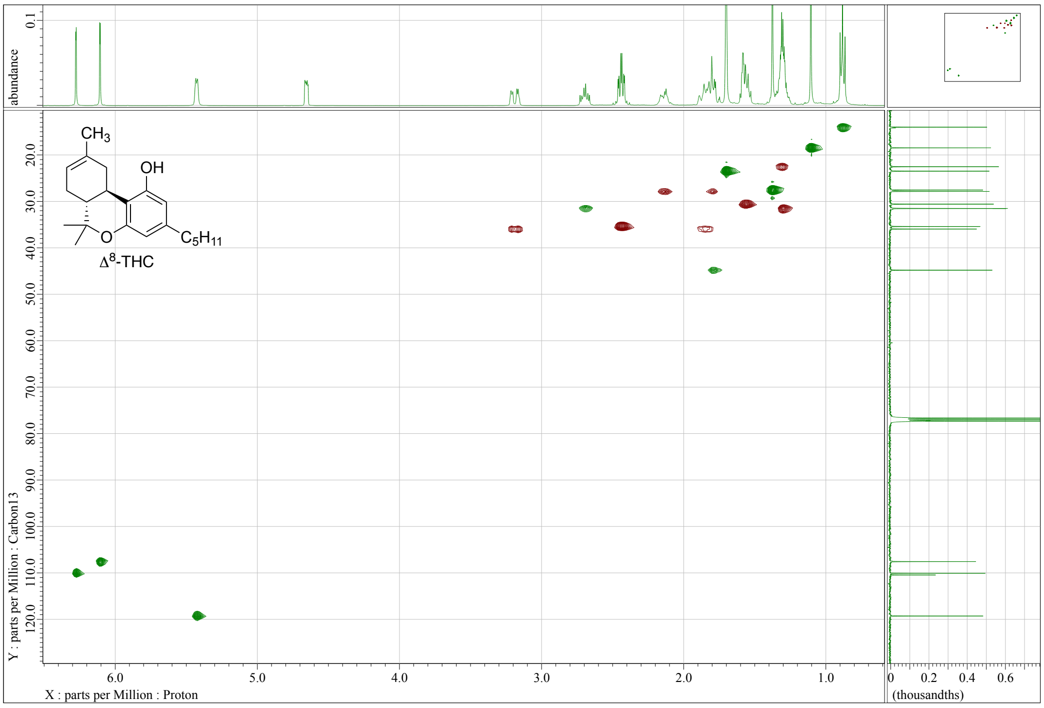

Figure S38. HMBC spectrum of Δ[8]-THC

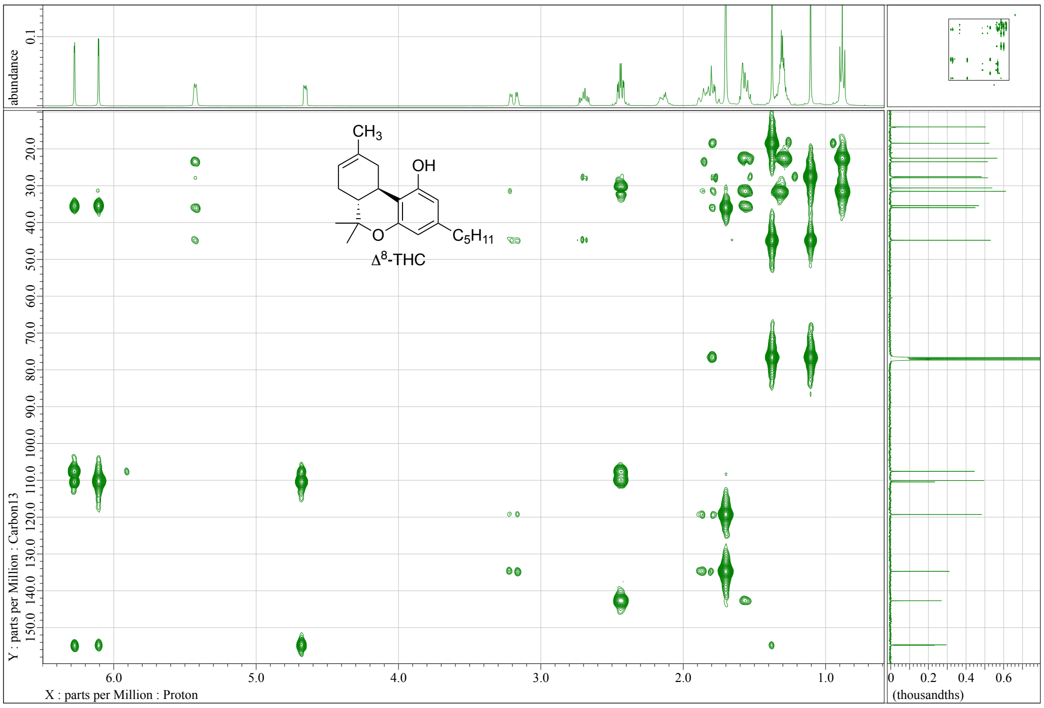

10. NMR Spectra of Δ^8-THC acetate

Figure S39. ^{1}H NMR (400 MHz, $CDCl_3$, ppm) of Δ^8-THC acetate

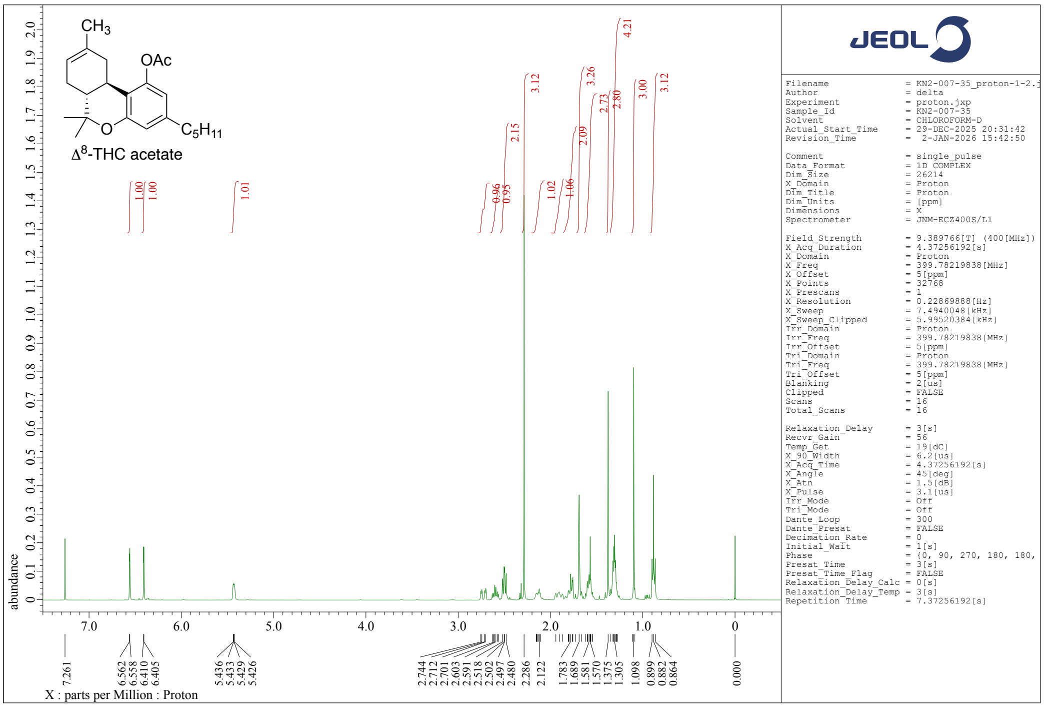

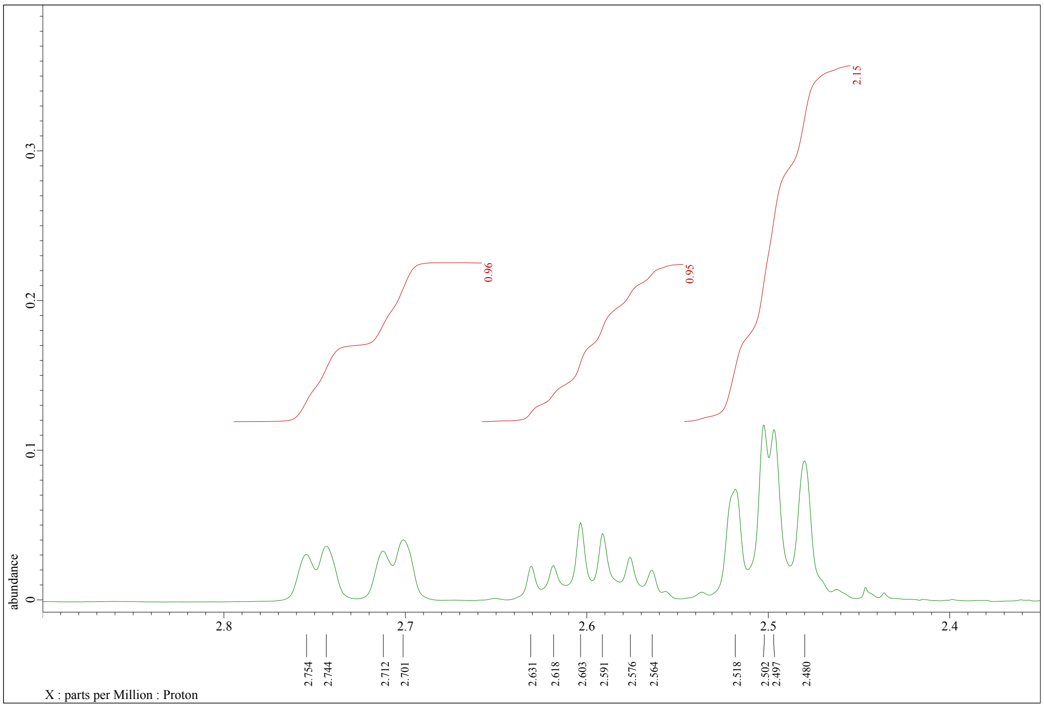

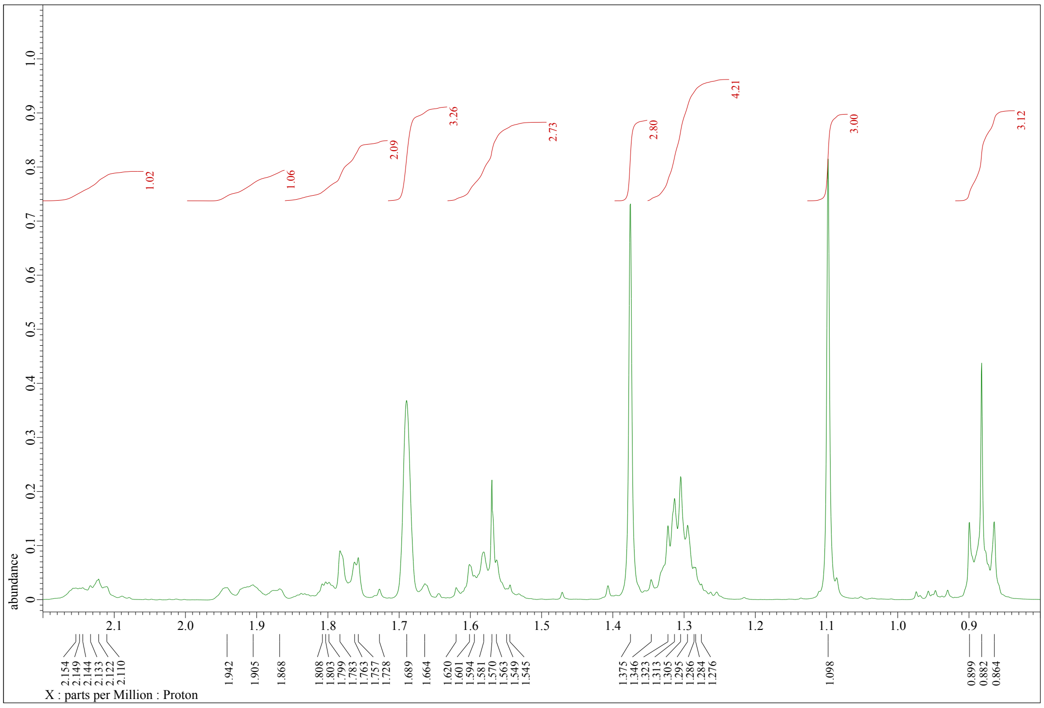

Figure S40. ^{13}C NMR (100 MHz, $CDCl_3$, ppm) of Δ^8-THC acetate

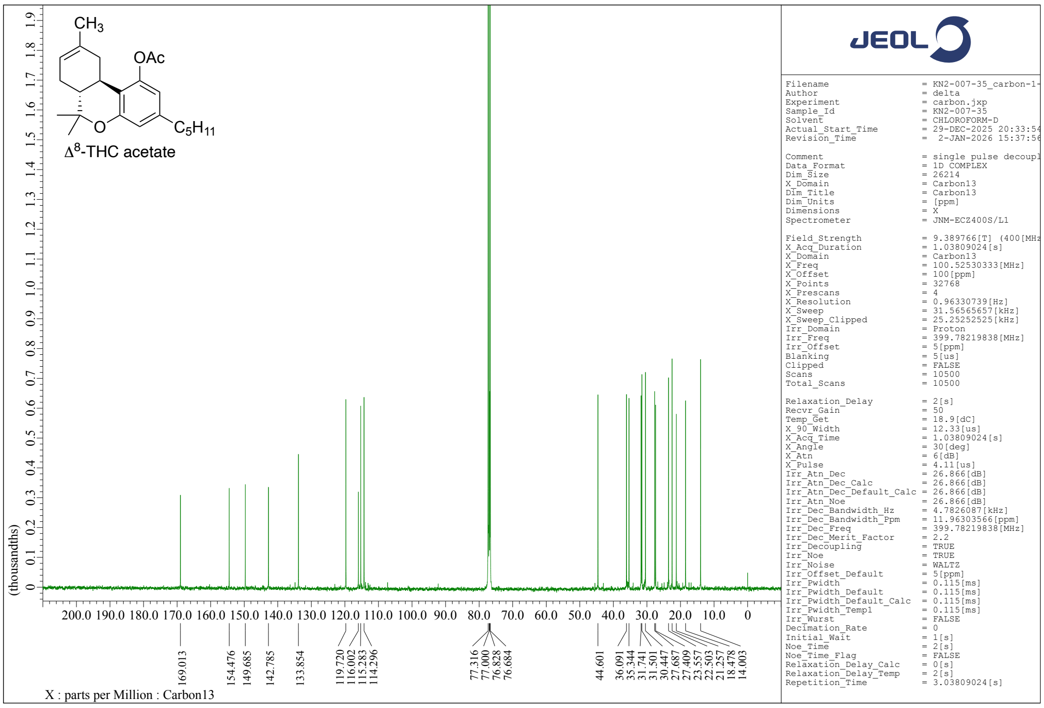

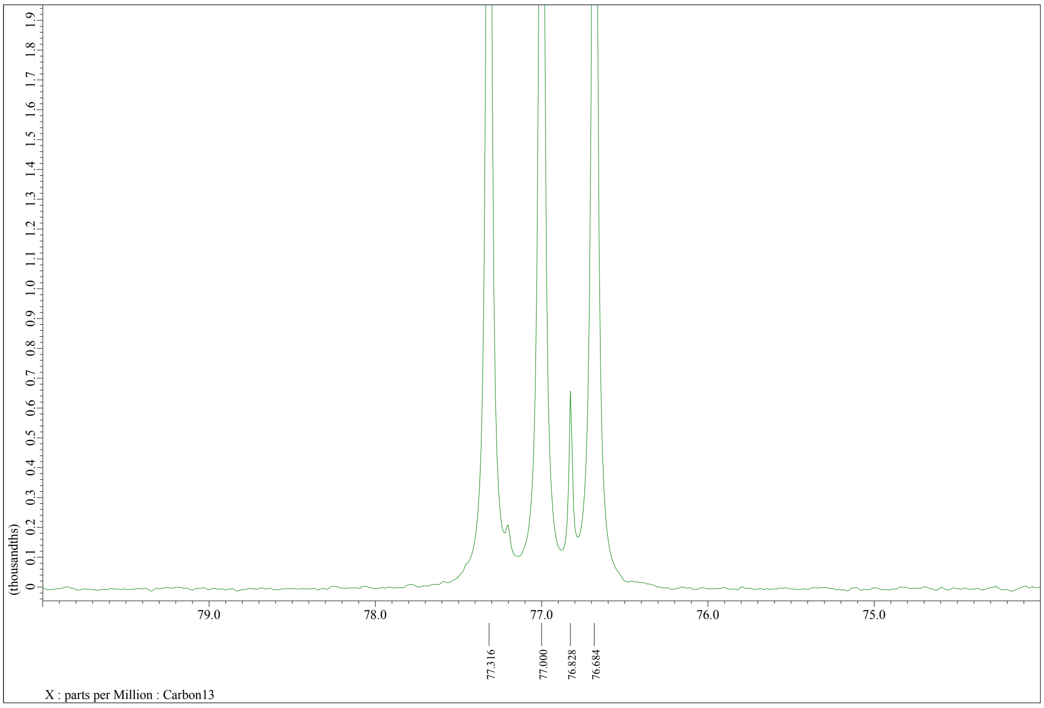
(thousandths)
77.316
77.000
76.828
76.684
X : parts per Million : Carbon13

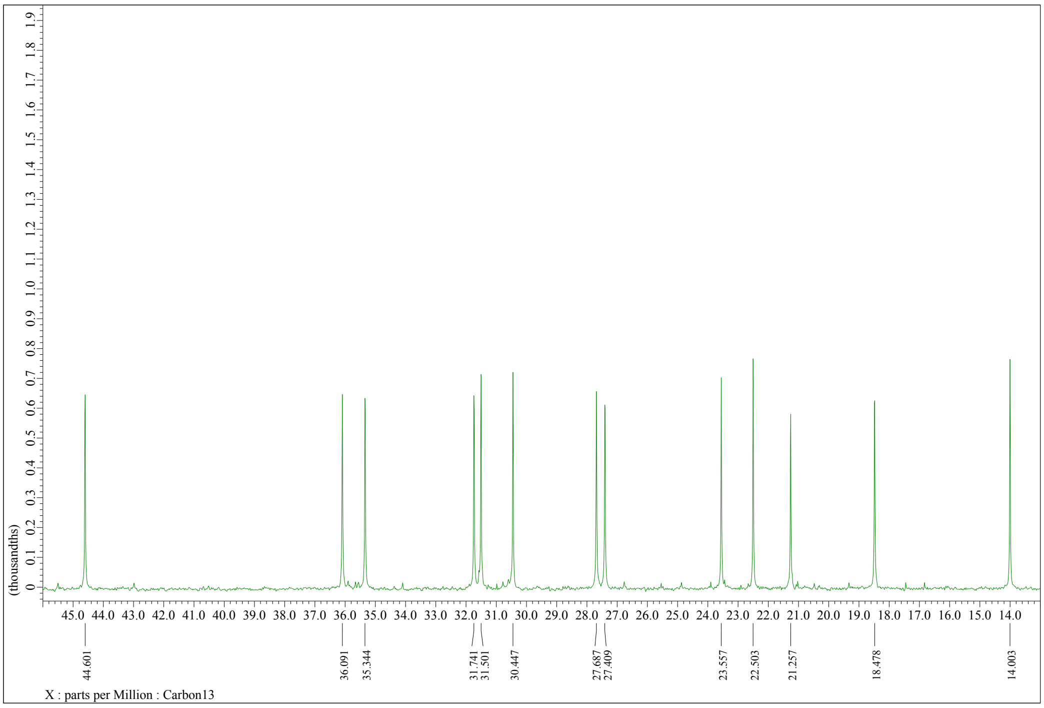
(thousandths)
44.601
36.091
35.344
31.741
31.501
30.447
27.687
27.409
23.557
22.503
21.257
18.478
14.003
X : parts per Million : Carbon13

Figure S41. NOESY spectrum of Δ^8-THC acetate

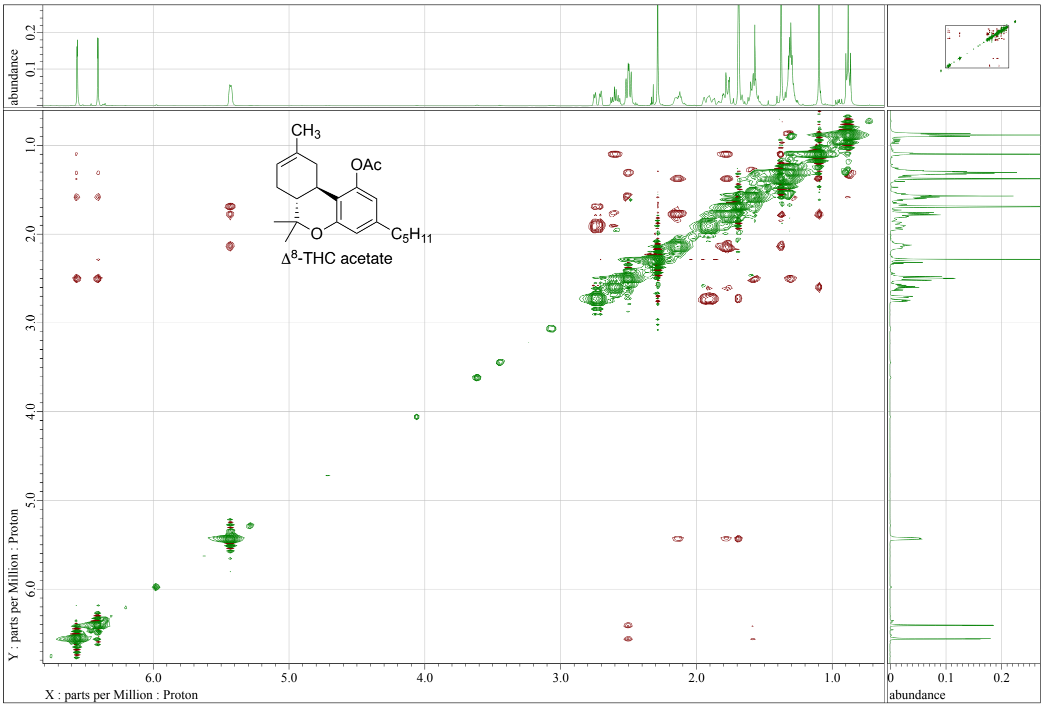

Figure S42. COSY spectrum of Δ^8-THC acetate

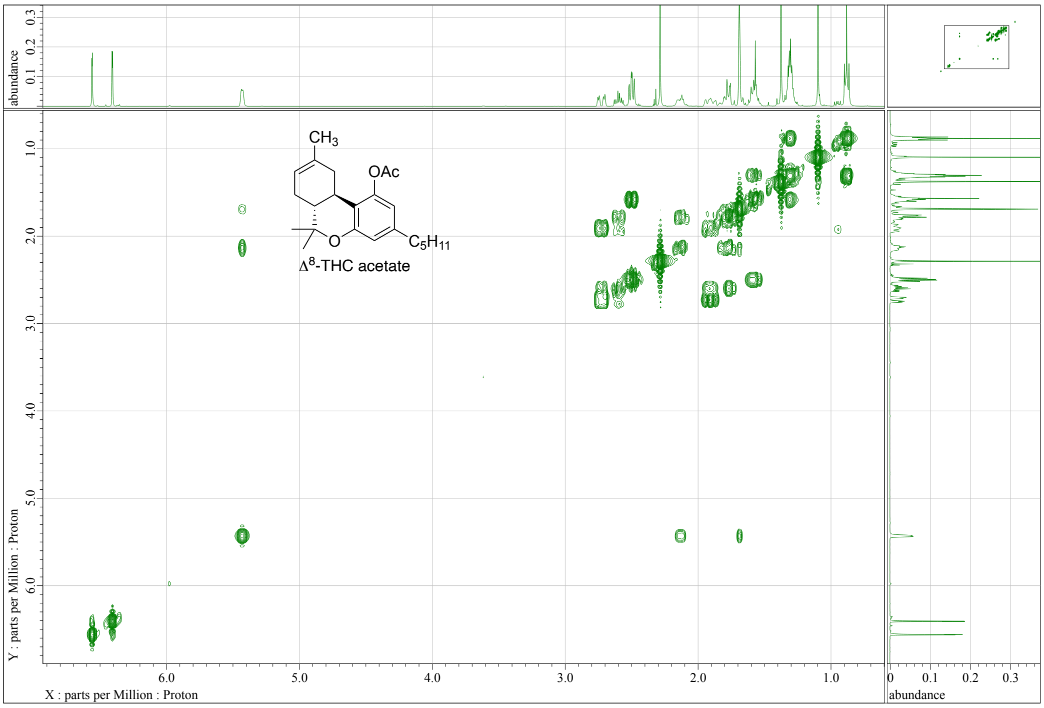

Figure S43. HSQC spectrum of Δ[8]-THC acetate

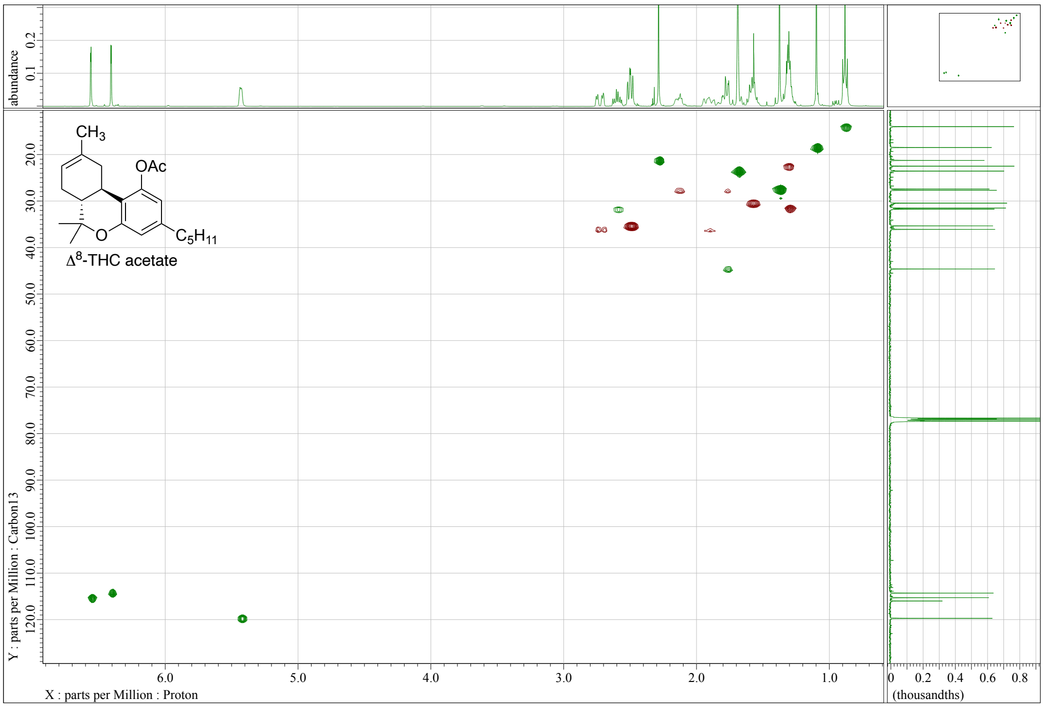

Figure S44. HMBC spectrum of Δ[8]-THC acetate

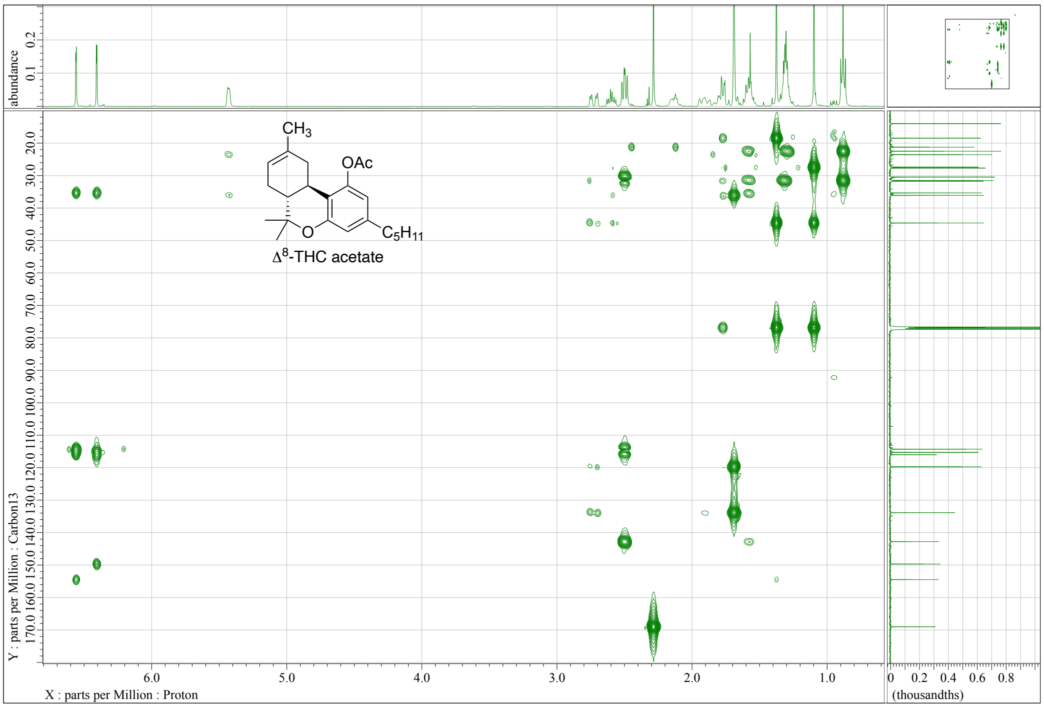

Supplement: Supplementary file 1 [file molecules-31-00289-s001.zip › molecules-4062454-supplementary.pdf]
